# Producer Nutritional Quality Controls Ecosystem Trophic Structure

**DOI:** 10.1371/journal.pone.0004929

**Published:** 2009-03-20

**Authors:** Just Cebrian, Jonathan B. Shurin, Elizabeth T. Borer, Bradley J. Cardinale, Jacqueline T. Ngai, Melinda D. Smith, William F. Fagan

**Affiliations:** 1 Dauphin Island Sea Lab and Department of Marine Sciences, University of South Alabama, Dauphin Island, Alabama, United States of America; 2 Department of Zoology, University of British Columbia, Vancouver, Canada; 3 Department of Zoology, Oregon State University, Corvallis, Oregon, United States of America; 4 Department of Ecology, Evolution and Marine Biology, University of California Santa Barbara, Santa Barbara, California, United States of America; 5 Department of Ecology and Evolutionary Biology, Yale University, New Haven, Connecticut, United States of America; 6 Department of Biology, University of Maryland, College Park, Maryland, United States of America; University of Zurich, Switzerland

## Abstract

Trophic structure, or the distribution of biomass among producers and consumers, determines key ecosystem values, such as the abundance of infectious, harvestable or conservation target species, and the storage and cycling of carbon and nutrients. There has been much debate on what controls ecosystem trophic structure, yet the answer is still elusive. Here we show that the nutritional quality of primary producers controls the trophic structure of ecosystems. By increasing the efficiency of trophic transfer, higher producer nutritional quality results in steeper ecosystem trophic structure, and those changes are more pronounced in terrestrial than in aquatic ecosystems probably due to the more stringent nutritional limitation of terrestrial herbivores. These results explain why ecosystems composed of highly nutritional primary producers feature high consumer productivity, fast energy recycling, and reduced carbon accumulation. Anthropogenic changes in producer nutritional quality, via changes in trophic structure, may alter the values and functions of ecosystems, and those alterations may be more important in terrestrial ecosystems.

## Introduction

The distribution of biomass among producers and consumers, or trophic structure, determines important ecosystem properties such as dynamical stability [1], [2], the abundance of infectious, harvestable or conservation target species [3], and carbon and nutrient recycling and accumulation [4], [5]. For instance, ecosystems with steep trophic pyramids (i.e. high ratios of herbivore-to-producer biomass) maintain high consumer productivity, recycle carbon and nutrients quickly, and accumulate less refractory carbon [3]–[5]. Thus, elucidating the mechanisms that regulate the trophic structure of ecosystems is essential for an understanding of their functions and values, such as the production of food, fiber and biofuel, as well as how anthropogenic environmental perturbations alter those functions and values [5], 6.

Ecologists have long noted that aquatic and terrestrial ecosystems differ greatly in trophic structure. Aquatic ecosystems support larger biomass of herbivores that consume a greater fraction of primary productivity, have smaller biomass of producers, and steeper trophic pyramids than terrestrial ecosystems [5]–[7]. Some authors have proposed that these differences stem from the contrasting nutritional quality of aquatic and terrestrial producers [8]–[11]. Aquatic producers have higher internal concentrations of nutrient-rich compounds and lower concentrations of lignin and other hardy structural compounds. Due to the higher nutritional quality of their diet, aquatic herbivores have faster growth rates and accumulate larger biomass, which leads to higher herbivory rates, smaller producer biomass, and higher herbivore-to-producer biomass ratios in aquatic ecosystems. Higher producer nutritional quality could, through enhanced efficiency of trophic transfer, also lead to the longer food chains typically found in aquatic ecosystems (i.e. higher prominence of secondary and tertiary predators), which in turn could further contribute to higher herbivore-to-producer biomass ratios through alleviation of predation of herbivores by primary carnivores [12], [13].

It remains unknown whether producer nutritional quality is a general control of ecosystem trophic structure and whether it can explain differences in trophic structure within aquatic and terrestrial ecosystems. If this is the case, ecosystems with higher producer nutritional quality should, regardless of whether they are aquatic or terrestrial, feature larger herbivore biomass, higher herbivory rates, smaller producer biomass and steeper trophic structure (i.e. higher herbivore-to-producer biomass ratios). We have already shown that aquatic or terrestrial ecosystems with higher producer nutritional quality support higher rates of herbivory (quantified as % of primary productivity consumed by herbivores; [10], [11]). To test the other predictions, we compiled an unparalleled data set from the literature and other sources that includes measures of aboveground producer biomass, herbivore biomass, and producer nutritional quality expressed as nitrogen and phosphorus content in aboveground producer biomass (i.e. % of producer dry weight) for a broad range of aquatic and terrestrial ecosystems. The data compiled correspond to mean values that integrate the whole ecosystem over at least one year of observations. Our results show that the nutritional quality of primary producers regulates the trophic structure of aquatic and terrestrial ecosystems.

## Results and Discussion

**Figure 1:**
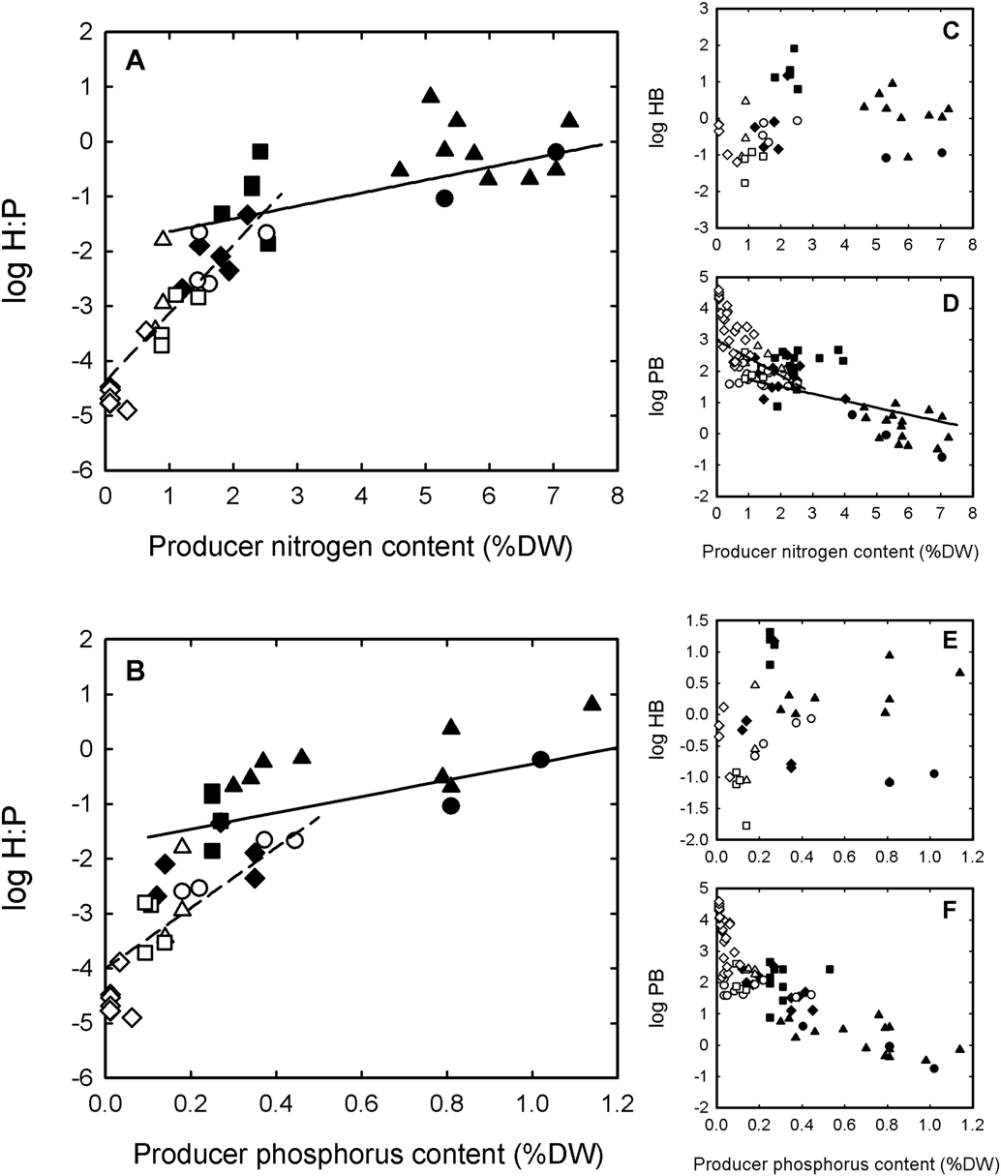
The relationship between trophic structure and producer nutrient content. (A, B) The relationships between the ratio of herbivore-to-producer biomass (H∶P, in g C m^−2^ ∶ g C m^−2^) and producer nitrogen and phosphorus contents. (C, E) The relationships between herbivore biomass (HB, in g C m^−2^) and producer nitrogen and phosphorus contents. (D, F) The relationships between producer biomass (PB, in g C m^−2^) and producer nitrogen and phosphorus contents. Solid symbols denote aquatic systems: triangles, pelagic systems (phytoplankton as dominant producer); circles, sediment flats (benthic microalgae as dominant producer); squares, macroalgal beds; diamonds, submerged grass meadows (seagrasses or freshwater macrophytes as dominant producer). Open symbols denote terrestrial systems: triangles, marshlands; circles, grasslands; squares, tundra heathlands; diamonds, shrublands and forests. Solid and dashed lines depict the associations for aquatic and terrestrial systems respectively. Analyses were done with the Mixed Model ANOVA: log y = μ+b_1_ *producer nutrient content*+b_2_ *system cluster*+b_3_ *producer nutrient content x system cluster*+b_4_ *system type*+*ε*, where y is the given dependent variable, μ is a constant term, *producer nutrient content* is a continuous fixed factor and corresponds to either nitrogen (Figs. 1 A, C, D) or phosphorus (Figs. 1 B, E, F), *system cluster* (aquatic or terrestrial) is a categorical fixed factor, *producer nutrient content x system cluster* denotes the interaction between these two factors, *system type* (four types within aquatic systems and four types within terrestrial systems, with each type corresponding to a different symbol) is a categorical random factor, and *ε* represents unexplained variance. The parameters of the Mixed Model ANOVA were estimated with maximum likelihood. The dependent variable was log transformed to comply with the assumptions of ANOVA. The variable *producer nutrient content* has measurement error, but that error is much smaller than the measurement error in any of the dependent variables, thereby allowing for the use of the model [5], [29]. We tested for the significance of *producer nutrient content*, *system cluster* and their interaction after accounting for the effect of *system type*. The ratio of herbivore-to-producer biomass (H∶P, in g C m^−2^ ∶ g C m^−2^) increased with higher *producer nutrient content* (P<0.001 for *producer nitrogen content*; P<0.001 for *producer phosphorus content*), and the rate of increase was faster for terrestrial than for aquatic systems (P<0.05 for the interaction between *producer nitrogen content* and *system cluster*; P<0.05 for the interaction between *producer phosphorus content* and *system cluster*). Herbivore biomass (HB, in g C m^−2^) was unrelated to *producer nutrient content* (P = 0.68 for *producer nitrogen content*; P = 0.64 for *producer phosphorus content*) within aquatic or terrestrial systems (P = 0.83 for the interaction between *producer nitrogen content* and *system cluster*; P = 0.45 for the interaction between *producer phosphorus content* and *system cluster*). Producer biomass (PB, in g C m^−2^) decreased with higher *producer nutrient content* (P<0.001 for *producer nitrogen content*; P<0.01 for *producer phosphorus content*). The rate of decrease in producer biomass with higher *producer nitrogen content* was faster, albeit only marginally, for terrestrial than for aquatic systems (P = 0.06 for the interaction between *producer nitrogen content* and *system cluster*). Producer biomass also decreased faster with higher *producer phosphorus content* in terrestrial than in aquatic systems, but that difference was driven by the confined distribution of grasslands at the low end of the association for terrestrial systems. In fact, the interaction between *producer phosphorus content* and *system cluster* was significant (P<0.05) when the effect of *system type* was not accounted for in the Mixed Model ANOVA, but not so (P = 0.84) when that effect was accounted for. As such, we do not depict the association lines in Fig. 1F since the lines represent significant differences in slope between system clusters after accounting for the effect of system type, which is not the case here.

The ratio of herbivore-to-producer biomass increased with higher producer nutritional quality both across aquatic and terrestrial ecosystems (Figs. 1A and B). The relationships were significant regardless of whether producer nutritional quality was expressed as nitrogen or phosphorus content. These results demonstrate the herbivore-to-producer biomass ratio in ecosystems is associated with the nutritional quality of primary producers. Because producer nutrient content and primary productivity (quantified as photosynthetic net carbon (C) fixation per square meter per year) are unrelated when a wide range of aquatic or terrestrial ecosystems are compared (i.e. ecosystems with nutritionally-poor producers can be little or very productive, and the same is true for ecosystems with nutritionally-rich producers [10], [11]), herbivore-to-producer biomass ratios are unrelated to primary productivity across the wide range of aquatic and terrestrial ecosystems examined here (supporting information (SI) Fig. S1). Thus, in contrast to what some authors have previously suggested based on more restricted data sets [14], [15], primary productivity is not a strong indicator of differences in trophic structure across aquatic and terrestrial ecosystems.

Producer biomass decreased as producer nutritional quality increased across aquatic and terrestrial ecosystems (Figs. 1D and F). Our prior work indicates this trend is partially due to higher herbivory rates (quantified as % of primary productivity consumed by herbivores) in ecosystems with higher producer nutritional quality [10], [16], with the rest of the decrease in producer biomass being attributable to higher rates of producer natural mortality [17]. Herbivore biomass did not increase with higher producer nutritional quality across aquatic and terrestrial ecosystems (Figs. 1C and E). The reason for this resides on the large variability in primary productivity that occurs for any given producer nutrient content both across aquatic and terrestrial ecosystems [10], [11]. That variability overwhelms the increasing trend in the percent consumed with higher producer nutrient content, such that absolute consumption (which corresponds to the product between primary productivity and the fraction consumed and it is expressed in g C consumed per square meter per year) also varies largely for any given producer nutrient content [10], [11]. Large variability in absolute consumption for any given producer nutrient content implies large variability in the absolute transfer of producer biomass to herbivores, which overrides any increases in herbivore biomass that may result from higher growth rates in ecosystems with higher producer nutritional quality. This leads to the independence between herbivore biomass and producer nutrient content across the broad range of ecosystems compared.

Our results also show that the shift in trophic structure as producer nutrient quality increases is more pronounced for terrestrial than for aquatic ecosystems. The increase in the percentage of primary productivity consumed [11], the decrease in producer biomass, and the increase in the ratio of herbivore-to-producer biomass with higher producer nutritional quality are all faster in terrestrial than in aquatic ecosystems (Fig. 1). This suggests that terrestrial herbivores suffer more severe nutritional limitation than their aquatic counterparts, which is consistent with the larger imbalance between herbivore nutritional requirements and diet nutrient availability in terrestrial than in aquatic ecosystems [18], [19]. Therefore, increases in producer nutrient content should relieve the nutritional limitation of herbivores, stimulate their metabolic and growth rates, and, as observed with our results, increase herbivory rates, reduce producer biomass and increase the ratio of herbivore-to-producer biomass to a greater extent in terrestrial than in aquatic ecosystems. Indeed, the steep increase in the ratio of herbivore-to-producer biomass observed from woody (i.e. dominated by shrubs and trees) to herbaceous (i.e. dominated by grasses and forbs) ecosystems points to stringent nutritional limitation of herbivores in the former due to low nutrient contents in the structural compounds of the producers.

**Figure 2:**
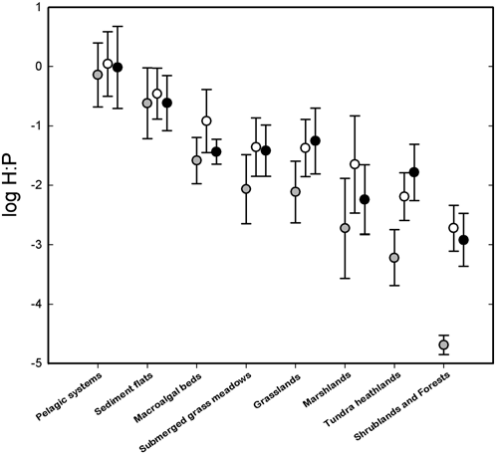
The ratio (mean±SD) of herbivore-to-producer biomass (H∶P, in g element m^−2^ ∶ g element m^−2^) in aquatic and terrestrial systems. Gray, white and black circles correspond to carbon, nitrogen and phosphorus units, respectively. Ratios were analyzed with a two way ANOVA with element (carbon, nitrogen and phosphorus) and system type as the two factors after log-transformation to comply with the assumptions of ANOVA. Ratios varied among elements (P<0.001) and also among system types (P<0.001). Most importantly, the differences among elements depended on the system type considered (P<0.05 for the interaction between element and system type) indicating that, for terrestrial systems, ratios expressed in carbon units tended to be lower than ratios expressed in nitrogen or phosphorus units, but not for aquatic systems.

To offer further support for more severe nutritional limitation of herbivores in terrestrial than in aquatic ecosystems, we compared the ratio of herbivore-to-producer biomass expressed in units of carbon, nitrogen or phosphorus. Modeling and empirical studies suggest that herbivores that are more severely limited by the nutritional quality of their diet tend to retain nutrients in their bodies to a greater extent [19]–[22]. Thus, ratios of herbivore- to-producer biomass in terrestrial ecosystems should be higher when expressed in nitrogen or phosphorus units than when expressed in carbon units, whereas such differences should be less pronounced in aquatic ecosystems. We compiled values of body nutrient content (i.e., nitrogen or phosphorus as % body dry weight) for the herbivores in a subset of the studies compiled (Data Sets S1) and plotted the ratios of herbivore-to-producer biomass in units of carbon, nitrogen and phosphorus for diverse aquatic and terrestrial ecosystems (Fig. 2). As expected, ratios in terrestrial ecosystems tended to be higher in terms of nitrogen and phosphorus than carbon units, but this was not the case for aquatic ecosystems. This supports that terrestrial herbivores are more severely limited by the nutritional quality of their diet than aquatic herbivores.

Our findings suggest that the alleviation of nutritional limitation of herbivores with higher producer nutrient content seems to be a major factor controlling the efficiency of trophic transfer in aquatic and terrestrial ecosystems. Higher producer nutrient contents, most likely by stimulating the metabolic and growth rates of herbivores, increase the intensity of herbivory, which leads to reduced producer biomass, higher ratios of herbivore-to-producer biomass, and steeper ecosystem trophic structure, with these changes being more pronounced in terrestrial than in aquatic ecosystems. Thus, producer nutritional quality is an important determinant of the functions and values of ecosystems through its impacts on trophic structure. Our results explain why ecosystems with higher producer nutritional quality often feature higher consumer productivity [23], faster recycling of energy and materials through the food web [10], [24], and smaller accumulation of refractory carbon [25]. Anthropogenic activities are altering the nutritional quality of producers in ecosystems worldwide [26], [27]. Such changes may propagate upward through the trophic structure of ecosystems to alter their functions and values, and those alterations may be more severe in terrestrial than in aquatic ecosystems.

## Materials and Methods

We searched the literature extensively to compile studies that represented a wide range of aquatic (from pelagic to sediment flats to submerged macrophyte beds) and terrestrial (from marshlands to grasslands to forests) systems. Some systems have been studied more than others and were better represented in the literature, but even the values obtained for the less studied systems, albeit not numerous, covered a wide spread. Thus our compilation (Data Sets S1) reflects the availability of information in the scientific record and the inclusion of more values for the less studied systems, had they existed, would likely leave our conclusions unaltered.

We only included studies that met three conditions. First, the system was not deliberately impacted by human activities and, thus, was mostly representative of natural conditions. Second, since our analysis compares systems, and not populations or individuals, we only considered studies that included all, or at least the most abundant, producers and herbivores in the system. Third, to eliminate any effects of seasonality on our results, we only accepted studies that provided data for an entire year or at least the entire growing season for annual producers.

Within-study variability was only known for a small fraction of the studies culled and, thus, we could not weight the final mean values compiled by the inverse of their variance as it is recommended for meta-analyses [28]. However, all the mean values compiled integrate whole systems over at least one year and, thus, should have high and similar reliability. Furthermore, our results, being based on a large number of mean values, have high statistical power. Therefore, our conclusions are robust despite not accounting for within-study variability [11], [28].

## Supporting Information

Figure S1 The relationship between the ratio of herbivore-to-producer biomass (H∶P, in g C m−2 ∶ g C m−2) and primary productivity. Solid symbols denote types of aquatic systems: triangles, pelagic systems (phytoplankton as dominant producer); circles, sediment flats (benthic microalgae as dominant producer); squares, macroalgal beds; diamonds, submerged grass meadows (seagrasses or freshwater macrophytes as dominant producer). Open symbols denote types of terrestrial systems: triangles, marshlands; circles, grasslands; squares, tundra heathlands; diamonds, shrublands and forests. The solid and dashed lines represent the mean ratios for aquatic and terrestrial systems respectively. The analysis was done with the mixed Model ANOVA: log H∶P = μ+b1 primary productivity+b2 system cluster+b3 primary productivity x system cluster+b4 system type+ε, where μ is a constant term, primary productivity is a continuous fixed factor, system cluster (aquatic or terrestrial) is a categorical fixed factor, primary productivity x system cluster denotes the interaction between these two factors, system type (four types within aquatic systems and four types within terrestrial systems, with each type corresponding to a different symbol) is a categorical random factor, and ε represents unexplained variance. The parameters of the Mixed Model ANOVA were estimated with maximum likelihood. The dependent variable (i.e. H∶P) was log transformed to comply with the assumptions of ANOVA. The variable primary productivity has measurement error but this model is adequate because that measurement error is much smaller than the measurement error in the dependent variable [5], [29]. We tested for the significance of primary productivity, system cluster and their interaction after accounting for the effect of system type. The ratio was unrelated to primary productivity (P = 0.60 for primary productivity) regardless of what system cluster was considered (P = 0.26 for the interaction).
(8.43 MB TIF)

Data Sets S1 Data Sets for Figures 1 and 2 in Main Text and Figure S1.
(0.49 MB PDF)
