# Supplementary material for: Producer Nutritional Quality Controls Ecosystem Trophic Structure
Source: PLoS One. 2009 Mar 20;4(3):e4929. doi: 10.1371/journal.pone.0004929 (PMC2654170; doi:10.1371/journal.pone.0004929)
Supplement: Data Sets S1 — Data Sets for Figures 1 and 2 in Main Text and Figure S1. (0.49 MB PDF) [file pone.0004929.s002.pdf]

Supporting Information: Data Set for Figure 1 in Main Text and Figure S1

reference	community type	ecosystem typ	PB (gCm-2)	NPP (gC m-2 y-1	H (gC m-2 y-1)	%NPP consumed	HB (gC m-2)	Nbiomass (%D	Pbiomass (%DW)
Arndt et al. 1993, Behrendt 1990	communities of freshwater phytoplankton	aquatic	.	204.400000	153.300000	75.000000	.	6.300000	.
Cloern et al 1987	communities of freshwater phytoplankton	aquatic	3.625000	1976.110000	1758.935000	89.009974	.	.	.
Coveney et al. 1977	communities of freshwater phytoplankton	aquatic	5.080000	331.785000	84.680000	25.522552	2.16	.	.
Cushing 1976	communities of freshwater phytoplankton	aquatic	1.811000	275.575000	124.465000	45.165563	.	.	.
Cyr and Pace 1993	communities of freshwater phytoplankton	aquatic	.	75.10768486	.	.	0.112413814	.	.
Cyr and Pace 1993	communities of freshwater phytoplankton	aquatic	.	65.90231627	.	.	0.60988213	.	.
Cyr and Pace 1993	communities of freshwater phytoplankton	aquatic	.	93.39505225	.	.	1.062256787	.	.
Cyr and Pace 1993	communities of freshwater phytoplankton	aquatic	.	59.52995878	.	.	0.121687484	.	.
Cyr and Pace 1993	communities of freshwater phytoplankton	aquatic	25.510000	1813.33	.	.	1.8	.	.
Cyr and Pace 1993	communities of freshwater phytoplankton	aquatic	3.920000	724.3277317	.	.	10.30658424	.	.
Cyr and Pace 1993	communities of freshwater phytoplankton	aquatic	.	438.7976336	.	.	2.346884996	.	.
Cyr and Pace 1993	communities of freshwater phytoplankton	aquatic	.	146.525144	.	.	0.456054138	.	.
Cyr and Pace 1993	communities of freshwater phytoplankton	aquatic	.	171.9152696	.	.	0.794328247	.	.
Cyr and Pace 1993	communities of freshwater phytoplankton	aquatic	.	258.2110605	.	.	0.315036347	.	.
Cyr and Pace 1993	communities of freshwater phytoplankton	aquatic	.	448.4646609	.	.	0.56340368	.	.
Cyr and Pace 1993	communities of freshwater phytoplankton	aquatic	.	355.4507481	.	.	1.149888535	.	.
Cyr and Pace 1993	communities of freshwater phytoplankton	aquatic	.	296.42388	.	.	1.180677137	.	.
Cyr and Pace 1993	communities of freshwater phytoplankton	aquatic	.	441.996598	.	.	1.03455624	.	.
Cyr and Pace 1993	communities of freshwater phytoplankton	aquatic	.	277.6651276	.	.	0.773614492	.	.
Cyr and Pace 1993	communities of freshwater phytoplankton	aquatic	.	260.0934954	.	.	0.660194859	.	.
Cyr and Pace 1993	communities of freshwater phytoplankton	aquatic	.	236.6568305	.	.	0.626211903	.	.
Garnier and Mourelatos 1991	communities of freshwater phytoplankton	aquatic	1.408000	169.360000	113.150000	66.810345	0.534402966	.	.
Gliwicz and Hillbricht-Ilkowska 1972	communities of freshwater phytoplankton	aquatic	0.140000	121.545000	48.180000	39.639640	.	.	.
Gliwicz and Hillbricht-Ilkowska 1972	communities of freshwater phytoplankton	aquatic	0.107000	123.005000	28.214500	22.937685	.	.	.
Gliwicz and Hillbricht-Ilkowska 1972	communities of freshwater phytoplankton	aquatic	0.361000	190.895000	158.775000	83.173996	.	.	.
Gulati 1975	communities of freshwater phytoplankton	aquatic	4.800000	146.000000	124.100000	85.000000	.	.	.
Gulati 1975	communities of freshwater phytoplankton	aquatic	8.000000	547.500000	354.050000	64.666667	.	.	.
Gulati 1984	communities of freshwater phytoplankton	aquatic	4.700000	867.250000	.	.	0.760000	.	.
Jensen and Schwaerter 1988	communities of freshwater phytoplankton	aquatic	7.600000	840.960000	262.800000	31.250000	.	.	.
Jespersen et al. 1988	communities of freshwater phytoplankton	aquatic	6.900000	218.635000	102.930000	47.078464	2	4.6	0.34
Jewson et al. 1981	communities of freshwater phytoplankton	aquatic	7.000000	186.515000	73.000000	39.138943	.	.	.
Lampert 1978	communities of freshwater phytoplankton	aquatic	2.469600	379.600000	343.100000	90.384615	.	.	.
Nakanishi et al. 1992	communities of freshwater phytoplankton	aquatic	1.733000	430.700000	231.045000	53.644068	1.0167	5.76	0.37
Tilzer 1984	communities of freshwater phytoplankton	aquatic	1.447000	296.380000	222.650000	75.123153	.	.	.
Urabe et al. 1995	communities of freshwater phytoplankton	aquatic	.	294.190000	220.640000	74.999150	.	4.910000	0.314000
Vadstein et al 1989	communities of freshwater phytoplankton	aquatic	4.305000	208.415000	117.530000	56.392294	.	.	.
Weisse et al 1990	communities of freshwater phytoplankton	aquatic	5.660000	448.950000	212.065000	47.235772	1.1788	6.63	0.3
Baird and Ulanowicz 1989	communities of marine phytoplankton	aquatic	3.500000	337.179700	73.000000	21.650176	1.062	7.04	0.79
Baird and Ulanowicz 1993	communities of marine phytoplankton	aquatic	0.413000	11.553710	7.665000	66.342326	0.0841	5.98	0.81
Baird and Ulanowicz 1993	communities of marine phytoplankton	aquatic	2.650000	37.726400	17.885000	47.407121	1.8045	5.3	0.46
Baird and Ulanowicz 1993	communities of marine phytoplankton	aquatic	3.730000	120.252900	94.900000	78.917016	8.7189	5.49	0.81
Baird and Ulanowicz 1993	communities of marine phytoplankton	aquatic	0.750000	103.747600	19.710000	18.998030	1.7398	7.25	0.81
Baird and Ulanowicz 1993 (phytoplankton average)	communities of marine phytoplankton	aquatic	.	.	.	.	.	5.490000	0.810000
Bienfag and Zieman 1992	communities of marine phytoplankton	aquatic	.	30.652700	.	.	.	.	.
Bienfag and Zieman 1992	communities of marine phytoplankton	aquatic	.	120.252900	.	.	.	.	.
Bishop et al 1977	communities of marine phytoplankton	aquatic	0.800000	54.750000	.	.	.	5.800000	0.700000
Brown et al 1991	communities of marine phytoplankton	aquatic	8.250000	1266.032000	.	.	4.150000	.	.
Chavez et al. 1991	communities of marine phytoplankton	aquatic	.	426.779900	248.200000	58.156441	.	.	.
Durbin et al. 1975	communities of marine phytoplankton	aquatic	2.400691	507.350000	337.387750	66.500000	.	5.800000	.
Frost 1991	communities of marine phytoplankton	aquatic	1.010000	200.421500	160.600000	80.131124	.	.	.
Garber 1984	communities of marine phytoplankton	aquatic	.	308.000000	.	.	.	.	.
Harrison et al. 1993	communities of marine phytoplankton	aquatic	.	0.400843	0.302950	75.578219	.	.	.
Holligan et al. 1984	communities of marine phytoplankton	aquatic	7.000000	532.885400	.	.	.	.	.
Holligan et al. 1984	communities of marine phytoplankton	aquatic	0.800000	122.610800	.	.	.	.	.
Holligan et al. 1984	communities of marine phytoplankton	aquatic	30.000000	530.527500	.	.	.	.	.
Holligan et al. 1993	communities of marine phytoplankton	aquatic	.	42.442200	.	.	.	.	.
Holligan et al. 1993	communities of marine phytoplankton	aquatic	.	87.242300	.	.	.	.	.
Holligan et al. 1993	communities of marine phytoplankton	aquatic	.	54.231700	5.000500	9.220622	.	.	.
Holligan et al. 1993	communities of marine phytoplankton	aquatic	.	73.094900	.	.	.	.	.
Iriarte et al. 1991	communities of marine phytoplankton	aquatic	9.100000	1322.781900	.	.	.	5.590000	0.760000
Jansson et al. 1984	communities of marine phytoplankton	aquatic	1.706048	201.721000	.	.	0.393635	.	.
Jensen et al. 1990	communities of marine phytoplankton	aquatic	.	1157.728900	.	.	.	5.490000	0.810000
Johnson et al. 1981	communities of marine phytoplankton	aquatic	.	2315.457800	.	.	.	5.590000	0.760000
Johnson et al. 1981	communities of marine phytoplankton	aquatic	.	2723.374500	.	.	.	.	.
Johnson et al. 1981	communities of marine phytoplankton	aquatic	.	3671.250300	.	.	.	.	.
Joiris et al. 1982	communities of marine phytoplankton	aquatic	4.600000	516.380100	80.300000	15.550561	.	.	.
Kamiyama 1994	communities of marine phytoplankton	aquatic	.	1971.204400	295.650000	14.998445	.	.	.
Kemp and Boynton 1980	communities of marine phytoplankton	aquatic	.	690.864700	.	.	.	.	.
Kemp and Boynton 1980	communities of marine phytoplankton	aquatic	.	530.527500	.	.	.	.	.
Kenney et al. 1988	communities of marine phytoplankton	aquatic	.	162.695100	.	.	.	.	.
King et al. 1978	communities of marine phytoplankton	aquatic	.	75.452800	.	.	.	.	.

Knauer et al. 1979	communities of marine phytoplankton	aquatic	3.200000	73.000000	.	.	.	4.660000	0.592000
Kruse 1993	communities of marine phytoplankton	aquatic	.	400.843000	.	.	.	.	.
Kuparinen 1986	communities of marine phytoplankton	aquatic	.	141.474000	.	.	.	.	.
Lawes et al. 1988	communities of marine phytoplankton	aquatic	11.200000	742.738500	430.700000	57.988108	.	.	.
LeBorgne et al. 1989	communities of marine phytoplankton	aquatic	.	160.600000	158.990000	98.997509	.	4.870000	0.967700
LeBorgne et al. 1997	communities of marine phytoplankton	aquatic	.	164.615000	27.370000	16.626674	.	3.230000	0.326000
Lenz et al. 1993	communities of marine phytoplankton	aquatic	.	80.168600	80.168600	100.000000	.	.	.
Lenz et al. 1993	communities of marine phytoplankton	aquatic	.	238.147900	127.750000	53.643135	.	.	.
Lenz et al. 1993	communities of marine phytoplankton	aquatic	.	252.295300	127.750000	50.635109	.	.	.
Lenz et al. 1993	communities of marine phytoplankton	aquatic	.	120.252900	58.400000	48.564317	.	.	.
Lignell et al. 1993	communities of marine phytoplankton	aquatic	3.200000	288.350000	47.450000	16.455696	.	.	.
Lindeboom et al. 1989	communities of marine phytoplankton	aquatic	2.100000	549.390700	.	.	.	.	.
McLusky 1981	communities of marine phytoplankton	aquatic	.	63.663300	.	.	.	.	.
Mee 1977	communities of marine phytoplankton	aquatic	.	2494.658200	.	.	.	.	.
Menzel and Ryther 1960	communities of marine phytoplankton	aquatic	.	37.726400	.	.	.	.	.
Muller and Suess 1979	communities of marine phytoplankton	aquatic	7.500000	160.001400	.	.	.	.	.
Muller and Suess 1979	communities of marine phytoplankton	aquatic	0.860000	64.280150	.	.	.	.	.
Muller and Suess 1979	communities of marine phytoplankton	aquatic	3.000000	59.998700	.	.	.	.	.
Murray and Wetzel 1987	communities of marine phytoplankton	aquatic	.	188.632000	.	.	.	.	.
Murray and Wetzel 1987	communities of marine phytoplankton	aquatic	.	315.958600	.	.	.	.	.
Murray et al. 1994	communities of marine phytoplankton	aquatic	1.732000	430.700000	.		0.948600	.	.
Nedwell et al. 1993	communities of marine phytoplankton	aquatic	4.240000	188.632000	.	.	.	.	.
Newell et al. 1981	communities of marine phytoplankton	aquatic	.	448.950000	.	.	.	.	.
Nixon and Pilson 1984	communities of marine phytoplankton	aquatic	.	299.453300	.	.	.	.	.
Odum and Wilson 1962	communities of marine phytoplankton	aquatic	.	2565.395200	.	.	.	.	.
Packard 1979	communities of marine phytoplankton	aquatic	.	679.075200	.	.	.	.	.
Packard and Le B. Williams 1981	communities of marine phytoplankton	aquatic	0.630000	242.863700	.	.	.	.	.
Packard and Le B. Williams 1981	communities of marine phytoplankton	aquatic	0.780000	214.568900	.	.	.	.	.
Packard and le B. Williams 1981	communities of marine phytoplankton	aquatic	0.890000	221.642600	.	.	.	.	.
Perry 1976	communities of marine phytoplankton	aquatic	0.439560	43.800000	.	.	.	5.690000	0.790000
Pomeroy and Johannes 1968	communities of marine phytoplankton	aquatic	.	848.844000	.	.	.	5.590000	0.760000
Pomeroy and Johannes 1968	communities of marine phytoplankton	aquatic	.	1242.613300	.	.	.	5.490000	0.810000
Pomeroy and Johannes 1968	communities of marine phytoplankton	aquatic	.	4258.367400	.	.	.	.	.
Pomeroy and Johannes 1968	communities of marine phytoplankton	aquatic	.	509.306400	.	.	.	.	.
Pomeroy and Johannes 1968	communities of marine phytoplankton	aquatic	.	580.043400	.	.	.	.	.
Pomeroy and Johannes 1968	communities of marine phytoplankton	aquatic	.	315.958600	.	.	.	5.490000	0.810000
Pomeroy and Johannes 1968	communities of marine phytoplankton	aquatic	.	264.084800	.	.	.	.	.
Poumba 1994	communities of marine phytoplankton	aquatic	2.500000	374.906100	.	.	.	.	.
Prego 1993	communities of marine phytoplankton	aquatic	4.000000	127.326600	.	.	.	.	.
Randall and Day 1987	communities of marine phytoplankton	aquatic	.	370.190000	.	.	.	5.490000	0.810000
Randall and Day 1987	communities of marine phytoplankton	aquatic	.	233.432100	.	.	.	.	.
Schlesinger 1997	communities of marine phytoplankton	aquatic	.	235.790000	.	.	.	.	.
Shin et al. 1987	communities of marine phytoplankton	aquatic	.	.	.	.	.	.	0.512000
Skjoldal and Lännergren 1978	communities of marine phytoplankton	aquatic	2.990000	1619.877300	175.200000	10.815634	.	.	.
Small et al. 1989	communities of marine phytoplankton	aquatic	1.270000	250.338000	.		0.098267	.	.
Smith 1977	communities of marine phytoplankton	aquatic	.	455.074700	.	.	.	.	.
Smith 1977	communities of marine phytoplankton	aquatic	.	464.506300	.	.	.	.	.
Smith 1992	communities of marine phytoplankton	aquatic	1.000000	37.726400	.	.	.	.	.
Smith 1992	communities of marine phytoplankton	aquatic	1.000000	37.726400	.	.	.	.	.
Smith 1992	communities of marine phytoplankton	aquatic	1.000000	37.726400	.	.	.	.	.
Smith 1992	communities of marine phytoplankton	aquatic	1.000000	37.726400	.	.	.	.	.
Smith 1992	communities of marine phytoplankton	aquatic	1.000000	37.726400	.	.	.	.	.
Smith and Hollibaugh 1993	communities of marine phytoplankton	aquatic	.	115.537100	.	.	.	.	.
Smith et al. 1986	communities of marine phytoplankton	aquatic	0.660000	247.579500	.	.	.	.	.
Smith et al. 1986	communities of marine phytoplankton	aquatic	1.100000	221.642600	.	.	.	.	.
Smith et al. 1986	communities of marine phytoplankton	aquatic	0.280000	294.737500	.	.	.	.	.
Smith et al. 1986	communities of marine phytoplankton	aquatic	5.250000	858.275600	.	.	.	.	.
Sorokin 1983	communities of marine phytoplankton	aquatic	0.715400	179.200400	127.750000	71.288903	4.6	5.08	1.14
Strom and Welschemeyer 1991	communities of marine phytoplankton	aquatic	.	117.895000	51.100000	43.343653	.	.	.
Suess 1980	communities of marine phytoplankton	aquatic	.	401.500000	.	.	.	.	.
Suess 1980	communities of marine phytoplankton	aquatic	.	496.400000	.	.	.	.	.
Tijseen 1979	communities of marine phytoplankton	aquatic	.	181.558300	.	.	.	.	.
Tijssen and Eijgenraam 1982	communities of marine phytoplankton	aquatic	.	754.528000	.	.	.	.	.
Velimirov and Walenta-Simon 1992	communities of marine phytoplankton	aquatic	.	297.095400	.	.	.	.	.
Walsh 1988	communities of marine phytoplankton	aquatic	.	299.993500	100.010000	33.337389	.	.	.
Walsh 1988	communities of marine phytoplankton	aquatic	.	166.002000	35.999950	21.686456	.	.	.
Walsh 1988	communities of marine phytoplankton	aquatic	.	284.992000	87.016000	30.532787	.	.	.
Walsh 1988	communities of marine phytoplankton	aquatic	.	99.973500	22.998650	23.004746	.	.	.
Walsh 1988	communities of marine phytoplankton	aquatic	.	161.987000	67.999500	41.978369	.	.	.
Walsh et al. 1988	communities of marine phytoplankton	aquatic	.	135.000000	.	.	.	.	.
Welschemeyer and Lorenzen 1985	communities of marine phytoplankton	aquatic	1.947000	238.147900	182.500000	76.633050	.	.	.
Welschmeyer and Lorenzen 1985	communities of marine phytoplankton	aquatic	0.324000	28.294800	26.061000	92.105263	.	6.900000	0.980000

Zieman et al. 1993	communities of marine phytoplankton	aquatic	.	174.484600	65.700000	37.653753	.	.	.
Cattaneo 1983	freshwater benthic microalgal beds	aquatic	0.350000	0.876000	0.733650	83.750000	.	.	.
Flint and Goldman 1975	freshwater benthic microalgal beds	aquatic	0.239000	5.256000	1.752000	33.333333	.	.	.
Flint and Goldman 1975	freshwater benthic microalgal beds	aquatic	1.000000	25.440500	4.485850	17.632712	.	.	.
Hunter 1980	freshwater benthic microalgal beds	aquatic	.	2.839700	2.434550	85.732648	.	3.850000	.
Jacoby 1987	freshwater benthic microalgal beds	aquatic	0.875000	132.009550	102.200000	77.418641	.	.	.
Mason and Bryant 1975	freshwater benthic microalgal beds	aquatic	4.000000	7.811000	3.606200	46.168224	.	4.240000	0.405000
Naiman 1976	freshwater benthic microalgal beds	aquatic	35.970000	1109.600000	.	.	2.090000	.	.
Stanley 1976	freshwater benthic microalgal beds	aquatic	0.912500	7.570100	1.073100	14.175506	.	.	.
Admiraal et al. 1983	marine benthic microalgal beds	aquatic	1.114000	274.012800	40.150000	14.652600	.	.	.
Andersen and Kristensen 1988	marine benthic microalgal beds	aquatic	.	137.006400	.	.	.	.	.
Baird and Ulanowicz 1989	marine benthic microalgal beds	aquatic	0.177000	56.414400	17.885000	31.702899	0.1127	7.04	1.02
Baird and Ulanowicz 1993	marine benthic microalgal beds	aquatic	0.895000	34.923200	34.923200	100.000000	0.0814	5.3	0.81
Baird and Ulanowicz 1993	marine benthic microalgal beds	aquatic	.	80.592000	65.700000	81.521739	.	.	.
Baird and Ulanowicz 1993 (microphytobenthos average)	marine benthic microalgal beds	aquatic	.	.	.	.	.	4.050000	0.459000
Burns et al. 1993	marine benthic microalgal beds	aquatic	.	150.438400	.	.	.	.	.
Cadee and Hegeman 1977	marine benthic microalgal beds	aquatic	.	85.008500	.	.	.	.	.
Carpenter 1986	marine benthic microalgal beds	aquatic	20.000000	577.576000	138.700000	24.014156	.	.	.
Darley et al. 1981	marine benthic microalgal beds	aquatic	1.160000	72.532800	29.565000	40.760870	.	.	.
Davis and Lee 1983	marine benthic microalgal beds	aquatic	.	515.788800	350.400000	67.934783	.	.	.
Davis and McIntire 1983	marine benthic microalgal beds	aquatic	.	91.600400	.	.	.	.	.
Gould and Gallagher 1990	marine benthic microalgal beds	aquatic	17.610000	1058.441600	343.100000	32.415582	.	.	.
Hansen et al. 1992	marine benthic microalgal beds	aquatic	.	67.160000	.	.	.	.	.
Hansen et al. 1992	marine benthic microalgal beds	aquatic	.	42.982400	.	.	.	.	.
Klumpp et al. 1992	marine benthic microalgal beds	aquatic	.	80.592000	20.440000	25.362319	.	1.760000	.
Kristensen 1993	marine benthic microalgal beds	aquatic	1.208000	110.142400	.	.	.	.	.
Lindeboom and Sande 1989	marine benthic microalgal beds	aquatic	.	265.953600	.	.	.	.	.
Lindeboom and Sande 1989	marine benthic microalgal beds	aquatic	.	179.988800	.	.	.	.	.
Lindeboom and Sande 1989	marine benthic microalgal beds	aquatic	.	222.971200	.	.	.	.	.
Lindeboom and Sande 1989	marine benthic microalgal beds	aquatic	.	131.633600	.	.	.	.	.
McLahlan and Bate 1984	marine benthic microalgal beds	aquatic	1.260000	198.793600	58.400000	29.377203	.	.	.
Merino et al. 1992	marine benthic microalgal beds	aquatic	.	1249.176000	.	.	.	.	.
Montagna 1984	marine benthic microalgal beds	aquatic	.	3153.833600	237.250000	7.522591	.	.	.
Murray and Wetzel 1987	marine benthic microalgal beds	aquatic	.	77.905600	.	.	.	.	.
Murray and Wetzel 1987	marine benthic microalgal beds	aquatic	.	166.556800	.	.	.	.	.
Nicotri 1977	marine benthic microalgal beds	aquatic	0.047300	0.209539	0.127750	60.967113	.	.	.
Nicotri 1977	marine benthic microalgal beds	aquatic	0.108000	0.564144	0.438000	77.639752	.	.	.
Nicotri 1977	marine benthic microalgal beds	aquatic	0.320000	2.955040	1.642500	55.583004	.	.	.
Ostergard and Kristensen 1992	marine benthic microalgal beds	aquatic	.	110.142400	.	.	.	.	.
Panatmat 1988	marine benthic microalgal beds	aquatic	.	107.456000	.	.	.	.	.
Rizzo and Wetzel 1985	marine benthic microalgal beds	aquatic	.	231.030400	.	.	.	.	.
Rizzo and Wetzel 1985	marine benthic microalgal beds	aquatic	.	99.396800	.	.	.	.	.
Rizzo and Wetzel 1985	marine benthic microalgal beds	aquatic	.	112.828800	.	.	.	.	.
Vidal 1991	marine benthic microalgal beds	aquatic	.	88.651200	.	.	.	.	.
Anderson et al.1981	marine macroalgal beds	aquatic	481.264637	1500.150000	.	.	.	3.800000	.
Atkinson and Grigg 1984	marine macroalgal beds	aquatic	.	628.785500	.	.	.	.	.
Bach and Josselyn 1979	marine macroalgal beds	aquatic	.	1131.500000	.	.	.	.	.
Baird and Ulanowicz 1993	marine macroalgal beds	aquatic	318.340000	526.534400	167.900000	31.887755	15.9716	.	.
Baird and Ulanowicz 1993	marine macroalgal beds	aquatic	96.500000	513.102400	31.025000	6.046551	0.8823	.	.
Bak 1994	marine macroalgal beds	aquatic	.	435.813650	346.750000	79.563823	.	.	.
Bak 1994	marine macroalgal beds	aquatic	.	837.138450	452.600000	54.065131	.	.	.
Bak 1994	marine macroalgal beds	aquatic	.	1018.988750	540.200000	53.013343	.	.	.
Bak 1994	marine macroalgal beds	aquatic	.	683.506300	273.750000	40.050838	.	.	.
Bak 1994	marine macroalgal beds	aquatic	.	1078.560400	492.750000	45.685898	.	.	.
Birch et al. 1983	marine macroalgal beds	aquatic	.	216.007000	21.600700	10.000000	.	2.600000	0.235600
Brouwer 1996	marine macroalgal beds	aquatic	260.000000	854.100000	.	.	.	3.210000	0.530000
Bustamante et al. 1995	marine macroalgal beds	aquatic	125.320000	1326.5	.	.	81.6	2.42	.
Bythell et al. 1993	marine macroalgal beds	aquatic	.	467.167150	43.800000	9.375659	.	.	.
Bythell et al. 1993	marine macroalgal beds	aquatic	.	467.167150	28.470000	6.094178	.	.	.
Chapman and Craigie 1978	marine macroalgal beds	aquatic	316.153846	1500.150000	.	.	.	2.170000	.
Drew and Hastings 1992	marine macroalgal beds	aquatic	.	3.066000	0.328500	10.714286	.	2.000000	0.250000
Dunton and Schell 1986	marine macroalgal beds	aquatic	7.519084	7.190500	0.719050	10.000000	.	1.900000	0.250000
Egan and Yarish 1990	marine macroalgal beds	aquatic	418.367347	1496.500000	.	.	.	2.060000	.
Gathuso et al. 1993	marine macroalgal beds	aquatic	.	2409.000000	.	.	.	.	.
Gathuso et al. 1993	marine macroalgal beds	aquatic	.	1943.917000	.	.	.	.	.
Gathuso et al. 1993	marine macroalgal beds	aquatic	.	7493.486500	.	.	.	.	.
Gathuso et al. 1993	marine macroalgal beds	aquatic	.	2523.956750	.	.	.	.	.
Geertz-Hansen et al. 1993; Pedersen and Borum 1996	marine macroalgal beds	aquatic	.	.	.	.	.	3.160000	0.278000
Gerard 1976	marine macroalgal beds	aquatic	.	1299.546000	131.400000	10.111223	.	2.420000	0.310000
Hanisack 1979	marine macroalgal beds	aquatic	126.696833	306.600000	.	.	.	1.390000	.
Hanisack 1979	marine macroalgal beds	aquatic	127.675277	252.580000	.	.	.	2.270000	.
Hanisack 1979	marine macroalgal beds	aquatic	34.567901	71.540000	.	.	.	2.380000	.

Hatcher et al. 1992	marine macroalgal beds	aquatic	.	1000.176650	.	.	.	.	.
Hauxwell et al. 1998; Peckol and Rivers 1996	marine macroalgal beds	aquatic	.	486.670000	127.567500	26.212320	.	2.600000	0.235600
Josselyn and Mathieson 1980	marine macroalgal beds	aquatic	.	954.000000	.	.	.	.	.
Khailov and Burlakova 1969	marine macroalgal beds	aquatic	.	0.070628	0.018250	25.839793	.	.	.
Khailov and Burlakova 1969	marine macroalgal beds	aquatic	.	1.412550	0.511000	36.175711	.	.	.
Khailov and Burlakova 1969	marine macroalgal beds	aquatic	.	543.047000	102.200000	18.819734	.	.	.
Khailov and Burlakova 1969	marine macroalgal beds	aquatic	.	2.825100	0.547500	19.379845	.	.	.
Khailov and Burlakova 1969	marine macroalgal beds	aquatic	.	25.112000	8.395000	33.430233	.	.	.
Khailov and Burlakova 1969	marine macroalgal beds	aquatic	.	2.354250	0.766500	32.558140	.	.	.
Khailov and Burlakova 1969	marine macroalgal beds	aquatic	.	7.690550	2.117000	27.527290	.	.	0.250000
Khailov and Burlakova 1969	marine macroalgal beds	aquatic	.	13.968550	4.380000	31.356154	.	.	.
Khailov and Burlakova 1969	marine macroalgal beds	aquatic	.	7.062750	1.679000	23.772610	.	.	.
Khailov and Burlakova 1969	marine macroalgal beds	aquatic	.	20.403500	6.205000	30.411449	.	.	.
Kiene and Hutchings 1994	marine macroalgal beds	aquatic	.	495.385300	5.840000	1.178880	.	.	.
Kiene and Hutchings 1994	marine macroalgal beds	aquatic	.	495.385300	178.850000	36.103211	.	.	.
Kiene and Hutchings 1994	marine macroalgal beds	aquatic	.	495.385300	91.250000	18.420006	.	.	.
Kiene and Hutchings 1994	marine macroalgal beds	aquatic	.	495.385300	178.850000	36.103211	.	.	.
Kiene and Hutchings 1994	marine macroalgal beds	aquatic	.	495.385300	12.045000	2.431441	.	.	.
Kiene and Hutchings 1994	marine macroalgal beds	aquatic	.	495.385300	160.600000	32.419210	.	.	.
Klumpp and Polunin 1989	marine macroalgal beds	aquatic	.	693.719000	521.950000	75.239398	.	.	.
Klumpp et al. 1989	marine macroalgal beds	aquatic	17.348300	183.631500	127.750000	69.568674	1.733400	.	.
Klumpp et al. 1993	marine macroalgal beds	aquatic	.	32.959500	5.475000	16.611296	.	1.760000	.
Koop et al. 1982	marine macroalgal beds	aquatic	264.000000	1175.555500	105.850000	9.004254	.	2.420000	0.310000
Kristensen et al. 1992	marine macroalgal beds	aquatic	.	535.199500	.	.	.	.	.
Lapointe et al. 1981	marine macroalgal beds	aquatic	211.904762	1299.400000	.	.	.	3.950000	.
Mann 1972 (averages for whole bay)	marine macroalgal beds	aquatic	73.810000	659.190000	62.050000	9.413068	.	2.420000	0.310000
Mann 1972 (averages for seaweed zone)	marine macroalgal beds	aquatic	264.100000	1750.000000	.	.	12.950000	1.820000	0.270000
Mann 1985	marine macroalgal beds	aquatic	.	1123.762000	135.050000	12.017669	.	.	.
Mann 1985	marine macroalgal beds	aquatic	.	272.775450	.	.	.	.	.
Mann 1985	marine macroalgal beds	aquatic	.	3821.991650	.	.	.	.	.
Mann 1985	marine macroalgal beds	aquatic	.	1072.289700	.	.	.	.	.
Mann 1985	marine macroalgal beds	aquatic	.	2085.007750	.	.	.	.	.
Mann 1985	marine macroalgal beds	aquatic	.	3608.787850	.	.	.	.	.
Mann 1985	marine macroalgal beds	aquatic	.	1931.375600	.	.	.	.	.
Mann 1985	marine macroalgal beds	aquatic	.	1191.433000	.	.	.	.	.
Mann 1985	marine macroalgal beds	aquatic	.	6270.700000	.	.	.	.	.
Mann 1985	marine macroalgal beds	aquatic	.	2821.815000	.	.	.	.	.
Mann 1985	marine macroalgal beds	aquatic	.	1881.210000	.	.	.	.	.
Mann 1985	marine macroalgal beds	aquatic	.	4047.736850	.	.	.	.	.
Mann 1985	marine macroalgal beds	aquatic	.	5925.811500	.	.	.	.	.
Mann 1988	marine macroalgal beds	aquatic	.	916.588000	113.150000	12.344696	.	.	.
Mann 1988	marine macroalgal beds	aquatic	.	200.421500	.	.	.	.	.
Mann 1988	marine macroalgal beds	aquatic	.	200.421500	.	.	.	.	.
McLusky 1981	marine macroalgal beds	aquatic	.	92.600500	25.915000	27.985810	.	.	.
McQuaid and Branch 1985-exposed	marine macroalgal beds	aquatic	146.320000	1548.75486	.	.	20.76	2.29	0.25
McQuaid and Branch 1985-protected	marine macroalgal beds	aquatic	93.430000	988.93538	.	.	15.672	2.29	0.25
Nakamori et al. 1992	marine macroalgal beds	aquatic	.	1423.500000	.	.	.	.	.
Neudecker 1979	marine macroalgal beds	aquatic	.	316.670350	105.850000	33.425927	.	.	.
Newell et al. 1982	marine macroalgal beds	aquatic	454.600000	1124.200000	135.050000	12.012987	6.26	2.54	0.25
Pattersen et al. 1991	marine macroalgal beds	aquatic	.	874.762650	.	.	.	.	.
Polunin and Klumpp 1992	marine macroalgal beds	aquatic	26.400000	718.831000	386.900000	53.823500	.	2.500000	0.310000
Reichardt and Dieckmann 1985	marine macroalgal beds	aquatic	.	.	5.292500	.	.	4.300000	.
Sakshaug et al. 1994	marine macroalgal beds	aquatic	.	.	18.250000	.	.	1.100000	.
Sfriso and Marcomini 1997	marine macroalgal beds	aquatic	.	815.994000	530.418000	65.002684	.	1.980000	0.164500
Smith 1973	marine macroalgal beds	aquatic	.	3009.936000	.	.	.	.	.
Smith 1973	marine macroalgal beds	aquatic	.	1241.598600	.	.	.	.	.
Smith 1973	marine macroalgal beds	aquatic	.	627.070000	.	.	.	.	.
Smith and Jokiel 1978	marine macroalgal beds	aquatic	.	1883.400000	.	.	.	.	.
Smith and Pesret 1974	marine macroalgal beds	aquatic	.	949.000000	.	.	.	.	.
Smith et al. 1978	marine macroalgal beds	aquatic	.	1883.400000	.	.	.	.	.
Sorokin 1983	marine macroalgal beds	aquatic	.	7.533600	2.372500	31.492248	.	2.5	0.31
Spencer 1977	marine macroalgal beds	aquatic	.	1357.606550	.	.	.	.	.
Wallentinus 1981	marine macroalgal beds	aquatic	.	.	.	.	.	.	0.310000
Wanders 1977	marine macroalgal beds	aquatic	37.680000	811.431500	536.550000	66.123881	.	.	.
Williams and Carpenter1988	marine macroalgal beds	aquatic	.	733.650000	550.237500	75.000000	.	2.670000	.
Yap et al. 1994	marine macroalgal beds	aquatic	.	3386.178000	.	.	.	.	.
Anderson 1976	meadows of freshwater submerged macrophytes	aquatic	30.800000	46.720000	16.060000	34.375000	.	1.730000	.
Anderson 1976	meadows of freshwater submerged macrophytes	aquatic	.	1500.150000	.	.	.	3.800000	.
Bayley et al. 1985	meadows of freshwater submerged macrophytes	aquatic	.	327.600000	.	.	.	.	.
Boyd 1970	meadows of freshwater submerged macrophytes	aquatic	84.120983	162.425000	.	.	.	1.320000	0.170000
Cyr and Pace 1993	meadows of freshwater submerged macrophytes	aquatic	.	39.63466511	.	.	0.079733229	.	.

Cyr and Pace 1993	meadows of freshwater submerged macrophytes	aquatic	.	55.35910801	.	.	0.021274965	.	.
Cyr and Pace 1993	meadows of freshwater submerged macrophytes	aquatic	.	55.35910801	.	.	0.090994764	.	.
Cyr and Pace 1993	meadows of freshwater submerged macrophytes	aquatic	.	80.18187793	.	.	0.142593054	.	.
Cyr and Pace 1993	meadows of freshwater submerged macrophytes	aquatic	.	94.07592976	.	.	0.34102553	.	.
Cyr and Pace 1993	meadows of freshwater submerged macrophytes	aquatic	.	139.2610317	.	.	0.17156403	.	.
Cyr and Pace 1993	meadows of freshwater submerged macrophytes	aquatic	.	106.4406617	.	.	0.298820126	.	.
Flint and Goldman 1975	meadows of freshwater submerged macrophytes	aquatic	51.430000	201.115000	28.068500	13.956443	.	.	.
Jupp and Spence 1977	meadows of freshwater submerged macrophytes	aquatic	35.000000	111.325000	15.494250	13.918033	.	.	0.385000
Klopatek and Stearns 1978	meadows of freshwater submerged macrophytes	aquatic	50.000000	401.500000	52.560000	13.090909	.	.	.
Mason and Bryant 1975	meadows of freshwater submerged macrophytes	aquatic	146.508197	326.200500	.	.	.	2.600000	0.200000
Neeley and Davis 1985	meadows of freshwater submerged macrophytes	aquatic	.	369.600000	.	.	.	.	.
Neill 1992	meadows of freshwater submerged macrophytes	aquatic	128.000000	321.200000	.	.	.	1.760000	0.210000
Odum 1957	meadows of freshwater submerged macrophytes	aquatic	323.600000	1462.200000	316.820000	21.667351	14.8	2.22	0.27
Rogers and Breen 1982	meadows of freshwater submerged macrophytes	aquatic	.	93.650000	.	.	.	.	.
Schalles and Shure 1989	meadows of freshwater submerged macrophytes	aquatic	13.820000	24.272500	.	.	.	.	.
Sheldon 1987	meadows of freshwater submerged macrophytes	aquatic	32.800000	74.460000	8.650500	11.617647	.	.	.
Soszka 1975	meadows of freshwater submerged macrophytes	aquatic	37.800000	81.030000	5.292500	6.531532	.	.	.
Soszka 1975	meadows of freshwater submerged macrophytes	aquatic	19.250000	62.780000	24.345500	38.779070	.	.	.
Soszka 1975	meadows of freshwater submerged macrophytes	aquatic	76.200000	167.535000	15.220500	9.084967	.	.	.
Soszka 1975	meadows of freshwater submerged macrophytes	aquatic	19.750000	55.480000	16.023500	28.881579	.	.	.
Wallace and O'Hop 1985	meadows of freshwater submerged macrophytes	aquatic	13.000000	365.000000	62.050000	17.000000	.	4.020000	0.450000
Baird and Ulanowicz 1993	seagrass meadows	aquatic	162.100000	665.442450	.	.	9.4015	.	.
Baldwin and Lovvorn 1994	seagrass meadows	aquatic	.	7.865750	2.701000	34.338747	.	.	.
Cebrian and Duarte 1998 (CN)	seagrass meadows	aquatic	.	149.110000	12.670000	8.497083	.	2.380000	0.188700
Cebrian and Duarte 1998 (EA, CS, TH)	seagrass meadows	aquatic	.	484.790000	57.620000	11.885559	.	2.220000	0.288500
Cebrian and Duarte 1998 (TC)	seagrass meadows	aquatic	.	89.300000	5.270000	5.901456	.	2.120000	0.189000
Cebrian and Duarte 1998 (TT)	seagrass meadows	aquatic	.	570.520000	54.770000	9.600014	.	2.550000	0.189600
Cebrian and Duarte 1998 (ZM)	seagrass meadows	aquatic	.	335.800000	18.800000	5.598571	.	2.290000	0.199700
Cebrian and Duarte 1998 (ZN)	seagrass meadows	aquatic	.	126.870000	5.836000	4.599984	.	2.720000	0.279000
Cebrian et al. 1996	seagrass meadows	aquatic	.	188.2508727	8.2830384	4.400000	.	1.650000	0.112000
Cebrian et al. 1996	seagrass meadows	aquatic	.	402.961014	9.268103323	2.300000	.	2.590000	0.165000
Cebrian et al. 1996	seagrass meadows	aquatic	.	677.4919008	10.16237851	1.500000	.	2.150000	0.156000
Cebrian et al. 1996	seagrass meadows	aquatic	.	238.953863	3.584307945	1.500000	.	2.640000	0.119000
Cebrian et al. 1996	seagrass meadows	aquatic	.	951.903414	19.03806828	2.000000	.	1.810000	0.124000
Cebrian et al. 1996	seagrass meadows	aquatic	.	961.8975716	17.31415629	1.800000	.	1.860000	0.113000
Cebrian et al. 1996	seagrass meadows	aquatic	.	375.776658	4.885096554	1.300000	.	1.320000	0.103000
Cebrian et al. 1996	seagrass meadows	aquatic	.	458.8172563	7.799893357	1.700000	.	2.100000	0.107000
Cebrian et al. 1996	seagrass meadows	aquatic	.	167.8219703	1.510397733	0.900000	.	1.420000	0.115000
Cebrian et al. 1996	seagrass meadows	aquatic	.	919.9008	2.7597024	0.300000	.	2.020000	0.229000
Cebrian et al. 1996	seagrass meadows	aquatic	.	676.3322816	10.82131651	1.600000	.	1.330000	0.181000
Cebrian et al. 1996	seagrass meadows	aquatic	.	1075.268133	9.6774132	0.900000	.	1.970000	0.235000
Erftemeier et al. 1993	seagrass meadows	aquatic	53.800000	335.080950	.	.	.	.	.
Erftemeier et al. 1993	seagrass meadows	aquatic	47.800000	476.664450	.	.	.	.	.
Erftemeier et al. 1993	seagrass meadows	aquatic	22.830000	220.241000	.	.	.	.	.
Greenway 1976	seagrass meadows	aquatic	99.600000	876.244550	423.400000	48.319844	0.794328247	1.800000	0.140000
Harrison 1982	seagrass meadows	aquatic	.	360.000000	.	.	.	.	.
Hauxwell et al. 2002; Hauxwell et al. 1998	seagrass meadows	aquatic	32.000000	158	.	.	0.141	1.93	0.35
Hauxwell et al. 2002; Hauxwell et al. 1998	seagrass meadows	aquatic	12.800000	63.6	.	.	0.162	1.47	0.35
Hemminga and Nieuwenhuize 1991	seagrass meadows	aquatic	.	529.250000	.	.	.	.	.
Jacobs et al. 1981	seagrass meadows	aquatic	.	10.950000	0.511000	4.666667	.	.	0.202579
Kirkman and Reid 1979	seagrass meadows	aquatic	.	160.461300	3.431000	2.138210	.	.	.
Klumpp et al. 1989	seagrass meadows	aquatic	273.728100	1094.912400	76.650000	7.000560	0.569900	1.200000	0.120000
Klumpp et al. 1993	seagrass meadows	aquatic	.	582.065500	69.350000	11.914467	.	1.210000	.
Lindeboom and De Bree 1982	seagrass meadows	aquatic	.	493.969100	.	.	.	.	.
Nienhuis and Groenedijk 1986	seagrass meadows	aquatic	17.000000	34.609300	2.628000	7.593335	.	.	.
Ott and Maurer 1977	seagrass meadows	aquatic	461.600000	555.321950	51.100000	9.201869	.	.	.
Peduzzi and Herndl 1991	seagrass meadows	aquatic	.	250.000000	.	.	.	.	.
Pellikaan and Nienhuis 1988	seagrass meadows	aquatic	50.000000	162.034450	8.869500	5.473836	.	2.510000	0.416200
Pergent et al. 1994	seagrass meadows	aquatic	.	190.398600	.	.	.	.	.
Romero et al. 1992	seagrass meadows	aquatic	89.100000	201.480000	.	.	.	.	.
Tersching 1989	seagrass meadows	aquatic	.	489.249650	17.520000	3.580994	.	.	.
Wahbeh and Mahasneh 1985	seagrass meadows	aquatic	.	321.200000	.	.	.	.	.
Wetzel and Penhale 1983	seagrass meadows	aquatic	.	1054.010500	.	.	.	.	.
Wetzel and Penhale 1983	seagrass meadows	aquatic	.	602.516450	.	.	.	.	.
Zieman et al. 1979	seagrass meadows	aquatic	30.800000	393.287500	58.400000	14.849188	.	.	.
Zieman et al. 1979	seagrass meadows	aquatic	2.320000	47.194500	6.935000	14.694509	.	.	.
Billings 1987	tundra shrublands and grasslands	terrestrial	75.270000	72.000000	.	.	0.12	1.0975	0.09275
Bliss 1975	tundra shrublands and grasslands	terrestrial	57.200000	35.489700	.	.	0.017	0.88	0.139
Bliss 1975	tundra shrublands and grasslands	terrestrial	399.200000	54.394700	2.467400	4.536104	0.077	0.880000	0.092750
Chapin et al. 1980	tundra shrublands and grasslands	terrestrial	63.000000	47	.	.	0.09	1.447	0.1072
McNaughton et al. 1989	tundra shrublands and grasslands	terrestrial	.	58.199250	0.032000	0.054983	.	.	.
McNaughton et al. 1989	tundra shrublands and grasslands	terrestrial	.	21.099993	0.062999	0.298574	.	.	.

McNaughton et al. 1989	tundra shrublands and grasslands	terrestrial	.	21.099993	0.099999	0.473929	.	.	.
McNaughton et al. 1989	tundra shrublands and grasslands	terrestrial	.	20.75957802	.	.	0.006353639	.	.
McNaughton et al. 1989	tundra shrublands and grasslands	terrestrial	.	22.28739728	.	.	0.021076705	.	.
McNaughton et al. 1989	tundra shrublands and grasslands	terrestrial	.	20.75957802	.	.	0.052728614	.	.
McNaughton et al. 1989	tundra shrublands and grasslands	terrestrial	.	61.41043258	.	.	0.052728614	.	.
McNaughton et al. 1989	tundra shrublands and grasslands	terrestrial	.	20.6259903	.	.	0.17491478	.	.
Moore 1989	tundra shrublands and grasslands	terrestrial	176.800000	29.674500	.	.	.	.	.
Moore 1989	tundra shrublands and grasslands	terrestrial	120.200000	49.939300	.	.	.	.	.
Moore 1989	tundra shrublands and grasslands	terrestrial	171.600000	25.221500	.	.	.	.	.
Moore 1989	tundra shrublands and grasslands	terrestrial	145.800000	31.317000	.	.	.	.	.
Moore 1989	tundra shrublands and grasslands	terrestrial	235.200000	31.755000	.	.	.	.	.
Bach et al. 1986	freshwater and marine marshes	terrestrial	.	836.711400	459.900000	54.965189	.	.	.
Bach et al. 1986	freshwater and marine marshes	terrestrial	.	547.846750	302.950000	55.298311	.	.	.
Bach et al. 1986	freshwater and marine marshes	terrestrial	.	567.768450	313.900000	55.286623	.	.	.
Bernard and Solsky 1977	freshwater and marine marshes	terrestrial	347.708895	470.850000	.	.	.	1.600000	.
Buth 1987	freshwater and marine marshes	terrestrial	.	3733.326580	.	.	.	.	.
Buth 1987	freshwater and marine marshes	terrestrial	.	5574.091660	.	.	.	.	.
Buth 1987	freshwater and marine marshes	terrestrial	.	4090.921095	.	.	.	.	.
Cahoon and Stevenson 1986	freshwater and marine marshes	terrestrial	117.600000	482.105140	73.000000	15.141925	.	.	.
Cammen 1980	freshwater and marine marshes	terrestrial	182.800000	226.4	.	.	2.9	0.9	0.18
Craft et al. 1988	freshwater and marine marshes	terrestrial	.	.	.	.	.	0.780000	0.053800
de la Cruz 1973	freshwater and marine marshes	terrestrial	400.000000	419.998200	.	.	.	.	.
de la Cruz 1973	freshwater and marine marshes	terrestrial	400.000000	419.998200	.	.	.	.	.
Gallagher et al. 1980	freshwater and marine marshes	terrestrial	280.085197	959.950000	.	.	.	0.940000	0.150000
Graneli 1989	freshwater and marine marshes	terrestrial	165.000000	40.880000	.	.	.	.	.
Hopkinson et al. 1978	freshwater and marine marshes	terrestrial	323.200000	539.878070	.	.	.	.	.
Hopkinson et al. 1978	freshwater and marine marshes	terrestrial	301.600000	1267.020120	211.700000	16.708496	.	.	.
Hopkinson et al. 1978	freshwater and marine marshes	terrestrial	550.400000	2415.506125	.	.	.	.	.
Hopkinson et al. 1978	freshwater and marine marshes	terrestrial	396.000000	926.359050	.	.	.	.	.
Hopkinson et al. 1978	freshwater and marine marshes	terrestrial	259.200000	598.647085	.	.	.	.	.
Karagatzides and Hutchinson 1991	freshwater and marine marshes	terrestrial	111.800000	103.295000	.	.	.	.	.
Karagatzides and Hutchinson 1991	freshwater and marine marshes	terrestrial	138.000000	161.330000	.	.	.	.	.
Lee 1990	freshwater and marine marshes	terrestrial	623.920000	879.543055	8.760000	0.995972	.	1.280000	.
Parsons and De la Cruz 1980	freshwater and marine marshes	terrestrial	242.500000	832.110000	2.964700	0.356287	0.089000	0.780000	0.140000
Patriquin and McClung 1978	freshwater and marine marshes	terrestrial	66.768293	159.870000	.	.	.	2.200000	.
Patriquin and McClung 1978	freshwater and marine marshes	terrestrial	119.883041	299.300000	.	.	.	2.030000	.
Reader and Stewart 1972	freshwater and marine marshes	terrestrial	.	192.600000	43.880000	22.782970	.	1.590000	0.114000
Reader and Stewart 1972	freshwater and marine marshes	terrestrial	.	447.400000	36.920000	8.252123	.	2.250000	0.175000
Richardson et al. 1999	freshwater and marine marshes	terrestrial	.	1196.400000	0.760000	0.063524	.	0.723400	0.013500
Smalley 1960	freshwater and marine marshes	terrestrial	.	503.006500	2.867221	0.570017	.	.	.
Squiers and Good 1974	freshwater and marine marshes	terrestrial	266.592428	436.905000	.	.	.	0.990000	.
Teal 1962	freshwater and marine marshes	terrestrial	250.000000	992.100660	51.100000	5.150687	0.281000	0.900000	0.180000
Udell et al 1969	freshwater and marine marshes	terrestrial	107.664234	215.350000	15.074500	7.000000	.	2.140000	.
White et al. 1978	freshwater and marine marshes	terrestrial	671.600000	517.964200	.	.	.	.	.
White et al. 1978	freshwater and marine marshes	terrestrial	589.200000	1387.546405	229.950000	16.572419	.	.	.
Boring et al. 1981	temperate and tropical shrublands and forests	terrestrial	.	350.200000	14.600000	4.169046	.	0.338900	0.061900
Bray 1964	temperate and tropical shrublands and forests	terrestrial	60.000000	132.130000	9.198000	6.961326	.	.	.
Chew and Chew 1970	temperate and tropical shrublands and forests	terrestrial	184.000000	52.560000	1.040250	1.979167	0.063641054	0.639600	.
Day 1982	temperate and tropical shrublands and forests	terrestrial	232.273680	233.503215	.	.	.	.	.
Day 1982	temperate and tropical shrublands and forests	terrestrial	446.683592	252.518304	.	.	.	.	.
Day 1982	temperate and tropical shrublands and forests	terrestrial	238.231947	239.493022	.	.	.	.	.
Day 1982	temperate and tropical shrublands and forests	terrestrial	208.449088	209.552509	.	.	.	.	.
De Angelis et al. 1981	temperate and tropical shrublands and forests	terrestrial	.	706.599850	15.000040	2.122848	.	.	.
De Angelis et al. 1981	temperate and tropical shrublands and forests	terrestrial	.	599.600100	2.200000	0.366911	.	.	.
De Angelis et al. 1981	temperate and tropical shrublands and forests	terrestrial	.	449.132500	7.520095	1.674360	.	.	.
De Angelis et al. 1981	temperate and tropical shrublands and forests	terrestrial	.	402.441700	36.399990	9.044786	.	.	.
De Angelis et al. 1981	temperate and tropical shrublands and forests	terrestrial	.	516.000500	16.000140	3.100799	.	.	.
De Angelis et al. 1981	temperate and tropical shrublands and forests	terrestrial	.	382.812000	8.400110	2.194317	.	.	.
De Angelis et al. 1981	temperate and tropical shrublands and forests	terrestrial	.	489.757000	6.840100	1.396631	.	.	.
De Angelis et al. 1981	temperate and tropical shrublands and forests	terrestrial	.	403.120600	7.120055	1.766234	.	.	.
Frangi and Lugo 1985	temperate and tropical shrublands and forests	terrestrial	9999.000000	876.000000	1.113250	0.127083	1.310000	.	0.033400
Gosz et al.1978	temperate and tropical shrublands and forests	terrestrial	7156.310000	468.952000	31.426500	6.701432	.	0.320000	0.061900
Gray 1982	temperate and tropical shrublands and forests	terrestrial	2592.890000	423.400000	.	.	.	0.640000	0.044000
Gray 1982	temperate and tropical shrublands and forests	terrestrial	370.000000	141.985000	.	.	.	0.530000	0.110000
Grier and Logan 1977	temperate and tropical shrublands and forests	terrestrial	26862.800000	332.000000	1.598700	0.481536	0.446	0.073300	0.011870
Grier and Logan 1977	temperate and tropical shrublands and forests	terrestrial	32528.800000	318.400000	2.398050	0.753156	0.668	0.073400	0.011880
Grier and Logan 1977	temperate and tropical shrublands and forests	terrestrial	20017.600000	251.200000	2.398050	0.954638	0.668	0.073300	0.011860
Grier and Logan 1977	temperate and tropical shrublands and forests	terrestrial	22590.400000	303.600000	2.398050	0.789872	0.668	0.073300	0.011870
Grier and Logan 1977	temperate and tropical shrublands and forests	terrestrial	39300.400000	402.800000	2.398050	0.595345	0.668	0.070800	0.011300
Grodzinski et al. 1966	temperate and tropical shrublands and forests	terrestrial	.	98.980700	1.376543	1.390718	.	.	.
Gruner, age 300	temperate and tropical shrublands and forests	terrestrial	7104	421.2	.	.	0.253528	.	.
Gruner, age 5000	temperate and tropical shrublands and forests	terrestrial	10644	388.208	.	.	1.477688	.	.

Gruner, age 150000	temperate and tropical shrublands and forests	terrestrial	5784	363.404	.	.	2.398276	.	.
Gruner, age 1400000	temperate and tropical shrublands and forests	terrestrial	5500	558	.	.	0.837252	.	.
Gruner, age 4100000	temperate and tropical shrublands and forests	terrestrial	8232	298.516	.	.	0.210936	.	.
Harris et al. 1975	temperate and tropical shrublands and forests	terrestrial	8011.000000	451.100000	4.489500	0.995234	0.101	0.3389	0.0619
Haukioja and Koponen 1975	temperate and tropical shrublands and forests	terrestrial	.	67.999865	3.559991	5.235291	0.042672096	.	.
Kira 1978	temperate and tropical shrublands and forests	terrestrial	17400.000000	846.800000	.	.	.	.	.
Leigh and Windsor 1983	temperate and tropical shrublands and forests	terrestrial	243.800000	280.320000	36.500000	13.020833	.	.	.
Lowman 1988	temperate and tropical shrublands and forests	terrestrial	456.400000	228.490000	50.005000	21.884984	.	.	.
Lowman 1988	temperate and tropical shrublands and forests	terrestrial	387.600000	193.815000	52.925000	27.306968	.	.	.
Lowman 1988	temperate and tropical shrublands and forests	terrestrial	490.400000	245.280000	35.186000	14.345238	.	.	.
McInnes et al. 1992	temperate and tropical shrublands and forests	terrestrial	.	490.001550	228.000900	46.530649	.	1.390000	.
McInnes et al. 1992	temperate and tropical shrublands and forests	terrestrial	.	228.000900	63.999100	28.069670	.	1.210000	.
McInnes et al. 1992	temperate and tropical shrublands and forests	terrestrial	.	353.998900	152.000600	42.938156	.	1.260000	.
McInnes et al. 1992	temperate and tropical shrublands and forests	terrestrial	.	301.999905	60.000160	19.867609	.	1.370000	.
McNaughton et al. 1989	temperate and tropical shrublands and forests	terrestrial	.	13.21169908	.	.	0.013483001	.	.
McNaughton et al. 1989	temperate and tropical shrublands and forests	terrestrial	.	41.15399548	.	.	0.015895208	.	.
McNaughton et al. 1989	temperate and tropical shrublands and forests	terrestrial	.	56.46689392	.	.	0.022091513	.	.
McNaughton et al. 1989	temperate and tropical shrublands and forests	terrestrial	.	75.99143688	.	.	0.254805335	.	.
McNaughton et al. 1989	temperate and tropical shrublands and forests	terrestrial	.	269.3346511	.	.	0.026663458	.	.
McNaughton et al. 1989	temperate and tropical shrublands and forests	terrestrial	.	367.1727248	.	.	0.148370268	.	.
McNaughton et al. 1989	temperate and tropical shrublands and forests	terrestrial	1810.040000	615.414014	.	.	0.865366424	.	.
McNaughton et al. 1989	temperate and tropical shrublands and forests	terrestrial	.	423.206256	.	.	0.668151443	.	.
McNaughton et al. 1989	temperate and tropical shrublands and forests	terrestrial	.	326.8914747	.	.	1.260613775	.	.
McNaughton et al. 1989	temperate and tropical shrublands and forests	terrestrial	.	210.7420355	.	.	2.164929131	.	.
McNaughton et al. 1989	temperate and tropical shrublands and forests	terrestrial	.	232.1704014	.	.	1.290605239	.	.
McNaughton et al. 1989	temperate and tropical shrublands and forests	terrestrial	.	250.8715411	.	.	0.427423874	.	.
Menhinick 1967	temperate and tropical shrublands and forests	terrestrial	.	221.920000	2.029400	0.914474	0.515881294	.	.
Nielsen 1978	temperate and tropical shrublands and forests	terrestrial	100.400000	114.610000	3.478450	3.035032	.	.	.
Ohmart et al. 1983	temperate and tropical shrublands and forests	terrestrial	194.000000	74.095000	1.241000	1.674877	.	0.530000	0.034000
Ohmart et al. 1983	temperate and tropical shrublands and forests	terrestrial	145.200000	65.335000	1.281150	1.960894	.	0.570000	0.025100
Ohmart et al. 1983	temperate and tropical shrublands and forests	terrestrial	200.000000	89.060000	2.120650	2.381148	.	0.580000	0.053700
Proctor et al. 1983	temperate and tropical shrublands and forests	terrestrial	1520.000000	332.880000	40.880000	12.280702	.	1.170000	.
Proctor et al. 1983	temperate and tropical shrublands and forests	terrestrial	1880.000000	264.260000	40.880000	15.469613	.	0.567000	.
Proctor et al. 1983	temperate and tropical shrublands and forests	terrestrial	1000.000000	318.280000	54.020000	16.972477	.	0.900000	.
Proctor et al. 1983	temperate and tropical shrublands and forests	terrestrial	2600.000000	246.740000	30.660000	12.426036	.	0.950000	.
Reichle and Crossley 1967	temperate and tropical shrublands and forests	terrestrial	40.000000	89.790000	7.993500	8.902439	.	.	.
Reichle et al. 1973	temperate and tropical shrublands and forests	terrestrial	.	327.868000	3.600000	1.098003	.	0.338900	0.061900
Schowalter et al. 1981	temperate and tropical shrublands and forests	terrestrial	.	127.998200	8.000070	6.250143	0.166879795	.	.
Soholt 1973	temperate and tropical shrublands and forests	terrestrial	.	13.542960	0.934473	6.900065	.	.	.
Svensson and Roswall 1980	temperate and tropical shrublands and forests	terrestrial	196.800000	56.940000	0.361350	0.634615	.	.	.
Tanner 1980	temperate and tropical shrublands and forests	terrestrial	315.294118	195.640000	.	.	.	1.050000	0.050000
Van Cleve et al. 1981	temperate and tropical shrublands and forests	terrestrial	927.152318	51.100000	.	.	.	0.390000	0.083300
Van Cleve et al. 1981	temperate and tropical shrublands and forests	terrestrial	597.727273	28.798500	.	.	.	0.210000	0.033000
Van Cleve et al. 1983	temperate and tropical shrublands and forests	terrestrial	4478.856462	188.000000	.	.	.	0.250000	0.025600
Van Cleve et al. 1983	temperate and tropical shrublands and forests	terrestrial	4422.700587	226.000000	.	.	.	0.240000	0.024500
Van Cleve et al. 1983	temperate and tropical shrublands and forests	terrestrial	6975.580703	146.400000	.	.	.	0.140000	0.017700
Van Cleve et al. 1983	temperate and tropical shrublands and forests	terrestrial	2036.770007	45.200000	.	.	.	0.240000	0.036000
Van Cleve et al. 1983	temperate and tropical shrublands and forests	terrestrial	4839.452055	220.800000	.	.	.	0.210000	0.026800
Varley 1970	temperate and tropical shrublands and forests	terrestrial	.	496	.	.	0.73	.	.
Whittaker 1975	temperate and tropical shrublands and forests	terrestrial	164.000000	157.680000	12.665500	8.032407	.	.	.
Whittaker and Woodwell 1969	temperate and tropical shrublands and forests	terrestrial	.	342.800700	9.719950	2.835452	.	.	.
Aerts 1989	temperate and tropical grasslands	terrestrial	80.000000	216.080000	.	.	.	0.955000	0.035000
Aerts et al. 1992	temperate and tropical grasslands	terrestrial	40.645161	45.990000	.	.	.	0.700000	0.125000
Aerts et al. 1992	temperate and tropical grasslands	terrestrial	38.194444	40.150000	.	.	.	1.400000	0.050000
Auclair et al. 1976	temperate and tropical grasslands	terrestrial	322.800000	327.999950	.	.	.	.	.
Boyer and Wheeler 1989	temperate and tropical grasslands	terrestrial	64.770932	149.650000	.	.	.	1.200000	0.700000
Boyer and Wheeler 1989	temperate and tropical grasslands	terrestrial	150.931677	88.695000	.	.	.	0.940000	0.400000
Collins and Steinauer 1998, unpubl. Data	temperate and tropical grasslands	terrestrial	91.458	182.916	.	.	1.32	.	.
Coppock et al. 1983	temperate and tropical grasslands	terrestrial	32.000000	76.650000	36.500000	47.619048	.	2.230000	.
Damman 1978	temperate and tropical grasslands	terrestrial	52.009132	41.573500	.	.	.	0.980000	0.084000
Damman 1978	temperate and tropical grasslands	terrestrial	38.059701	37.230000	.	.	.	0.410000	0.035000
Day and Detling 1990	temperate and tropical grasslands	terrestrial	.	56.246500	7.318250	13.011032	.	1.080000	.
Edroma 1984	temperate and tropical grasslands	terrestrial	.	190.000750	46.000950	24.210931	.	.	.
Frank and McNaughton 1992	temperate and tropical grasslands	terrestrial	.	120.997500	54.458000	45.007541	.	2.100000	.
Gandar 1982	temperate and tropical grasslands	terrestrial	8.650000	30.660000	12.994000	42.380952	.	.	.
Gandar 1982	temperate and tropical grasslands	terrestrial	6.400000	17.520000	4.336200	24.750000	.	.	.
Lock 1972	temperate and tropical grasslands	terrestrial	.	200.921550	56.921750	28.330336	.	.	.
McNaughton 1985	temperate and tropical grasslands	terrestrial	33.470000	265.720000	175.200000	65.934066	0.733	1.470000	0.373000
McNaughton 1985	temperate and tropical grasslands	terrestrial	40.000000	328.000000	248.000000	75.609756	0.849	2.520000	0.444000
McNaughton et al. 1989	temperate and tropical grasslands	terrestrial	.	62.61138244	.	.	0.023706072	.	.
McNaughton et al. 1989	temperate and tropical grasslands	terrestrial	.	43.89844399	.	.	0.106754852	.	.
McNaughton et al. 1989	temperate and tropical grasslands	terrestrial	.	172.5186675	.	.	0.120072762	.	.

McNaughton et al. 1989	temperate and tropical grasslands	terrestrial	.	103.5959644	.	.	0.17491478	.	.
McNaughton et al. 1989	temperate and tropical grasslands	terrestrial	.	105.6218997	.	.	0.226543517	.	.
McNaughton et al. 1989	temperate and tropical grasslands	terrestrial	.	136.7421673	.	.	0.580238655	.	.
McNaughton et al. 1989	temperate and tropical grasslands	terrestrial	427.590091	611.4538298	.	.	3.989692704	.	.
McNaughton et al. 1989	temperate and tropical grasslands	terrestrial	363.860431	520.3204158	.	.	3.989692704	.	.
McNaughton et al. 1989	temperate and tropical grasslands	terrestrial	.	324.7879307	.	.	0.540720122	.	.
McNaughton et al. 1989	temperate and tropical grasslands	terrestrial	.	306.4548318	.	.	0.608176183	.	.
McNaughton et al. 1989	temperate and tropical grasslands	terrestrial	.	469.2577704	.	.	1.451611169	.	.
McNaughton et al. 1989	temperate and tropical grasslands	terrestrial	.	428.7059422	.	.	1.35274574	.	.
McNaughton et al. 1989	temperate and tropical grasslands	terrestrial	.	396.7481933	.	.	1.174756677	.	.
McNaughton et al. 1989	temperate and tropical grasslands	terrestrial	.	371.9442393	.	.	1.321310236	.	.
McNaughton et al. 1989	temperate and tropical grasslands	terrestrial	.	287.2958496	.	.	1.020186728	.	.
McNaughton et al. 1989	temperate and tropical grasslands	terrestrial	.	255.7776153	.	.	0.825614459	.	.
McNaughton et al. 1989	temperate and tropical grasslands	terrestrial	.	196.2954971	.	.	0.825614459	.	.
McNaughton et al. 1989	temperate and tropical grasslands	terrestrial	.	250.8715411	.	.	1.451611169	.	.
McNaughton et al. 1989	temperate and tropical grasslands	terrestrial	.	212.106942	.	.	1.120792384	.	.
McNaughton et al. 1989	temperate and tropical grasslands	terrestrial	.	212.106942	.	.	0.354134121	.	.
Pakarinen 1978	temperate and tropical grasslands	terrestrial	178.531073	115.340000	.	.	.	0.830000	.
Pakarinen 1978	temperate and tropical grasslands	terrestrial	132.911392	76.650000	.	.	.	0.700000	0.035000
Ritchie, 1989-1999	temperate and tropical grasslands	terrestrial	17.74	35.48	.	.	0.0996	.	.
Ritchie et al. 1998	temperate and tropical grasslands	terrestrial	.	99.999000	45.000850	45.001300	.	1.450000	.
Scott et al. 1979	temperate and tropical grasslands	terrestrial	85.030000	121.600000	9.320000	7.664474	0.216136885	1.625000	0.180000
Sinclair 1975	temperate and tropical grasslands	terrestrial	116.560000	239.075000	65.700000	27.480916	0.337866414	1.440000	0.220000
Strugnell and Pigott 1978	temperate and tropical grasslands	terrestrial	98.520000	306.600000	164.250000	53.571429	.	1.600000	.
Thornton 1971	temperate and tropical grasslands	terrestrial	.	190.000750	46.000950	24.210931	.	.	.
Van Hook 1971	temperate and tropical grasslands	terrestrial	56.880000	127.385000	12.300500	9.656160	0.267073789	.	.
Whealer and Shaw 1991	temperate and tropical grasslands	terrestrial	56.000000	87.965000	.	.	.	.	.
Whealer and Shaw 1991	temperate and tropical grasslands	terrestrial	132.000000	140.160000	.	.	.	.	.
Whealer and Shaw 1991	temperate and tropical grasslands	terrestrial	240.000000	500.050000	.	.	.	.	.
Whealer and Shaw 1991	temperate and tropical grasslands	terrestrial	134.000000	247.835000	.	.	.	.	.
Whealer and Shaw 1991	temperate and tropical grasslands	terrestrial	178.000000	368.650000	.	.	.	.	.
Whealer and Shaw 1991	temperate and tropical grasslands	terrestrial	390.000000	700.800000	.	.	.	.	.
Whealer and Shaw 1991	temperate and tropical grasslands	terrestrial	90.000000	100.010000	.	.	.	.	.
Whealer and Shaw 1991	temperate and tropical grasslands	terrestrial	96.000000	100.010000	.	.	.	.	.
Whealer and Shaw 1991	temperate and tropical grasslands	terrestrial	276.000000	540.200000	.	.	.	.	.
Whealer and Shaw 1991	temperate and tropical grasslands	terrestrial	310.000000	598.600000	.	.	.	.	.
Whealer and Shaw 1991	temperate and tropical grasslands	terrestrial	228.000000	267.910000	.	.	.	.	.
Whealer and Shaw 1991	temperate and tropical grasslands	terrestrial	200.000000	278.130000	.	.	.	.	.
Wiegert and Evans 1964	temperate and tropical grasslands	terrestrial	424.100000	594.950000	8.176000	1.374233	.	.	.
Wiegert and Evans 1964	temperate and tropical grasslands	terrestrial	296.200000	199.509000	2.555000	1.280644	.	.	.
Brown et al. 1984	mangroves	terrestrial	3073.193916	118.004500	.	.	.	.	.
Christensen 1978	mangroves	terrestrial	.	1080.400000	16.060000	1.486486	.	.	.
Emmerson and McGwynne 1992	mangroves	terrestrial	.	525.600000	29.200000	5.555556	.	.	.
Flores-Verdugo et al. 1987	mangroves	terrestrial	.	463.550000	.	.	.	.	.
Golley et al. 1962	mangroves	terrestrial	1842.200000	1438.892050	36.500000	2.536674	1.000000	.	.
Johnstone 1981	mangroves	terrestrial	.	1533.000000	33.580000	2.190476	.	.	.
Lee 1989	mangroves	terrestrial	.	876.000000	47.450000	5.416667	.	.	.
Li and Lee 1998	mangroves	terrestrial	.	700.800000	26.207000	3.739583	.	.	.
Li and Lee 1998	mangroves	terrestrial	.	868.700000	27.995500	3.222689	.	.	.
Nessel 1978	mangroves	terrestrial	11370.967742	514.650000	.	.	.	.	0.013900
Odum 1971	mangroves	terrestrial	.	900.017000	23.039530	2.559899	.	.	.
Omuf et al. 1977	mangroves	terrestrial	.	1694.151150	105.850000	6.247967	.	.	.
Ong 1993	mangroves	terrestrial	.	900.017000	.	.	.	.	.
Ong et al. 1982	mangroves	terrestrial	.	.	.	.	.	0.655000	.
Robertson and Daniel 1989	mangroves	terrestrial	.	599.987000	15.800120	2.633410	.	.	.
Schlesinger 1978	mangroves	terrestrial	12285.251216	276.670000	.	.	.	0.330000	0.014900
Twilley et al. 1986	mangroves	terrestrial	6800.000000	199.311900	.	.	.	.	.
Twilley et al. 1986	mangroves	terrestrial	6544.000000	118.887800	.	.	.	.	.
Twilley et al. 1986	mangroves	terrestrial	4460.000000	181.828400	.	.	.	.	.
Van der Walk and Attiwill 1984	mangroves	terrestrial	.	104.901000	.	.	.	.	.
Wolff et al. 2000	mangroves	terrestrial	.	507.000000	13.500000	2.662722	.	0.981875	0.060650

Supporting Information: Data Set for Figure 2 in Main Text

system	ref	herbivore ID	herbivore ID2	common name	group	Nh	N max	N min	N SD	Ph	P max	P min	P SD	study mean he	study mean he	N source	P source	notes
1	Baird and Ulanowicz 1989	.	.	oysters	invert	9.39				0.084						Hatcher	Hatcher	up to trophic position 2.5, used abalone
1	Baird and Ulanowicz 1989	Mya	arenaria	soft-shelled cla	invert	10.7				0.084						Carmichael	Hatcher	up to trophic position 2.5, used abalone
2	Baird and Ulanowicz 1989	.	.	zooplankton	zoop	7.73213148				0.82336711								up to trophic position 2.5, used average copepod
53	Baird and Ular Kromme	zooplankton		zooplankton	invert	7.73213148				0.82336711								
54	Baird and Ular Ythan	zooplankton		zooplankton	invert	7.73213148				0.82336711								
55	Baird and Ular Ems	zooplankton		zooplankton	invert	7.73213148				0.82336711								
56	Baird and Ular Swartkops	zooplankton		zooplankton	invert	7.73213148				0.82336711								
57	Baird and Ular Ems	benthos		benthos	invert	10.045				1.01								
58	Baird and Ular Ythan	benthos		benthos	invert	10.045				1.01								
59	Baird and Ular Swartkops	benthos		benthos	invert	10.045				1.01								
60	Baird and Ular Kromme	benthos		benthos	invert	10.045				1.01								
3	Billings 1987	.	.	lemmings	mammal	11.6				2.76						Hayssen91 d.	Hayssen91 d. microps	
61	Bliss 1975	.	.	snow geese	bird													
61	Bliss 1975	.	.	arctic hare	mammal	11.3				3.43						Dierenfeld07 r	Gillooly&Allen05 rabbit	
61	Bliss 1975	.	.	collared lemm	mammal	11.6				2.76						Hayssen91 d.	Hayssen91 d. microps	
61	Bliss 1975	.	.	muskox	mammal	9.6				2.88						Dierenfeld07 b	Gillooly&Allen05 bull	
62	Bliss 1975	.	.	snow geese	bird													
62	Bliss 1975	.	.	arctic hare	mammal	11.3				3.43						Dierenfeld07 r	Gillooly&Allen05 rabbit	
62	Bliss 1975	.	.	collared lemm	mammal	11.6				2.76						Hayssen91 d.	Hayssen91 d. microps	
62	Bliss 1975	.	.	muskox	mammal	9.6				2.88						Dierenfeld07 b	Gillooly&Allen05 bull	
4	Brown et al 1991	Clausocalanus	furcatus	copepod	mar zoop	7.73213148	11.1111111	4.02298851	1.93306673	0.82336711	1.64021164	0.29693487	0.29184236	7.69707304	0.85634546			S Afr J Mar Sci 11: 537
4	Brown et al 1991	Corycella	concinna	protozoan	mar zoop	6.99943452	8.61230329	5.49019608	0.80682275	1.29091027	2.58333333	0.4434907	0.62454483	7.69707304	0.85634546			S Afr J Mar Sci 11: 537
4	Brown et al 1991	Temora	discaudata	copepod	mar zoop	7.73213148	11.1111111	4.02298851	1.93306673	0.82336711	1.64021164	0.29693487	0.29184236	7.69707304	0.85634546			S Afr J Mar Sci 11: 537
4	Brown et al 1991	Calanus	finmarchicus	copepod	mar zoop	7.73213148	11.1111111	4.02298851	1.93306673	0.82336711	1.64021164	0.29693487	0.29184236	7.69707304	0.85634546			S Afr J Mar Sci 11: 537
4	Brown et al 1991	Euterpina	acutifrons	copepod	mar zoop	7.73213148	11.1111111	4.02298851	1.93306673	0.82336711	1.64021164	0.29693487	0.29184236	7.69707304	0.85634546			S Afr J Mar Sci 11: 537
4	Brown et al 1991	Temora	turbinata	copepod	mar zoop	7.73213148	11.1111111	4.02298851	1.93306673	0.82336711	1.64021164	0.29693487	0.29184236	7.69707304	0.85634546			S Afr J Mar Sci 11: 537
4	Brown et al 1991	Pseudodiapton	nudus	copepod	mar zoop	7.73213148	11.1111111	4.02298851	1.93306673	0.82336711	1.64021164	0.29693487	0.29184236	7.69707304	0.85634546			S Afr J Mar Sci 11: 537
4	Brown et al 1991	Calocalanus	tenuis	copepod	mar zoop	7.73213148	11.1111111	4.02298851	1.93306673	0.82336711	1.64021164	0.29693487	0.29184236	7.69707304	0.85634546			S Afr J Mar Sci 11: 537
4	Brown et al 1991	Centropages	brachiatus	copepod	mar zoop	7.90389332	9.1503268	6.76328502	0.85719415	0.88347763	1.17424242	0.73809524	0.2518097	7.69707304	0.85634546			S Afr J Mar Sci 11: 537
4	Brown et al 1991	Calanoides	carinatus	copepod	mar zoop	7.73213148	11.1111111	4.02298851	1.93306673	0.82336711	1.64021164	0.29693487	0.29184236	7.69707304	0.85634546			S Afr J Mar Sci 11: 537
4	Brown et al 1991	Metridia	lucens	copepod	mar zoop	7.73213148	11.1111111	4.02298851	1.93306673	0.82336711	1.64021164	0.29693487	0.29184236	7.69707304	0.85634546			S Afr J Mar Sci 11: 537
4	Brown et al 1991	Nannocalanus	minor	copepod	mar zoop	7.73213148	11.1111111	4.02298851	1.93306673	0.82336711	1.64021164	0.29693487	0.29184236	7.69707304	0.85634546			S Afr J Mar Sci 11: 537
4	Brown et al 1991	Clausocalanus	arcuicornis	copepod	mar zoop	7.73213148	11.1111111	4.02298851	1.93306673	0.82336711	1.64021164	0.29693487	0.29184236	7.69707304	0.85634546			S Afr J Mar Sci 11: 537
4	Brown et al 1991	Paracalanus	parvus	copepod	mar zoop	7.73213148	11.1111111	4.02298851	1.93306673	0.82336711	1.64021164	0.29693487	0.29184236	7.69707304	0.85634546			S Afr J Mar Sci 11: 537
4	Brown et al 1991	Paracalanus	crassirostris	copepod	mar zoop	7.73213148	11.1111111	4.02298851	1.93306673	0.82336711	1.64021164	0.29693487	0.29184236	7.69707304	0.85634546			S Afr J Mar Sci 11: 537
4	Brown et al 1991	Ctenocalanus	vanus	copepod	mar zoop	7.73213148	11.1111111	4.02298851	1.93306673	0.82336711	1.64021164	0.29693487	0.29184236	7.69707304	0.85634546			S Afr J Mar Sci 11: 537
5	Bustamante et al. 1995	Patella	granularis	patellid limpet	invert	12.138				0.436677						Hatcher	Hatcher	Used chiton value, which is anomalously low
6	Cammen 1980	Nereis	succinea	polychaete	invert	10.57				0.281827						Hatcher	Hatcher	Used Iotrochota sponge value
7	Chapin et al. 1980	.	.	lemmings	mammal	11.6				2.76						Hayssen91 d.	Hayssen91 d. microps	
8	Chew and Chew 1970	Dipodomys	merriami	rodents	mammal	11.6				2.76						Hayssen91 d.	Hayssen91 d. microps	
8	Chew and Chew 1970	Onychomys	torridus	rodents	mammal	11.6				2.76						Hayssen91 d.	Hayssen91 d. microps	
8	Chew and Chew 1970	Lepus	californicus	rodents	mammal	11.6				2.76						Hayssen91 d.	Hayssen91 d. microps	
8	Chew and Chew 1970	Peromyscus	eremicus	rodents	mammal	11.6				2.76						Hayssen91 d.	Hayssen91 d. microps	
8	Chew and Chew 1970	Ammospermo	harrisii	rodents	mammal	11.6				2.76						Hayssen91 d.	Hayssen91 d. microps	
8	Chew and Chew 1970	Spermophilus	spilosoma	rodents	mammal	11.6				2.76						Hayssen91 d.	Hayssen91 d. microps	
8	Chew and Chew 1970	Sylvilagus	audubonii	rodents	mammal	11.6				2.76						Hayssen91 d.	Hayssen91 d. microps	
8	Chew and Chew 1970	Peromyscus	flavus	rodents	mammal	11.6				2.76						Hayssen91 d.	Hayssen91 d. microps	
8	Chew and Chew 1970	Peromyscus	maniculatus	rodents	mammal	11.6				2.76						Hayssen91 d.	Hayssen91 d. microps	
8	Chew and Chew 1970	Reithrodontom	megalotis	rodents	mammal	11.6				2.76						Hayssen91 d.	Hayssen91 d. microps	
8	Chew and Chew 1970	Neotoma	albigula	rodents	mammal	11.6				2.76						Hayssen91 d.	Hayssen91 d. microps	
8	Chew and Chew 1970	Perognathus	baileyi	rodents	mammal	11.6				2.76						Hayssen91 d.	Hayssen91 d. microps	
8	Chew and Chew 1970	Perognathus	penicillatus	rodents	mammal	11.6				2.76						Hayssen91 d.	Hayssen91 d. microps	
9	Collins and Steinauer 1998, unpubl. Data																	
10	Coveney et al. 1977	Daphnia	pulex	zooplankton	fw zoop	8.46817631	9.92	7.06833333	0.79453088	1.19122024	1.55	0.81	0.21287079	9.18964371	0.97977679	Elser et al. 20	Elser et al. 20	From a sample of 12 Daphnia species from different publications
10	Coveney et al. 1977	Eudiaptomus	graciloides	zooplankton	fw zoop	9.91111111	11.1	6.9	1.58137306	0.76833333	1.07	0.45	0.2402013	9.18964371	0.97977679	Elser et al. 20	Elser et al. 20	From a sample of 6 calanoid species from different publications
63	Cyr and Pace 1993a	Daphnia	pulex		fw zoop	8.25	9.92	7.06833333	1.4872299	1.01571429	1.17714286	0.81	0.18753503	9.42555556	1.21156085	Elser et al. 20	Elser et al. 20	Jarrvis 1986 J Plankton Res 8:1065 Mean of 3 D. pulex/pulicaria measurements
63	Cyr and Pace 1993a	Ceriodaphnia	reticulata		fw zoop	8.7	8.7	8.7	0	2.05	2.05	2.05	0	9.42555556	1.21156085	Elser et al. 20	Elser et al. 20	Jarrvis 1986 J Plankton Res 8:1065
63	Cyr and Pace 1993a	Daphnia	longispina		fw zoop	9.25	9.25	9.25	0	1.51	1.51	1.51	0	9.42555556	1.21156085	Elser et al. 20	Elser et al. 20	Jarrvis 1986 J Plankton Res 8:1065
63	Cyr and Pace 1993a	Bosmina	longirostris		fw zoop	8.815	8.815	8.815	0	0.615	0.615	0.615	0	9.42555556	1.21156085	Elser et al. 20	Elser et al. 20	Jarrvis 1986 J Plankton Res 8:1065
63	Cyr and Pace 1993b	Diaphanosoma	brachyurum	cladocerans	fw zoop	9.8	10	9.6	0.28284271	1.085	1.22	0.95	0.19091883	9.42555556	1.21156085	Elser et al. 20	Elser et al. 20	Gophen 1981 J Plankton Res 3: 15
63	Cyr and Pace 1993b	Bosmina	longirostris	cladocerans	fw zoop	8.815	8.815	8.815	0	0.615	0.615	0.615	0	9.42555556	1.21156085	Elser et al. 20	Elser et al. 20	Gophen 1981 J Plankton Res 3: 15
63	Cyr and Pace 1993b	Ceriodaphnia	reticulata	cladocerans	fw zoop	8.7	8.7	8.7	0	2.05	2.05	2.05	0	9.42555556	1.21156085	Elser et al. 20	Elser et al. 20	Gophen 1981 J Plankton Res 3: 15
63	Cyr and Pace 1993b	Mesocyclops	leuckarti	cop	fw zoop	10.15	10.4	9.9	0.35355339	0.905	1.16	0.65	0.36062446	9.42555556	1.21156085	Elser et al. 20	Elser et al. 20	Gophen 1981 J Plankton Res 3: 15
63	Cyr and Pace 1993b	Brachionus	.	rotifera	fw zoop	12.35	12.4	12.3	0.07071068	1.05833333	1.05833333	1.05833333	0	9.42555556	1.21156085	Elser et al. 20	Elser et al. 20	Gophen 1981 J Plankton Res 3: 15 Mean of 2 Brachionus species for N, Synchaeta (the only rotifer) for P
64	Cyr and Pace 1993a	Daphnia	pulex		fw zoop	8.25	9.92	7.06833333	1.4872299	1.01571429	1.17714286	0.81	0.18753503	9.42555556	1.21156085	Elser et al. 20	Elser et al. 20	Jarrvis 1986 J Plankton Res 8:1065 Mean of 3 D. pulex/pulicaria measurements
64	Cyr and Pace 1993a	Ceriodaphnia	reticulata		fw zoop	8.7	8.7	8.7	0	2.05	2.05	2.05	0	9.42555556	1.21156085	Elser et al. 20	Elser et al. 20	Jarrvis 1986 J Plankton Res 8:1065
64	Cyr and Pace 1993a	Daphnia	longispina		fw zoop	9.25	9.25	9.25	0	1.51	1.51	1.51	0	9.42555556	1.21156085	Elser et al. 20	Elser et al. 20	Jarrvis 1986 J Plankton Res 8:1065
64	Cyr and Pace 1993a	Bosmina	longirostris		fw zoop	8.815	8.815	8.815	0	0.615	0.615	0.615	0	9.42555556	1.21156085	Elser et al. 20	Elser et al. 20	Jarrvis 1986 J Plankton Res 8:1065
64	Cyr and Pace 1993b	Diaphanosoma	brachyurum	cladocerans	fw zoop	9.8	10	9.6	0.28284271	1.085	1.22	0.95	0.19091883	9.42555556	1.21156085	Elser et al. 20	Elser et al. 20	Gophen 1981 J Plankton Res 3: 15
64	Cyr and Pace 1993b	Bosmina	longirostris	cladocerans	fw zoop	8.815	8.815	8.815	0	0.615	0.615	0.615	0	9.42555556	1.21156085	Elser et al. 20	Elser et al. 20	Gophen 1981 J Plankton Res 3: 15
64	Cyr and Pace 1993b	Ceriodaphnia	reticulata	cladocerans	fw zoop	8.7	8.7	8.7	0	2.05	2.05	2.05	0	9.42555556	1.21156085	Elser et al. 20	Elser et al. 20	Gophen 1981 J Plankton Res 3: 15
64	Cyr and Pace 1993b	Mesocyclops	leuckarti	cop	fw zoop	10.15	10.4	9.9	0.35355339	0.905	1.16	0.65	0.36062446	9.42555556	1.21156085	Elser et al. 20	Elser et al. 20	Gophen 1981 J Plankton Res 3: 15
64	Cyr and Pace 1993b	Brachionus	.	rotifera	fw zoop	12.35	12.4	12.3	0.07071068	1.05833333	1.05833333	1.05833333	0	9.42555556	1.21156085	Elser et al. 20	Elser et al. 20	Gophen 1981 J Plankton Res 3: 15 Mean of 2 Brachionus species for N, Synchaeta (the only rotifer) for P
11	Frangi and Lugo 1985	.	.	caterpillars	insect	8.564			1.0937	0.9347			0.34404			Fagan lepidopt	Fagan lepidopt	
12	Garnier and Mourelatos 1991	Daphnia	cucullata	cladocerans	fw zoop	9	9	9	0	1.55	1.55	1.55	0	9.10556437	1.2687963	Elser et al. 20	Elser et al. 2000	
12	Garnier and Mourelatos 1991	Daphnia	hyalina	cladocerans	fw zoop	8.9	8.9	8.9	0	1.395	1.395	1.395	0	9.10556437	1.2687963	Elser et al. 20	Elser et al. 2000	
12	Garnier and Mourelatos 1991	Daphnia	galatea	cladocerans	fw zoop	8.21285714	8.21285714	8.21285714	0	1.1375	1.1375	1.1375	0	9.10556437	1.2687963	Elser et al. 20	Elser et al. 2000	
12	Garnier and Mourelatos 1991	Ceriodaphnia	pulchella	cladocerans	fw zoop	8.7	8.7	8.7	0	2.05	2.05	2.05	0	9.10556437	1.2687963	Elser et al. 20	Elser et al. 2000	
12	Garnier and Mourelatos 1991	Ceriodaphnia	dubia	cladocerans	fw zoop	8.7	8.7	8.7	0	2.05	2.05	2.05	0	9.10556437	1.2687963			
12	Garnier and Mourelatos 1991	Diaphanosoma	brachyurum	cladocerans	fw zoop	9.8	10	9.6	0.28284271	1.085	1.22	0.95	0.19091883	9.10556437	1.2687963			
12	Garnier and Mourelatos 1991	Bosmina	longirostris	cladocerans	fw zoop	8.815	8.815	8.815	0	0.615	0.615	0.615	0	9.10556437	1.2687963			
12	Garnier and Mourelatos 1991	Eudiaptomus	gracilis	copepods	fw zoop	9.91111111	11.1	6.9	1.58137306	0.76833333	1.07	0.45	0.2402013	9.10556437	1.2687963			
12	Garnier and Mourelatos 1991	Eurytemora	velox	copepods	fw zoop	9.91111111	11.1	6.9	1.58137306	0.76833333	1.07	0.45	0.2402013	9.10556437	1.2687963			
13	Golley et al. 1962	Aglaopteryx	diaphana	blattodea	insect	10.816			1.1839	0.6503			0.1141			Fagan all orth	Fagan all orthoptera	
13	Golley et al. 1962	Gryllidae	.	crickets	insect	10.816			1.1839	0.6503			0.1141			Fagan all orth	Fagan all orthoptera	
13	Golley et al. 1962	Melampus	coffeus	snail	invert	9.1				0.566751						Hatcher, Turbo torquatus		
13	Golley et al. 1962	Littorina	anglepifera	snail	invert	9.1				0.566751						Hatcher, Turbo torquatus		
13	Golley et al. 1962	Aratus	pisonii	crabs	invert	6.93333333	7.9	5.5	0.904802	1.40166667	7.9	5.5	0.904802			Lim+Harayama, for larval crabs		
13	Golley et al. 1962	Eurytium	limosum	crabs	invert	6.93333333	7.9	5.5	0.904802	1.40166667	7.9	5.5	0.904802			Lim+Harayama, for larval crabs		
13	Golley et al. 1962	Uca	mordax	crabs	invert	6.93333333	7.9	5.5	0.904802	1.40166667	7.9	5.5	0.904802			Lim+Harayama, for larval crabs		
13	Golley et al. 1962	Ucides	cordatus	crabs	invert	6.93333333	7.9	5.5	0.904802	1.40166667	7.9	5.5	0.904802			Lim+Harayama, for larval crabs		
13	Golley et al. 1962	Goniopsis	eventata	crabs	invert	6.93333333	7.9	5.5	0.904802	1.40166667	7.9	5.5	0.904802			Lim+Harayama, for larval crabs		
14	Greenway 1976	Sparisoma	radians	fish	fish													
15	Grier and Logan 1977	.	.	caterpillars	insect	8.564			1.0937	0.9347			0.34404			Fagan lepidopt	Fagan lepidopt	
16	Grier and Logan 1977	.	.	caterpillars	insect	8.564			1.0937	0.9347			0.34404			Fagan lepidopt	Fagan lepidopt	
17	Grier and Logan 1977	.	.	caterpillars	insect	8.564			1.0937	0.9347			0.34404			Fagan lepidopt	Fagan lepidopt	
18	Grier and Logan 1977	.	.	caterpillars	insect	8.564			1.0937	0.9347			0.34404			Fagan lepidopt	Fagan lepidopt	
19	Grier and Logan 1977	.	.	caterpillars	insect	8.564			1.0937	0.9347			0.34404			Fagan lepidopt	Fagan lepidopt	
20	Gruner, age 1400000			mostly orthopt	insect	10.931664				0.6617791								
21	Gruner, age 150000			mostly orthopt	insect	10.931664				0.6617791								
22	Gruner, age 300			mostly orthopt	insect	10.931664				0.6617791								
23	Gruner, age 4100000			mostly orthopt	insect	10.931664				0.6617791								
24	Gruner, age 5000			mostly orthopt	insect	10.931664				0.6617791								
25	Gulati 1984	Bosmina	coregoni	cladocerans	fw zoop	8.815	8.815	8.815	0	0.615	0.615	0.615	0	9.87352519	1.13326956			rare species ignored
25	Gulati 1984	Chydorus	sphaericus	.	fw zoop	8.1665	8.1665	8.1665	0	2.49	2.49	2.49	0	9.87352519	1.13326956			rare species ignored, used Scapholebris
25	Gulati 1984	Daphnia	.	cladocerans	fw zoop	8.46817631	9.92	7.06833333	0.79453088	1.19122024	1.55	0.81	0.21287079	9.87352519	1.13326956			rare species ignored
25	Gulati 1984	Bosmina	longirostris	cladocerans	fw zoop	8.815	8.815	8.815	0	0.615	0.615	0.615	0	9.87352519	1.13326956			rare species ignored
25	Gulati 1984	Cyclops	.	copepoda	fw zoop	10.15	10.4	9.9	0.35355339	0.905	1.16	0.65	0.36062446	9.87352519	1.13326956			rare species ignored, used Mesocyclops edax
25	Gulati 1984	Keratella	cochlearis	rotifera	fw zoop	12.35	12.4	12.3	0.07071068	1.05833333	1.05833333	1.05833333	0	9.87352519	1.13326956			rare species ignored
25	Gulati 1984	Polyarthra	.	rotifera	fw zoop	12.35	12.4	12.3	0.07071068	1.05833333	1.05833333	1.05833333	0	9.87352519	1.13326956			rare species ignored
26	Harris et al. 1975	.	.	canopy arthro	insect	9.1875			1.9827	0.795			0.2501			Fagan all insec	Fagan all insec	
27	Haukioja and Koponen 1975																	
28	Hauxwell et al. 2002: Hauxwe	Microdeutopus	gryllotalpa	amphipod	invert	7.28			0.68	1.01			0.11			Frost et al	Frost et al	used freswhater amphipods from Frost et al

28	Hauxwell et al. 2002: Hauxwe	Cymandusa	compta	amphipod	invert	7.28			0.68	1.01			0.11			Frost et al	Frost et al	used freswhater amphipods from Frost et al
28	Hauxwell et al. 2002: Hauxwe	Idotea	baltica	isopod	invert	7.28			0.68	1.01			0.11			Frost et al	Frost et al	used freswhater amphipods from Frost et al
29	Hauxwell et al. 2002; Hauxwe	Microdeutopus	gryllotalpa	amphipod	invert	7.28			0.68	1.01			0.11			Frost et al	Frost et al	used freswhater amphipods from Frost et al
29	Hauxwell et al. 2002; Hauxwe	Cymandusa	compta	amphipod	invert	7.28			0.68	1.01			0.11			Frost et al	Frost et al	used freswhater amphipods from Frost et al
29	Hauxwell et al. 2002: Hauxwe	Idotea	baltica	isopod	invert	7.28			0.68	1.01			0.11			Frost et al	Frost et al	used freswhater amphipods from Frost et al
30	Jansson et al. 1984	protozoans	.	.	zoop	6.99943452	8.61230329	5.49019608	0.80682275	1.29091027	2.58333333	0.4434907	0.62454483	8.45527282	1.0296218	GoldmanEtAlM	GoldmanEtAlM	from a marine micro-flagellate
30	Jansson et al. 1984	copepods	.	mesozoop	zoop	9.91111111	11.1	6.9	1.58137306	0.76833333	1.07	0.45	0.2402013	8.45527282	1.0296218			used mean calanoid
31	Jespersen et al. 1988	Daphnia	cucullata	zooplankton	fw zoop	9	9	9	0	1.55	1.55	1.55	0	8.97	1.35583333			
31	Jespersen et al. 1988	Eudiaptomus	graciloides	zooplankton	fw zoop	9.91111111	11.1	6.9	1.58137306	0.76833333	1.07	0.45	0.2402013	8.97315278	1.35583333			
31	Jespersen et al. 1988	Bosmina	coregoni	zooplankton	fw zoop	8.815	8.815	8.815	0	0.615	0.615	0.615	0	8.97315278	1.35583333			
31	Jespersen et al. 1988	Chydorus	sphaericus	zooplankton	fw zoop	8.1665	8.1665	8.1665	0	2.49	2.49	2.49	0	8.97315278	1.35583333			rare species ignored, used Scapholebris
32	Klumpp et al. 1989	.	.	parrotfish	fish	12.5				2.5								compilation -- many herbivores listed, lots not very specific
32	Klumpp et al. 1989	.	.	garfish	fish	12.5				2.5								compilation -- many herbivores listed, lots not very specific
32	Klumpp et al. 1989	Scaridae	.		fish	12.5				2.5								compilation -- many herbivores listed, lots not very specific
32	Klumpp et al. 1989	Sparidae	.		fish	12.5				2.5								compilation -- many herbivores listed, lots not very specific
32	Klumpp et al. 1989	Monacanthidae	.		fish	12.5				2.5								compilation -- many herbivores listed, lots not very specific
32	Klumpp et al. 1989	Hemirhamphic	.		fish	12.5				2.5								compilation -- many herbivores listed, lots not very specific
32	Klumpp et al. 1989	Haemuliidae	.		fish	12.5				2.5								compilation -- many herbivores listed, lots not very specific
32	Klumpp et al. 1989	Atherinidae	.		fish	12.5				2.5								compilation -- many herbivores listed, lots not very specific
32	Klumpp et al. 1989	Blenniidae	.		fish	12.5				2.5								compilation -- many herbivores listed, lots not very specific
32	Klumpp et al. 1989	Gobiidae	.		fish	12.5				2.5								compilation -- many herbivores listed, lots not very specific
32	Klumpp et al. 1989	Tetraodontidae	.		fish	12.5				2.5								compilation -- many herbivores listed, lots not very specific
32	Klumpp et al. 1989	echinoids	.	sea urchin	invert	10.57				0.281827						Hatcher	Hatcher	compilation -- many herbivores listed, lots not very specific- Used data for Iotrochota sponges
32	Klumpp et al. 1989	.	.	crustaceans	invert	6.93333333	7.9	5.5	0.904802	1.40166667	7.9	5.5	0.904802			Lim+Harayam	Lim+Harayam	compilation -- many herbivores listed, lots not very specific, average of crab larval stages
32	Klumpp et al. 1989	.	.	molluscs	invert	8.5348387	9.52380952	7.52688172	0.78820196	0.83951371	1.29041667	0.54987551	0.2593176			Smaal et al	Smaal et al.	Mytilus edulis
32	Klumpp et al. 1989	.	.	crabs	invert	6.93333333	7.9	5.5	0.904802	1.40166667	7.9	5.5	0.904802					compilation -- many herbivores listed, lots not very specific
32	Klumpp et al. 1989	.	.	isopods	invert	7.28			0.68	1.01			0.11			Frost et al	Frost et al	used freswhater amphipods from Frost et al
32	Klumpp et al. 1989	.	.	amphipod	invert	7.28			0.68	1.01			0.11			Frost et al	Frost et al	used freswhater amphipods from Frost et al
32	Klumpp et al. 1989	Caridae	.		invert													compilation -- many herbivores listed, lots not very specific
32	Klumpp et al. 1989	Gastropoda	.		invert	9.1				0.566751						Hatcher	Hatcher	compilation -- many herbivores listed, lots not very specific
32	Klumpp et al. 1989	Penaeidae	.		invert	11.1				1.39						Uye + Matsuda	Uye + Matsuda	compilation -- many herbivores listed, lots not very specific
32	Klumpp et al. 1989	Polychaeta	.		invert	11.1				1.39						Uye + Matsuda	Uye + Matsuda	compilation -- many herbivores listed, lots not very specific
32	Klumpp et al. 1989	Mytilid	.	bivalve	invert	11.1				1.39						Uye + Matsuda	Uye + Matsuda	compilation -- many herbivores listed, lots not very specific
33	Klumpp et al. 1989	.	.	parrotfish	fish	12.5				2.5								compilation -- many herbivores listed, lots not very specific
33	Klumpp et al. 1989	.	.	garfish	fish	12.5				2.5								compilation -- many herbivores listed, lots not very specific
33	Klumpp et al. 1989	Scaridae	.		fish	12.5				2.5								compilation -- many herbivores listed, lots not very specific
33	Klumpp et al. 1989	Sparidae	.		fish	12.5				2.5								compilation -- many herbivores listed, lots not very specific
33	Klumpp et al. 1989	Monacanthidae	.		fish	12.5				2.5								compilation -- many herbivores listed, lots not very specific
33	Klumpp et al. 1989	Hemirhamphic	.		fish	12.5				2.5								compilation -- many herbivores listed, lots not very specific
33	Klumpp et al. 1989	Haemuliidae	.		fish	12.5				2.5								compilation -- many herbivores listed, lots not very specific
33	Klumpp et al. 1989	Atherinidae	.		fish	12.5				2.5								compilation -- many herbivores listed, lots not very specific
33	Klumpp et al. 1989	Blenniidae	.		fish	12.5				2.5								compilation -- many herbivores listed, lots not very specific
33	Klumpp et al. 1989	Gobiidae	.		fish	12.5				2.5								compilation -- many herbivores listed, lots not very specific
33	Klumpp et al. 1989	Tetraodontidae	.		fish	12.5				2.5								compilation -- many herbivores listed, lots not very specific
33	Klumpp et al. 1989	echinoids	.	sea urchin	invert	10.57				0.281827						Hatcher	Hatcher	compilation -- many herbivores listed, lots not very specific- Used data for Iotrochota sponges
33	Klumpp et al. 1989	.	.	crustaceans	invert	6.93333333	7.9	5.5	0.904802	1.40166667	7.9	5.5	0.904802			Lim+Harayam	Lim+Harayam	compilation -- many herbivores listed, lots not very specific, average of crab larval stages
33	Klumpp et al. 1989	.	.	molluscs	invert	8.5348387	9.52380952	7.52688172	0.78820196	0.83951371	1.29041667	0.54987551	0.2593176			Smaal et al	Smaal et al.	Mytilus edulis
33	Klumpp et al. 1989	.	.	crabs	invert	6.93333333	7.9	5.5	0.904802	1.40166667	7.9	5.5	0.904802					compilation -- many herbivores listed, lots not very specific
33	Klumpp et al. 1989	.	.	isopods	invert	7.28			0.68	1.01			0.11			Frost et al	Frost et al	used freswhater amphipods from Frost et al
33	Klumpp et al. 1989	.	.	amphipod	invert	7.28			0.68	1.01			0.11			Frost et al	Frost et al	used freswhater amphipods from Frost et al
33	Klumpp et al. 1989	Caridae	.		invert													compilation -- many herbivores listed, lots not very specific
33	Klumpp et al. 1989	Gastropoda	.		invert	9.1				0.566751						Hatcher	Hatcher	compilation -- many herbivores listed, lots not very specific
33	Klumpp et al. 1989	Penaeidae	.		invert	11.1				1.39						Uye + Matsuda	Uye + Matsuda	compilation -- many herbivores listed, lots not very specific
33	Klumpp et al. 1989	Polychaeta	.		invert	11.1				1.39						Uye + Matsuda	Uye + Matsuda	compilation -- many herbivores listed, lots not very specific
33	Klumpp et al. 1989	Mytilid	.	bivalve	invert	11.1				1.39						Uye + Matsuda	Uye + Matsuda	compilation -- many herbivores listed, lots not very specific
34	Mann 1972 (averages for sea	.	.	sea urchin	invert	10.57				0.281827						Hatcher	Hatcher	Used data for Iotrochota sponges
65	McNaughton 1985	Connochaetes	taurinus	wildebeest	mammal	9.6				2.88						Dierenfeld07 t	Gillooly&Allen05	bull
65	McNaughton 1985	Equus	burchelli	zebra	mammal	9.6				2.88						Dierenfeld07 t	Gillooly&Allen05	bull
65	McNaughton 1985	Taurotragus	oryx	eland	mammal	9.6				2.88						Dierenfeld07 t	Gillooly&Allen05	bull
65	McNaughton 1985	Gazella	thomsonii	Thompson's ga	mammal	9.6				2.88						Dierenfeld07 t	Gillooly&Allen05	bull
65	McNaughton 1985	Syncerus	caffer	buffalo	mammal	9.6				2.88						Dierenfeld07 t	Gillooly&Allen05	bull
65	McNaughton 1985	Damaliscus	korrigum	topi	mammal	9.6				2.88						Dierenfeld07 t	Gillooly&Allen05	bull
65	McNaughton 1985	Connochaetes	taurinus	wildebeest	mammal	9.6				2.88						Dierenfeld07 t	Gillooly&Allen05	bull
65	McNaughton 1985	Equus	burchelli	zebra	mammal	9.6				2.88						Dierenfeld07 t	Gillooly&Allen05	bull
65	McNaughton 1985	Taurotragus	oryx	eland	mammal	9.6				2.88						Dierenfeld07 t	Gillooly&Allen05	bull
65	McNaughton 1985	Gazella	thomsonii	Thompson's ga	mammal	9.6				2.88						Dierenfeld07 t	Gillooly&Allen05	bull
65	McNaughton 1985	Syncerus	caffer	buffalo	mammal	9.6				2.88						Dierenfeld07 t	Gillooly&Allen05	bull
65	McNaughton 1985	Damaliscus	korrigum	topi	mammal	9.6				2.88						Dierenfeld07 t	Gillooly&Allen05	bull
35	McQuaid and Branch 1985-ex	.	.	.														herbiv desc too general
36	McQuaid and Branch 1985-pr	.	.	.														herbiv desc too general
37	Menhinick 1967@					10.931664				0.6617791						Fagan orthopt	Fagan orthopt	Used mean orthopteran from Fagan data set
38	Murray et al. 1994	protozoans	.	.	zoop	6.99943452	8.61230329	5.49019608	0.80682275	1.29091027	2.58333333	0.4434907	0.62454483	8.45527282	1.0296218	GoldmanEtAlM	GoldmanEtAlM	from a marine micro-flagellate
38	Murray et al. 1994	copepods	.	mesozoop	zoop	9.91111111	11.1	6.9	1.58137306	0.76833333	1.07	0.45	0.2402013	8.45527282	1.0296218			used mean calanoid
39	Naiman 1976	Cyprinodon	nevadensis am	pupfish	fish													
40	Nakanishi et al. 1992	Copepoda	nauplii	copepoda	fw zoop	9.91111111	11.1	6.9	1.58137306	0.76833333	1.07	0.45	0.2402013	11.0192369	0.97596154			used mean calanoid
40	Nakanishi et al. 1992	Eadiaptomus	japonicus	copepoda	fw zoop	9.91111111	11.1	6.9	1.58137306	0.76833333	1.07	0.45	0.2402013	11.0192369	0.97596154			used mean calanoid
40	Nakanishi et al. 1992	Mesocyclops	thermocyclopc	copepoda	fw zoop	10.15	10.4	9.9	0.35355339	0.905	1.16	0.65	0.36062446	11.0192369	0.97596154	Elser et al. 20	Elser et al. 20	Gophen 1981 J Plankton Res 3: 15
40	Nakanishi et al. 1992	Daphnia	galeata	cladocerans	fw zoop	8.21285714	8.21285714	8.21285714	0	1.1375	1.1375	1.1375	0	11.0192369	0.97596154			
40	Nakanishi et al. 1992	Bosmina	longirostris	cladocerans	fw zoop	8.815	8.815	8.815	0	0.615	0.615	0.615	0	11.0192369	0.97596154			
40	Nakanishi et al. 1992	Polyarthra	.	rotifera	fw zoop	12.35	12.4	12.3	0.07071068	1.05833333	1.05833333	1.05833333	0	11.0192369	0.97596154			
40	Nakanishi et al. 1992	Keratella	quadrata	rotifera	fw zoop	12.35	12.4	12.3	0.07071068	1.05833333	1.05833333	1.05833333	0	11.0192369	0.97596154			
40	Nakanishi et al. 1992	Ploesoma	truncatum	rotifera	fw zoop	12.35	12.4	12.3	0.07071068	1.05833333	1.05833333	1.05833333	0	11.0192369	0.97596154			
40	Nakanishi et al. 1992	Filinia	longiseta	rotifera	fw zoop	12.35	12.4	12.3	0.07071068	1.05833333	1.05833333	1.05833333	0	11.0192369	0.97596154			
40	Nakanishi et al. 1992	Asplanchna	priodonta	rotifera	fw zoop	12.35	12.4	12.3	0.07071068	1.05833333	1.05833333	1.05833333	0	11.0192369	0.97596154			
40	Nakanishi et al. 1992	Kellicottia	lonispina	rotifera	fw zoop	12.35	12.4	12.3	0.07071068	1.05833333	1.05833333	1.05833333	0	11.0192369	0.97596154			
40	Nakanishi et al. 1992	Keratella	cochlearis	rotifera	fw zoop	12.35	12.4	12.3	0.07071068	1.05833333	1.05833333	1.05833333	0	11.0192369	0.97596154			
40	Nakanishi et al. 1992	Diaphanosoma	brachyurum	cladocerans	fw zoop	9.8	10	9.6	0.28284271	1.085	1.22	0.95	0.19091883	11.0192369	0.97596154			
41	Newell et al. 1982	Pyura	.	ascidian	invert	8.232				0.272536						Hatcher	Hatcher	
41	Newell et al. 1982	Aulacomya	ater	mussel	invert	8.5348387	9.52380952	7.52688172	0.78820196	0.83951371	1.29041667	0.54987551	0.2593176			Smaal et al	Smaal et al.	Mytilus edulis
41	Newell et al. 1982	Tethya	.	sponge	invert	10.57				0.281827						Hatcher	Hatcher	Used data for Iotrochota sponges
41	Newell et al. 1982	Parechinus	angulosus	sea urchin	invert	10.57				0.281827						Hatcher	Hatcher	Used data for Iotrochota sponges
41	Newell et al. 1982	Haliotis	midae	gastropod	invert	9.1				0.566751								
41	Newell et al. 1982	Patella	.	gastropod	invert	9.1				0.566751								
42	Odum 1957	Arcella	.	protozoan	protozoan	6.99943452	8.61230329	5.49019608	0.80682275	1.29091027	2.58333333	0.4434907	0.62454483					Table 7
42	Odum 1957	.	.	mullet	fish													Table 7
42	Odum 1957	chironomid	.		insect	8.6126			1.97687	0.9599			0.2607				Fagan all dipte	Fagan all dipte Table 7
42	Odum 1957	Elophila	.	lepidoptera?	insect	8.564			1.0937	0.9347			0.34404				Fagan lepidopt	Fagan lepidopt Table 7
42	Odum 1957	Hydrobiidae	.	snail	invert	9.1				0.566751								Table 7
42	Odum 1957	Oligochaete	.	polychaete	invert	10.57				0.281827						Hatcher	Hatcher	Used Iotrochota sponge value
42	Odum 1957	.	.	gammarids	invert	7.28			0.68	1.01			0.11			Frost et al	Frost et al	used freswhater amphipods from Frost et al
42	Odum 1957	.	.	flatworms	invert	8.232				0.272536						Hatcher	Hatcher	Table 7, used Ascidian value
42	Odum 1957	paleomonetes	.	shrimp	invert	11.1				1.39						Uye and Matsu	Uye and Matsu	Table 7, mysids
42	Odum 1957	Pomacea	.	snail	invert	9.1				0.566751						Hatcher	Hatcher	Table 7
42	Odum 1957	.	.	turtles	reptile													Table 7
42	Odum 1957	.	.	copepods	fw zoop	9.91111111	11.1	6.9	1.58137306	0.76833333	1.07	0.45	0.2402013					used mean calanoid
42	Odum 1957	.	.	ostracods	fw zoop	8.46817631	9.92	7.06833333	0.79453088	1.19122024	1.55	0.81	0.21287079			Elser et al. 20	Elser et al. 20	used mean daphnia
43	Odum et al. 1962*																	
44	Parsons and De la Cruz 1980	Tettigoniidae	.	conocephaline	insect	10.7595			0.0601	0.6727			.			Fagan tettigon	Fagan tettigoniidae	
45	Ritchie, 1989-1999					10.931664				0.6617791						Fagan orthopt	Fagan orthopt	
46	Schowalter et al. 1981	Orthoptera		chewing herbi	insect	10.931664				0.6617791						Fagan orthopt	Fagan orthoptera	
46	Schowalter et al. 1981	Hymenoptera		sucking herbivores		9.338861				0.6632201						Fagan hymenc	Fagan hymenoptera	
47	Scott et al. 1979	Orthoptera	.	insects	insects	10.816			1.1839	0.65034			0.11408			Fagan orthopt	Fagan orthoptera	
47	Scott et al. 1979	Chrysomelidae	.	insects	insects	7.3588			1.81	0.92976			0.21			Fagan chryson	Fagan chrysomelid	
47	Scott et al. 1979	Scarabidae	.	insects	insects	11.35			1.3152	0.6211			.			Fagan scarabic	Fagan scarabidae	

47	Scott et al. 1979	Curculionidae	.	insects	insects	9.8565			0.77428	0.5425			0.09746	Fagan curculio	Fagan curculionid	
47	Scott et al. 1979	Tenebrionidae	.	insects	insects	8			0.4243	1.345			0.10607	Fagan tenebric	Fagan tenebrionid	
47	Scott et al. 1979	Carabidae	.	insects	insects											
47	Scott et al. 1979	Lepidoptera	.	larvae	insects	8.564			1.0937	0.9347			0.34404	Fagan lepidopt	Fagan lepidoptera	
47	Scott et al. 1979	.	.	nematodes	invert	8.232				0.272536				Hatcher, ascidi	Hatcher, ascidians	
48	Sinclair 1975	Connochaetes	taurinus	wildebeest	mammal	9.6				2.88				Dierenfeld07 b	Gillooly&Allen05 bull	
48	Sinclair 1975	Equus	burchelli	zebra	mammal											
48	Sinclair 1975	Gazella	thomsonii	Thompson's ga	mammal	9.6				2.88				Dierenfeld07 b	Gillooly&Allen05 bull	
48	Sinclair 1975	Taurotragus	oryx	eland	mammal	9.6				2.88				Dierenfeld07 b	Gillooly&Allen05 bull	
49	Small et al. 1989	copepods	.	mesozoop	mar zoop	7.73213148	11.1111111	4.02298851	1.93306673	0.82336711	1.64021164	0.29693487	0.29184236			
50	Sorokin 1983	Mugil	cephalus	fish	fish	12.5				2.5						
50	Sorokin 1983	Mugil	saliens	fish	fish	12.5				2.5						
50	Sorokin 1983	Caspialosa	.	fish	fish	12.5				2.5						
50	Sorokin 1983	Clupeonella	.	fish	fish	12.5				2.5						
50	Sorokin 1983	Engraulis	.	fish	fish	12.5				2.5						
50	Sorokin 1983	Sardinella	.	fish	fish	12.5				2.5						
50	Sorokin 1983	Sprattus	sprattus	fish	fish	12.5				2.5						
50	Sorokin 1983	Syngnathus	schmidtii	fish	fish	12.5				2.5						
50	Sorokin 1983	Oikopleura		zooplankton	mar zoop	2.8072	4.93	1.314	1.49286811	0.1305	0.15	0.111	0.02757716			
50	Sorokin 1983	Penilia		zooplankton	mar zoop	8.46817631	9.92	7.06833333	0.79453088	1.19122024	1.55	0.81	0.21287079			used average Daphnia, no marine cladoceran data available
50	Sorokin 1983	Paracalanus		zooplankton	mar zoop	7.73213148	11.1111111	4.02298851	1.93306673	0.82336711	1.64021164	0.29693487	0.29184236			used average marine calanoid
50	Sorokin 1983	Calanus	helgolandicus	zooplankton	mar zoop	7.73213148	11.1111111	4.02298851	1.93306673	0.82336711	1.64021164	0.29693487	0.29184236			used average marine calanoid
50	Sorokin 1983	Pseudocalanus	elongatus	zooplankton	mar zoop	7.73213148	11.1111111	4.02298851	1.93306673	0.82336711	1.64021164	0.29693487	0.29184236			used average marine calanoid
50	Sorokin 1983	Centropages	.	copepods	mar zoop	7.90389332	9.1503268	6.76328502	0.85719415	0.88347763	1.17424242	0.73809524	0.2518097			
50	Sorokin 1983	Pontella	.	copepods	mar zoop	7.73213148	11.1111111	4.02298851	1.93306673	0.82336711	1.64021164	0.29693487	0.29184236			used average marine calanoid
50	Sorokin 1983	Acartia	.	copepods	mar zoop	7.70172639	9.72222222	5.36398467	1.60174235	1.14652077	1.64021164	0.86834734	0.33903071			
51	Teal 1962	Prokelisia	.	hopper	insect	9.5528			0.78997	0.8049			0.01242	Fagan prokelis	Fagan prokelisia	
51	Teal 1962	Orchelimum	.	katydid??	insect	10.7595			0.0601	0.6727			.	Fagan tettigon	Fagan tettigoniidae	
51	Teal 1962	Uca	.	crabs	invert	6.93333333	7.9	5.5	0.904802	1.40166667	7.9	5.5	0.904802	Lim + Harayar	Lim + Harayama, larval crabs	
51	Teal 1962	Sesarma	.	crabs	invert	6.93333333	7.9	5.5	0.904802	1.40166667	7.9	5.5	0.904802	Lim + Harayar	Lim + Harayama, larval crabs	
51	Teal 1962	Modiolus	.	mussel	invert	8.5348387	9.52380952	7.52688172	0.78820196	0.83951371	1.29041667	0.54987551	0.2593176	Smaal et al. m	Smaal et al, mytilus edulis	
51	Teal 1962	Littorina	.	molluscs	invert	9.1				0.566751				Hatcher, gastr	Hatcher, gastropod	
51	Teal 1962	Oligochaete	.	polychaete	invert	10.57				0.281827				Hatcher	Hatcher	Used Iotrochota sponge value
51	Teal 1962	Streblospio	.	polychaete	invert	10.57				0.281827				Hatcher	Hatcher	Used Iotrochota sponge value
51	Teal 1962	Capitella	.	polychaete	invert	10.57				0.281827				Hatcher	Hatcher	Used Iotrochota sponge value
51	Teal 1962	Manayunkia	.	polychaete	invert	10.57				0.281827				Hatcher	Hatcher	Used Iotrochota sponge value
52	Van Hook 1971	Melanoplus	sanguinipes	Acrididae	insect	10.9713			0.79796	0.65395			0.1384	Fagan melanop	Fagan melanoplus	
52	Van Hook 1971	Conocephalus	fasciatus	Tettigoniidae	insect	10.7595			0.0601	0.6727			.	Fagan tettigon	Fagan tettigoniidae	
52	Van Hook 1971	.	.	Homoptera/ H	insect	8.981			2.1489	0.8503			0.18719	Fagan hemipte	Fagan hemiptera	
52	Van Hook 1971	Pteronemobius	fasciatus	Gryllidae (omr	insect	10.816			1.1839	0.6503			0.1141	Fagan all ortho	Fagan all orthoptera	
53	Weisse et al 1990	Mesocyclops	leuckarti	zooplankton	invert	10.15	10.4	9.9	0.35355339	0.905	1.16	0.65	0.36062446			
53	Weisse et al 1990	Cyclops	vicinus	zooplankton	invert	10.85	10.85	10.85	0	1.35	1.35	1.35	0	Elser et al. 20	Elser et al. 20	used Diacyclops thomasi
53	Weisse et al 1990	Daphnia	hyalina	zooplankton	invert	8.21285714	8.21285714	8.21285714	0	1.1375	1.1375	1.1375	0			
53	Weisse et al 1990	Daphnia	galeata	zooplankton	invert	8.21285714	8.21285714	8.21285714	0	1.1375	1.1375	1.1375	0			

LITERATURE USED FOR THE COMPILATION OF THE DATA SETS

Admiraal, W., Bouwman, L. A., Hoekstra, L. & Romeyn, K. Qualitative and Quantitative Interactions between Microphytobenthos and Herbivorous Meiofauna on a Brackish Intertidal Mudflat. Int. Revue ges. Hydrobiol. 68, 175-191 (1983)

Aerts R (1989) Aboveground biomass and nutrient dynamics of Calluna vulgaris and Molinia caerulea in a dry heathland. Oikos 56: 31-38

Aerts, R., B.S. Waller and N. Malmer. 1992. Growth limiting nutrients in Sphagnum dominated bogs subject to low and high atmospheric nitrogen supply. J. Ecology 80: 131-140

Aizaki M, Takamura N (1991) Regeneration of nutrient and detritus formation from aerobic decomposition of natural phytoplankton. Jpn J Limnol 52:83-94

Albright LJ, Chocair J, Masuda K, Valdes M (1980) In situ degradation of the kelps Macrocystis integrifolia and Nereocystis luetkeana in British Columbia coastal waters. Nat Can 107: 3-10

Andersen FO (1978) Effects of nutrient level on the decomposition of Phragmites communis Trin Arch Hydrobiol 84: 42-54.

Andersen, F. and E. Kristensen. 1988. The influence of macrofauna on estuarine benthic community metabolism: a microcosm study. Mar. Biol. 99:591-603.

Anderson, M. G. Use of sago pondweed by waterfowl on the Delta Marsh, Manitoba. J. Wildl. Manage. **40**, 233-242 (1976)

Anderson, M.R., Cardinal A. and Larochelle J. (1981). An alternate growth pattern for Laminaria longicruris. J. Phycol. 17: 405-411.

Arndt, H., M. Kroker, B. Nixdorf, and A.Kohler. 1993. Long-term annual and seasonal changes of meta- and protozooplankton in Lake Muggelsee (Berlin): Effects of eutrophication grazing activities, and the impact of predation. Int. Revue ges. Hydrobiol. 78:379-402.

Atkinson, M. J., R. W. Grigg (1984). Model of a coral reef ecosystem: II. Gross and net benthic primary production at French Frigate Shoals, Hawaii. Coral Reefs 3: 13-22

Auclair, A. N. D., Bouchard, A. & Pajaczkowski, J. Productiviry in a Carex-dominated ecosystem. Oecologia **26**, 9-31 (1976)

Aziz, S.A and D.B. Nedwell. 1986. The nitrogen cycle of an east coast, U.K. saltmarsh: I. Nitrogen assimilation during primary production: Detrital mineralization. Est. Coast. Shelf Sci. 22: 559-575

Bach, S. D., M. N. Josselyn (1979). Production and biomass of Cladophora prolifera (Chlorophyta, Cladophorales) in Bermuda. Bot. Mar. 22: 163-168.

Bach, S., G. Thayer and M. LaCroix 1986. Export of detritus from eelgrass (Zostera marina) beds near Beaufort, North Carolina, USA. Mar. Ecol. Prog. Ser. 28: 265-278.

Baird, D. and R. E. Ulanowicz. 1989. The Seasonal Dynamics of the Chesapeake Bay Ecosystem. Ecol. Monogr. 59: 329-364.

Baird, D. and R. E. Ulanowicz. 1993. Comparative study on the trophic structure, cycling and ecosystem properties of four tidal estuaries. Mar. Ecol. Prog. Ser. 99: 221-237.

Bak, R. 1994. Sea urchin bioerosion on coral reefs: place in the carbonate budget and relevant variables. Coral Reefs 13: 99-103.

Baldwin, J. R. and J. R. Lovvorn. 1994. Expansion of seagrass habitat by the exotic Zostera japonica and its use by dabbling ducks and brant in Boundary Bay, British Columbia. Mar. Ecol. Prog. Ser. 103: 119-127.

Barko, J. W., Murphy, P. G. & Wetzel, R. G. An investigation of primary production and ecosystem metabolism in a Lake Michigan dune pond. Arch. Hydrobiol. **81**, 155-187 (1977).

Bastardo H (1979). Laboratory studies on decomposition of littoral plants. Arch. Hydrobiol. 26: 267-299.

Bayley SE, Zoltek J Jr, Hermann AJ, Dolan TJ, Tortora L (1985). Experimental manipulation of nutrients and water in a freshwater marsh: effects on biomass, decomposition, and nutrient accumulation. Limnol. Oceanogr 30: 500-512.

Behrendt, H. 1990. The chemical composition of phytoplankton and zooplankton in an eutrophic shallow lake. Arch. Hydrobiol. 118:129-145.

Benner R, Fogel ML, Sprague EK (1991) Diagenesis of belowground biomass of Spartina alterniflora in salt-marsh sediments. Limnol. Oceanogr. 36: 1358-1374.

Bernard, J.M and B.A. Solsky. 1977. Nutrient cycling in a *Carex lacustris* wetland. Can. J. Bot. 55: 630-638

Best EPH, Dassen JHA, Boon JJ, Wiegers G (1990). Studies on decomposition of Ceratophyllum demersum litter under laboratory and field conditions: losses of dry mass and nutrients, qualitative changes in organic compounds and consequences for ambient water and sediments. Hydrobiologia 194: 91-114.

Biddanda BA (1988) Microbial aggregation and degradation of phytoplankton-derived detritus in seawater. II. Microbial metabolism. Mar Ecol Prog Ser 42: 89-95

Bienfag, P. and D. Zieman. 1992. The role of Coastal High Latitude Ecosystems in Global Export Production , p. 285-298. *In* P. Falkowski and A. Woodhead [eds], Primary Productivity and Biogeochemical Cycles in the Sea. Plenum Press.

Billings, W. D. Carbon balance of Alaskan tundra and taiga ecosystem: past, present and future. Quat. Sci. Rev. **6**, 165-177 (1987).

Birch PB, Gabrielson JO, Hamel KS (1983) Decomposition of Cladophora. I Field studies in the Peel-Harvey estuarine system, Westen Australia. Bot Mar 26: 165-171

Bishop, J.K.B., J.M. Edmund, D.R. Ketter, M.P. Baeen and W.B. Silver. 1977. The chemistry, biology and vertical flux of particulate matter from the upper 400m of the equatorial Atlantic Ocean. Deep-Sea Res. 24: 511-548

Bliss, L. C. in Structure and Function of Tundra Ecosystems (eds Rosswall, T. & Heal, O.) 17-60 (Tundra Biome Steering Committee, Stockholm, 1975).

Blum, L. 1993. Spartina alterniflora root dynamics in a Virginia marsh. Mar. Ecol. Prog. Ser. 102:169-178.

Boring, L.R., C.D. Monk, and W.T. Swank (1981) Early regeneration of a clear-cut southern Appalachian forest. Ecology 62(5):1244-1253

Boyd, C.E. 1970. Production, mineral accumulation and pigment concentrations in *Typha latifolia* and *Scirpus americanus* Ecology 51: 285-290

Boyd, C.E. 1971. The dynamics of dry matter and chemical substances in a *Juncus effusus* population. Am. Midl. Nat. 86: 28-45

Boyer, M.L.H. and B.D. Wheeler. 1989. Vegetation patterns in spring-fed calcareous fens: calcite precipitation and constraints on fertility. J. Ecology 77: 597-609

Bray, J. R. Primary consumption in three forest canopies. Ecology, **45**, 165-167 (1964).

Breteler RJ, Teal JM (1981) Trace elements enrichments in decomposing litter of Spartina alterniflora. Aquat. Bot. 11: 111-120

Briggs SV, Maher MT, Tongway DJ (1985) Dry matter and nutrient loss from decomposing Vallisneria spiralis L. Aquat. Bot. 22: 387-392.

Brouwer PEM. 1996. Decomposition in situ of the sublittoral Antartic macroalgae Desmarestia anceps Montagne. Polar Biol. 16: 129-137

Brown S, Flohrschutz EW, Odum HT. 1984. Structure, productivity and phosphorus cycling of the scrub cypress ecosystem. In: KC Ewel and HT Odum (eds): "Cypress swamps", University Presses of Florida. Gainesville, Fla. pp. 304-317

Burns, K., M. Ehrhardt, B. Hows and G. Taylor. 1993. Subtidal Benthic Community Respiration and production near the heavily oiled Gulf Coast of Saudi Arabia. Mar. Pollut. Bul. 27: 199-205.

Bustamante, R. H., Branch, G. M., Eekhout, S., Robertson, B., Zoutendyk, P., Schleyer, M., Dye, A., Hanekom, N., Keats, D., Jurd, M. and McQuaid, C. 1995. Gradients of intertidal primary productivity around the coast of South Africa and their relationships with consumer biomass. Oecologia 102: 189-201.

Buth, G. 1987. Decomposition of roots of three plant communities in a dutch salt marsh. Aquat. Bot. 29:123-128.

Bythell, J., E. Gladfelter and M. Bythell. 1993. Chronic and catastrophic natural mortality of three common Caribbean reef corals. Coral Reefs 12:143-152.

Cadee, G. C., J. Hegeman (1977). Distribution of primary production of the benthic microflora and accumulation of organic matter on a tidal flat area, Balgzand, Dutch Wadden Sea. Neth. J. Sea Res. 11: 24-41

Cahoon, D. and J. Stevenson. 1986. Production, predation and decomposition in a low-salinity Hibischus marsh. Ecology 67:1341-1350.

Cammen, L. M. 1980. The significance of microbial carbon in the nutrition of the deposit feeding polychaete Nereis succinea. Mar. Biol. 61: 9-20.

Carpenter, R. C. Partitioning herbivory and its effects on coral reef algal communities. Ecol. Monogr. 56, 345-363 (1986).

Cattaneo, A. Grazing on epiphytes. Limnol. Oceanogr. **28**, 124-132 (1983).

Cebrian, J and C.M. Duarte. 1998. Patterns in leaf herbivory on seagrasses. Aquatic Botany 60: 67-82

Cebrian, J., C. M. Duarte, N. Marbà, S. Enríquez, M. Gallegos and B. Olesen. 1996. Herbivory on *Posidonia oceanica* (L.) Delile: magnitude and variability in the Spanish Mediterranean. Mar. Ecol. Progr. Ser. 130: 147-155.

Cebrián, J., C. M. Duarte, N. Marbà and S. Enríquez. 1997. The magnitude and fate of production of four co-ocurring Western Mediterranean seagrass species. Mar. Ecol. Progr. Ser. 155 : 29-44

Cebrian J., M. F. Pedersen, K. Kroeger, I. Valiela. 2000. Fate of production of the seagrass Cymodocea nodosa in different stages of meadow formation. Mar. Ecol. Prog. Ser. 204:119-130

Chapin III, F. S., P. C. Miller, W. D. Billings and P. I Coyne. (1980). Carbon and nutrient budgets and their control in coastal tundra. In Brown, J., P. C. Miller, L. L. Tieszen and

F. L. Bunnell (eds). An Arctic Ecosystem: The Coastal Tundra at Barrow, Alaska. Chapter 12, pp. 458-482. Dowden, Hutchinson and Ross Stroudsburg, Pennsylvania.

Chapman ARO, Craigie JS. 1978. Seasonal growth in Laminaria longicruris: relations with reserve carbohydrate storage and production. Mar. Biol. 46: 209-213

Chavez, F., K. Buck, K. Coale, J. Martin, G. DiTullio, N. Welschmeyer, A. Jacobsen and R. Barber. 1991. Growth rates, grazing, sinking and iron limitation of equatorial pacific phytoplankton. Limnol. Oceanogr. 36: 1816-1833.

Chew, R. M. & Chew, A. E. Energy relationships of the mammals of a desert shrub (Larrea tridentata) community. Ecol. Monogr. **40**, 1-21 (1970).

Christensen, B. (1978). Biomass and primary production of Rhizophora apiculata in a mangrove in Southern Thailandia. Aquat. Bot. 4: 43-52.

Cloern, J., Cole, B. & Wienke, S. Big Soda Lake (Nevada). 4. Vertical fluxes of particulate matter: seasonality and variations across thermocline. Limnol. Oceanogr. **32,** 815-824 (1987).

Clymo, R. S. The limits to peat bog growth. Phil. Trans. R. Soc. B. **303**, 605-654 (1984)

Coppock, D. L., Detling, J. K., Ellis, J. E. & Dyer, M. I. Plant-Herbivore Interactions in a North American Mixed-Grass Prairie. Oecologia **56**, 1-56 (1983)

Coveney, M. F., Cronberg, G., Enell, M., Larsoon, H. & Olofsson, L. Phytoplankton, zooplankton and bacteria-standing crop and production relationships in a eutrophic lake. Oikos **29**, 5-21 (1977).

Craft CB, Broome SW and Seneca ED. 1988. Soil nitrogen, phosphorus and organic carbon in transplanted estuarine marshes. In DD Hook, WH McKee, HK Smith, J Gregory, VG Burrell Jr. Mr. DeVoe, RE Sojka, S Gilbert, R Banks, LH Stolzy, C Brooks, TD Matthews and TH Shear (eds.). pp. 351-355 The Ecology and Managment of Wetlands. Timber Press Portland (OR).

Cushing, D. H. Grazing in Lake Erken. Limnol. Oceanogr. **21**, 349-356 (1976).

Cyr, H. and M. L. Pace. 1993. Magnitude and patterns of herbivory in aquatic and terrestrial ecosytems. Nature 361: 148-150.

Damman, A.W.H. 1978. Distribution and movement of elements in ombrotrophic peat bogs. Oikos 30: 480-495

Darley, W. M., Montague, C. L., Plumley, F. G., Sage, W. W. & Psalidas, A. T. Factors limiting edaphic algal biomass and productivity in a Georgia salt marsh. J. Phycol. 17, 122-128 (1981).

Davis SM (1991) Growth, decomposition and nutrient retention of Cladium jamaicense Crantz and Thypa dominguensis Pers. in the Florida everglades. Aquat. Bot. 40: 203-224.

Davis, M. and H. Lee. 1983. Recolonization of sediment-associated microalgae and effects of estuarine infauna on microalgal production. Mar. Ecol. Prog. Ser. 11: 227-232.

Davis, M. W., C. D. McIntire (1983). Effects of physical gradients on the production dynamics of sediments-associated algae. Mar. Ecol. Prog. Ser. 13: 103-114.

Day, F. P. Litter decomposition rates in the seasonally flooded great dismal swamp. Ecology **63**, 670-678 (1982).

Day, J., W. Smith, P. Wagner and W. Stone. 1973. Community structure and carbon budget of a salt marsh and shallow bay estuarine system in Lousiana. Center for Wetland Resources, Lousiana State University, Baton Rouge, Publ. NO. LSU-SG-72-04.

Day, T. A. and J. K. Detling. 1990. Grassland patch dynamics and herbivory grazing preference following urine deposition. Ecology 71: 180-188.

de la Cruz, A. A. The role of tidal marshes in the productivity of coastal waters. Assoc. Southeast. Biol. Bull. 20, 147-156 (1973)

DeAngelis, D. L., Gardner, R. H. & Shugart, H. H. in Dynamic Properties of Forest Ecosystems (ed Reichle, D. E.) 567-672 (Cambridge Unversity Press, New York, 1981).

Drew, E. A., and R. M. Hastings. 1992. A year-round ecophysiological study of *Himantothallus grandifolius* (Desmarestiales, Phaeophyta) at Sygny Island, Antarctica. Phycologia 31:262-277.

Dunton, K.H. and Schell, D.M. 1986. Seasonal carbon budget and growth *of L. solidungula* in the Alaskan high arctic. Mar. Ecol. Prog. Ser. 31: 57-66

Durbin, E.G., Krawiec, R.W. and Smaydan, T.J. 1975. Seasonal studies on the realtive importance of different size fractions of phytoplankton in Narragansett Bay, USA. Marine Biology 32:271-287.

Edroma, E. L. 1984. Effects of burning and grazing on the productivity and number of plants in Queen Elizabeth National Park, Uganda. African Journal of Ecology 22: 165-174.

Egan, B and Yarish, C. 1990. Productivity and life history of L. longicuris at its southern limit in the western atlantic ocean. Mar. Ecol. Prog. Ser. 67: 263-273

Emmerson, W. and L. McGwynne. 1992. Feeding and assimilation of mangrove leaves by the crab Sesarma meinerti de Man in relation to leaf-litter production in Myazana, a warm-temperate southern mangrove swamp. J. Exp. Mar. Biol. Ecol., 157: 41-53.

Erftemeier P., R. Osinga and M. Astrid. 1993. Primary production of seagrass beds in South Sulawesi (Indonesia): a comparison of habitats, methods and species. Aquat. Bot. 46: 67-90.

Fahey TJ, Stevens PA, Hornung M, Rowland P (1991) Decomposition and nutrient release from logging residue following conventional harvest of Sitka spruce in North Wales. Forestry 64: 289-301

Findlay, S, Howe K, Austin HK (1990) Comparison of detritus dynamics in two tidal freshwater wetlands. Ecology 71: 288-295.

Flint, R. W. & Goldman, C. R. The effects of a benthic grazer on the primary productivity of the littoral zone of Lake Tahoe. Limnol. Oceanogr. **20**, 935-945 (1975).

Flores Verdugo, F. J., J. W. Day, R. Briseno-Duenas (1987). Structure, litter fall, decomposition and detritus dynamics of mangroves in a Mexican coastal lagoon with an ephemeral inlet. Mar. Ecol. Prog. Ser. 35: 83-90.

Frangi, J. L. & Lugo, A. E. Ecosystem dynamics of a subtropical floodplain forest. Ecol. Monogr. **55**, 351-369 (1985).

Frank, D.A. and S.J. McNaughton (1992) The ecology of plants, large mammalian herbivores, and drought in Yellowstone National Park. Ecology 73(6):2043-2058

Frankignoulle, M., J. Bouquegneau, E. Ernst, R. Biondo, M. Rigo and D. Bay. 1984. Contribution de l´activité de l´Herbier de Posidonies au Metabolisme global de la Baie de Calvi. Premiers Resultats, p. 277-282. *In* C. Boudouresque, A. Jeudy de Grissac and J. Olivier [eds.], International Workshop Posidonia oceanica Beds (I) GIS Posidonie publ.

Frost, B. W. The role of grazing in nutrient-rich areas of the open sea. Limnol. Oceanogr. 36, 1616-1630 (1991)

Gabrielson JO, Birch PB, Hamel KS. (1983). Decompostion of Cladophora. II. In vitro studies of nitrogen and phosphorus regeneration. Bot Mar 26: 173-179

Gallagher, J.L., R.J. Reinhold, R.A. Linthurst and W.J. Pfeiffer. 1980. Aerial production, mortality and mineral accumulation-export dynamics in *S. alterniflora* and *J. roemerianus* plant stands in a georgia saltmarsh. Ecology 61: 303-312

Gandar, M. V. The dynamics and trophic ecology of grasshoppers (Acridoidea) in a South African Savanna. Oecologia **54**, 370-378 (1982).

Garber JH. 1984. Laboratory study of nitrogen and phosphorus remineralization during the decomposition of coastal plankton and seston. Estuarine Coastal Shelf Sci 18: 685-702

Garnier, J. & Mourelatos, S. Contribution of grazing in phytoplankton overall losses ina shallow French lake. Fresh. Biol. **25**, 515-523 (1991).

Gathuso, J., M. Pichon, B. Delesalle and M. Frankignoulle. 1993. Community metabolism and air-sea CO_2 fluxes in a coral reef ecosystem (Moore, French Polynesia). Mar. Ecol. Prog. Ser. 96: 259-267.

Geertz-Hansen, O., Sand-Jensen, K., Hansen, D.F. and Christiansen, A. 1993. Growth and grazing control of abundance of marine macroalgae, Ulva lactuca L. in an eutrophic Danish estuary. Aquat. Bot. 46: 101-109.

Gerard, V. 1976. Some aspects of material dynamics and energy flow in a kelp forest in Monterey Bay, California. Ph. D. Thesis, University of California, Santa Cruz.

Gliwicz, Z. M. & Hillbricht-Ilkowska. Efficency of the utilization of nannoplankton primary production by communities of filter feedng animals measured in situ. Verh. Internat. Verein. Limnol. **18**, 197-203 (1972).

Godshalk GL, Wetzel RG (1978a) Decomposition of aquatic angiosperms. II Particulate components. Aquat. Bot. 5: 301-327.

Godshalk GL, Wetzel RG (1978b). Decomposition of aquatic angiosperms. III Zostera marina L. and a conceptual model of decomposition. Aquat. Bot. 5: 329-354.

Golley, F. B. 1960. Energy dynamics of a food chain of an old-field community. Ecol. Monogr. 30: 187-206.

Golley, F., H. Odum and R. Wilson. 1962. The structure and metabolism of a Puerto Rican Red Mangrove Forest in May. Ecology 43: 8-19.

Golterman HL (1972) The role of phytoplankton in detritus formation. Mem Ist Ital Idrobiol 29: 89-103

Gosz, J. R., Holmes, R. T., Likens, G. E. & Bormann, F. H. The flow of Energy in a Forest Ecosystem. Sci. Am. **238**, 93-102 (1978).

Gould, D. and E. Gallagher. 1990. Field measurement of specific growth rate, biomass and primary production of benthic diatoms of Savin Hill Cove, Boston. Limnol. Oceanogr. 35:1757-1770.

Graneli, W. (1989). Influence of standing litter on shoot production in reed, Phragmites australis. Aquat. Bot. 35: 99-109.

Gray, J. T. Community structure and productivity in Ceanothus chaparral and coastal sage scrub of southern California. Ecol. Monogr. **52**, 415-435 (1982).

Greenway, M. The grazing of Thalassia testudinum in Kingston Harbour, Jamaica. Aquat. Bot. 2, 117-126 (1976).

Grier, C. C. & Logan, R. S. Old-growth Pseudotsuga menziesii communities of a western Oregon watershed: biomass distributon and production budgets. Ecol. Monogr. **47**, 373-400 (1977).

Grodzinski, W., A. Gorecki, K. Janas and P. Migula. 1966. Effect of rodents on the primary productivity of alpine meadows in Bierzezady mountains. Acta Theriol. 11: 419-431.

Gulati, R. D. A study on the role of herbivorous zooplankton community as primary consumers of phytoplankton in Dutch lakes. Verh. Internat. Verein. Limnol. **19**, 1202-1210 (1975).

Haines EB, Hanson RB (1979) Experimental degradation of detritus made from the salt marsh plants Spartina alterniflora Loisel, Salicornia virginica L. and Juncus roemerianus Scheele. J Exp mar Biol Ecol 40: 27-40

Hanisak, M.D. 1979. Nitrogen limitation of *C. Fragile* ssp. *tomentosoides* as determined by tissue analysis. Marine Biology 50: 333-337

Hansen, J. A., D. Klumpp, D. Alongi, P. Dayton and M. Riddle. 1992. Detrital pathways in a coral reef lagoon. II. Detritus deposition, benthic microbial biomass and production. Mar. Biol. 113: 363-372.

Harris, W. F., Sollins, P., Edwards, N. T., Dinger, B. E. & Shugart, H. H. in Productivity of the world systems. Proceedings of a Symposium (eds Reichle, D., Franklin, J. & Goodall, D.) 116-122 (National Academy of Sciences, Washington, D.C, 1975).

Harrison PG (1982) Control of microbial growth and of amphipod grazing by water soluble compounds from leaves of Zostera marina. Mar Biol 67: 225-230

Harrison PG (1989) Detrital processing in seagrass systems: a review of factors affecting decay rates, remineralization and detritivory. Aquat Bot 23: 263-288

Harrison, W. G., E. Head, E. Horne, B.Irwin, W. Li, A. Longhurst, M. Paranjape and T. Platt. 1993 The Western North Atlantic Bloom Experiment. Deep-Sea Res. 40: 279-305.

Hatcher, B.G., S. V. Smith and C. Crossland. 1992. The role of Coral Reefs in Global Ocean Production, p. 513-514.*In* P. Falkowski and A.Woodhead [eds.], Primary Productivity and Biogeochemical Cycles in the Sea. Plenum Press.

Haukioja, E. and Koponen S. 1975. Birch herbivores and herbivory at Kevo. In Fennoscandian Tundra Ecosystems. Part 2. Animals and System Analyses (ed. Wielgolaski, F. E.) 181-188 (Springer, Heidelber).

Hauxwell J, McClelland J, Behr PJ, Valiela I. 1998. Relative importance of grazing and nutrient controls of macroalgal biomass in three temperate shallow estuaries. Estuaries 21: 347-360

Hauxwell, J, J. Cebrian and I. Valiela. 2002. Annual growth dynamics of eelgrass (Zostera marina L.) in estuaries of Waquoit Bay, MA, USA: Relationship to land-derived nitrogen loads and effect on light supply for eelgrass. Mar. Ecol. Prog. Ser. (in press).

Heal, O. W. in Tundra Biome. Proceedings IV International Meeting on the Biological Productivity of Tundra (eds Wielgolaski, F. & Rosswall, T.) 295-305 (Tundra Biome Steering Committee, Stockholm, 1972).

Hemmings MA, Buth GJC (1991) Decomposition in salt marsh ecosystems of the SW Netherlands: the effects of biotic and abiotic factors. Vegetatio 92: 73-83

Hemmings MA, Nieuwenhuize J (1991) Transport, deposition and "in situ" decay of seagrasses in a tropical mudflat area (Banc d'Arguin, Mauritania). Neth J Sea Res 27: 183-190

Hill BH (1979) Uptake and release of nutrients by aquatic macrophytes. Aquat. Bot. 7: 87-93.

Hodges, C., T.Thompson, J. Riley and E. Glenn. 1993. Reversing the flow: water and nutrients from the sea to the land. Ambio 22: 483-490.

Holligan, P.M., P. J. leB. Williams, D. Purdie and R. Harris. 1984. Photosynthesis, respiration and nitrogen supply of plankton populations in stratified, frontal and tidally mixed shelf waters. Mar. Ecol Progr. Ser. 17:201-213.

Holligan P., E. Fernández, J. Aiken, W. Balch, P. Boyd, P. Burkill, M. Finch, S. Groom, G. Malin, K. Muller, D. Purdie, C. Robinson, C. Trees, S. Turner and P. van der Wal. 1993. A Biogeochemical Study of the Coccolithophore, Emiliania huxleyi, in the north Atlantic. Global Biogeochemi. Cycles 7: 879-900.

Hopkinson, O., J. Gosselink and R. Panardo. 1978. Aboveground production of seven marsh plant species in coastal Lousiana. Ecology 59: 760-769.

Howes, B., J. Dacey and J. Teal. 1985. Annual C mineralization and belowground production of Spartina alterniflora in a new England salt marsh. Ecology 66: 595-605.

Hunter, R. D. Effects of grazing on the quantity and quality of freshwater aufwuchs. Hydrobiologia **69**, 251-259 (1980).

Iriarte, A., G. Daneri, V. Garcia, D. Purdie and D. Crawford. 1991 Plankton community respiration and its relationship to Cha concentration in marine coastal waters. Oceanologia Acta 14:379-387.

Jacobs, R., den Hartog, C., Braster, C. & Carrière, F. Grazing of the seagrass Zostera noltii by birds at Terschelling (Dutch Wadden Sea). Aquat. Bot. 10, 241-259 (1981).

Jacoby, J. M. Alterations in periphyton characteristics due to grazing in a Cascade foothill stream. Fresh. Biol. **18**, 495-508 (1987).

Jensen, L. M. & Schwaerter, S. Major pathways involved in the utilization of primary production in a temperate eutrophic lake. Verh. Internat. Verein. Limnol. **23**, 445-450 (1988).

Jensen, L. M., K. Sand-Jensen, S. Marcher and M. Hansen. 1990. Plankton community respiration along a nutrient gradient in a shallow Danish Estuary. Mar. Ecol. Prog. Ser. 61:75-85

Jespersen, A. M., Christoffersen, K. & Riemann, B. Annual carbon fluxes between phyto-, zoo-, and bacterio-plankton in eutrophic, Lake Frederiksborg Slotssø, Denmark. Verh. Internat. Verein. Limnol. **23**, 440-444 (1988).

Johnson, F.L. and P.G. Rissor. 1974. Biomass, annual net primary production and dynamics of six mineral elements in a post oak-blackjack oak forest. Ecology. 55: 1246-1258

Johnson, K. M., C. M. Burney and J. Sieburth. 1981. Enigmatic marine ecosystem metabolism measured by direct diel $\sum CO_2$ and O_2 flux in conjunction with DOC release and uptake. Mar. Biol. 65:49-60.

Johnstone, I. M. (1981) Consumption of leaves by herbivores in mixed mangrove stands. Biotropica 13: 252-259.

Joiris, C., G. Billen, C. Lancelot , M. Daro, J. Mommaerts, A. Bertels, M. Bossicart and J. Nijs. 1982. A budget of Carbon Cycling in the Belgian Coastal Zone:Relative roles of zooplankton, bacterioplankton and benthos in the utilization of Primary Production. Neth. J. Sea Res. 16:260-275.

Josselyn M. N., A. C. Mathieson (1980). Seasonal influx and decomposition of autochthonous macrophyte litter in a north temperate estuary. Hydrobiologia 71: 197-208.

Josselyn, M., M. Fonseca, T. Niesen and R. Larson. 1986. Biomass, production and decomposition of a deep water seagrass, Halophila decipiens Ostenf. Aquat. Bot. 25: 47-61.

Jupp, B. P. & Spence, D. H. N. Limitations of macrophytes in a eutrophic lake, Loch Leven. II Wave action, sediments and waterfwol grazing. J. Ecol. **65**, 431-446 (1977).

Kamiyama, T. 1994. The impact of grazing by microzooplankton in northern Hiroshima Bay, the Seto Island Sea, Japan. Mar. Bio. 119:77-88.

Karagatzides, J. D. & Hutchinson, I. Intra-specific comparisons of biomass dynamics in Scirpus americanus and Scirpus maritimus on the River Delta. J. Ecol. **79**, 459-476 (1991).

Kemp, W. M. and W. R. Boynton. 1980. Influence of biological and physical processes on dissolved oxygen dynamics in an Estuarine System:implication for measurement of community metabolism. Estuar. Coast. Mar. Sci. 2:407-431.

Kenney, B., W. Litaker, C. Duke and J. Ramus. 1988. Community oxygen metabolism in a shallow tidal estuary. Estuar. Coast. Shelf Sci. 27:33-34.

Kenworthy, J. and G. Thayer. 1984. Production and decomposition of the roots and rhizomes of seagrasses, Zostera marina and Thalassia testudinum, in temperate and subtropical marine ecosystems. Bull. Mar. Sci. 35: 364-379.

Khailov, K. M. & Burlakova, Z. P. Release of dissolved organic matter by marine seaweeds and distribution of their total organic production to inshore communities. Limnol. Oceanogr. 14, 521-527 (1969).

Kiene, W. and P. Hutchings. 1994. Bioerosion experiments at Lizard Island,Great Barrier Reef. Coral Reefs 13:91-98.

King, F., A. Devol and T. Packard. 1978. Plankton metabolic activity in the eastern tropical North Pacific. Deep-Sea Res. 25: 689-704.

Kira, T. in Tropical trees as living systems (eds Tomlinson, P. B. & Zimmerman, M. H.) 561-590 (Cambridge University Press, New York, 1978)

Kirkman, D. & Reid, D. A study of the role of the seagrass Posidonia australis in the carbon budget of an estuary. Aquat. Bot. 7, 173-183 (1979).

Klopatek, J. & Stearns, F. Primary productivity of Emergent Macrophytes in a Wisconsin freshwater marsh ecosystem. Am. Mid. Nat. **100**, 320-332 (1978).

Klumpp D. and N. Polunin. 1989. Partioning among grazers of food resources within damselfish territories on a coral reef. J. Exp. Mar. Ecol. Biol. 125: 145-169.

Klumpp, D., R. Howard, D. Pollard. 1989. Trophodynamics and nutritional ecology of seagrass communities, p. 349-475.*In* A. Larkum, A. McComb and S. Sepherd S. [eds], Biology of Seagrasses: a Treatise on the Biology of Seagrasses with Special Reference to the Australian Region. Elvesier.

Klumpp, D. W., Salita-Espinosa, J. S. & Fortes, M. D. The role of epiphytes and marcoinvertabrate grazers in the trophic flux of a tropical seagrass community. Aquat. Bot. 43, 327-349 (1992)

Klumpp, D. W., Salita-Espinosa, J. S. & Fortes, M. D. Feeding ecology and trophic role of sea urchins in a tropical seagrass community. Aquat. Bot. 45, 205-229 (1993).

Knauer, G.A., Martin, J.H. and Bruleud, K.W. 1979. Fluxes of particulate carbon, nitrogen and phosphorus in the upper water column of the northeast Pacific. Deep-sea Research. 26: 97-108

Koop, K., R. Newell and M. Lucas. 1982. Biodegradation and carbon flow based on kelp (Ecklonia maxima) debris in a Sandy Beach Microcosm. Mar. Ecol. Prog. Ser. 7:315-326.

Kristensen, E. 1993. Seasonal variations in Benthic Community Metabolism and nutrient dynamics in a Shallow, Organic-poor danish lagoon. Estuar. Coast. Shelf Sci. 36:565-586.

Kristensen, E., F. Andersen and T. Blackburn. 1992. Effects of benthic macrofaune and temperate on degradation of macroalgal detritus: the fate of organic carbon. Limnol. Oceanogr. 37: 1404-1419.

Kruse, B. 1993. Measurement of plankton O_2 respiration in gas-tight plastic jars. Mar. Ecol. Prog. Ser. 94:155-163.

Kuparinen, J. 1986. Production and respiration for overall plankton and ultraplankton communities at the entrance to the Gulf of Finland in the Baltic Sea. Mar. Biol. 63:591-607.

Lampert, W. Climatic conditionss and planktonic interactions as factors controlling the regular succession of spring algal bloom and extremely clear water in Lake Constance. Verh. Internat. Verein. Limnol. **20**, 969-974 (1978).

Lapointe, B.E., Niell, F. and Fuentes, J.M. 1981. Communtiy structure, succession and production of seaweeds associated with mussel-beds in the Ria de Arosa, NW Spain. Mar. Ecol. Prog. Ser. 5: 243-253

Lawes, E., Bienfang, P., Ziemann, D. &Conquest, L. Phytoplankton population dynamics and the fate of production during the spring bloom in Auke Bay, Alaska. Limnol. Oceanogr. 33, 57-65 (1988).

LeBorgne, R., Blanchot, J. and L. Charpy. 1989. Zooplankton of Tikehau atoll (Thamoth archipielago) and its relationship to particulate matter. Mar. Biol. 102: 341-353.

LeBorgne, R., M. Rodier, A. LeBouteiller, and M. Kulbicki. 1997. Plankton biomass and production in an open atoll lagoon: Uvea, New Caledonia. J. Exp. Mar. Biol. Ecol. 212:187-210.

Lee, S. 1989. The importance of sesarminae crabs Chiromanthes sp. and inundation frequency on mangrove (Kandelia Kandel (L.) Durce) leaf litter turnover in a Hong Kong Tidal shrimpond. J. Exp. Mar. Biol. Ecol. 1989. 131: 23-43.

Lee, S. 1990. Net aerial primary productivity, litter production and decomposition of the reed Phragmites communis in a nature reserve in Hong-Kong: management implications. Mar. Ecol. Prog. Ser. 66:161-173.

Leigh, E. G. & Windsor, D. M. in Ecology of a tropical forest:seasonal rhythms and long term changes 111-123 (eds Leigh, E. G., Raud, A. & Windsor, D. M. (Smithsonian Institution Press, Washington, 1983).

Lenz, J., A. Morales and J. Gunkel. 1993 Mesozooplankton standing stock during the North Atlantic spring bloom study in 1989 and its potential grazing pressure on phytoplankton: a comparison between low, medium and high latitudes. Deep-Sea Res. (II) 40: 559-572.

Li M. S., S. Y. Lee (1998). Carbon dynamics of Deep Bay, eastern Pearl River Estuary, China. I: A mass balance budget and implications for shorebird conservation. Mar. Ecol. Prog. Ser. 172: 73-87.

Lignell, R., Heiskanen, A. S., Kuosa, H., Gundersen, K., Kuuppo-Leinikki, P., Pajuniemi, R. & Uitto, A. Fate of phytoplankton spring bloom: sedimentation and carbon flow in the planktonic food web in the northern Baltic. Mar. Ecol. Prog. Ser. 94, 239-252 (1993).

Lindeboom, H. and B. De Bree. 1982. Daily production and consumption in a eelgrass (Zostera marina) community in Saline-Lake Grevelingen: discrepancies between the O_2 and ^{14}C methods. Neth. J. Sea Res. 16:362-379.

Lindeboom, H. and A. Sandee. 1989. Production and consumption of tropical seagrass fields in eastern Indonesia measured with bell jars and microelectrods. Neth. J. Sea Res. 23:181-190.

Lindeboom H., W. Raaphorst, H. Ridderinkhof and H. W. van der Veer. 1989. Ecosystem model of the Western Wedden Sea: a bridge between science and management. Helgolander Meeresunters 43: 549-564.

Lock, J. M. 1972. The effects of hippopotamus grazing on grasslands. J. Ecol. 60: 445-467.

Lowman, M. D. Litterfall and leaf decay in three australian rainforest formations. J. Ecol. **76**, 451-465 (1988).

Mann, K. H. 1972. Ecological energetics of the sea-weed zone in a marine bay on the Atlantic coast of Canada. II. Productivity of seaweeds. Mar. Biol. 14: 199-209.

Mann, K.H. 1985. Ecology of Coastal Waters. Studies in Ecology Volume 8. 2nd ed. Blacwell.

Mann, K.H. 1988. Production and use and detritus in various freshwater, estuarine and coastal marine ecosystems. Limnol. Oceanogr. 33: 910-931.

Mason, C. F. & Bryant, R. J. Periphyton production and grazing by chironomids in Alderfen Broad, Norfolk. Freshwat. Biol. **5**, 271-277 (1975).

McClaugherty CA, Pastor J, Aber JD (1985) Forest litter decomposition in relation to soil nitrogen dynamics and litter quality. Ecology 66: 266-275

McInnes P. F., R. J. Naiman, J. Pastor and Y. Cohen. 1992. Effects of moose browsing on vegetation and litter of the boreal forest, Isle Royale, Michigan, USA. Ecology 73: 2059-2075.

McLachlan, A. &Bate, G. Carbon budget for a high energy surf zone. Vie milieu 34, 67-77 (1984).

McLusky, DS. The estuarine ecosystem (Wiley, New York, 1981).

McNaughton, S. J. Ecology of a grazing ecosystem: The Serengeti. Ecol. Monogr. **55**, 259-294 (1985).

McNaughton, S. J., M. Oesterheld, D. A. Frank and K. J. Williams. 1989. Ecosystem-level patterns of primary productivity and herbivory in terrestrial habitats. Nature 341: 142-144.

McQuaid, C. D and G. M. Branch. 1985. Trophic structure of rocky intertidal communities: response to wave action and implications for energy flow. Mar. Ecol. Prog. Ser. 22: 153-161.

Mee, L. D. 1977. The Chemistry and Hydrography of SomeTropical Coastal Lagoons-Pacific Coast of Mexico. Ph. D. Tesis. University of Liverpool. Dept. of Oceanography, England.

Megonigal, J. P. & Day Jr., F. P. Organic matter dynamics in four seasonally flooded forest communities of the dismal swamp. Amer. J. Bot. **75**, 1334-1343 (1988).

Menhinick, E. F. Structure, Stability and Energy flow in plants and arthropods in a Sericea lespedeza stand. Ecol. Monogr. **37**, 256-272 (1967)..

Menzel, D. and J. Ryther. 1960 The annual cycle of primary production in the Sargasso Sea off Bermuda. Deep Sea Res. 6:351-357.

Merino, M., A. González, E. Reyes, M. Gallegos and S. Czitrom. 1992. Eutrophication in the lagoons of Cancún, México. Science of the Total Environment, Supplement (1992): 861-870.

Miller, P. C., Miller. P. M., Blake-Jacobson, M., Chapin, F. S., III, Everett, K. R., Hilbert, D. H., Kummerow, J., Linkins, A. E., Marion, G. M., Oechel, W. C., Roberts, S. W. & Stuart, L. Plant-soil processes in Eriophorum vaginatum tussock tundra in Alaska: a systems modeling approach. Ecol. Monogr. **54**, 361-405 (1984).

Mitchell DT, Coley PGF, Webb S, Allsopp N (1986) Litterfall and decomposition processes in the coastal fynbos vegetation, South-Western Cape, South Africa. J. Ecol. 74: 977-993.

Montagna, P. A. In situ measurement of meiobenthic grazing rates on sediment bacteria and edaphic diatoms. Mar. Ecol. Progr. Ser. 18, 119-130 (1984).

Moore, T. R. Growth and net production of Sphagnum at five fen sites, subartic eastern Canada. Can. J. Bot. **67**, 1203-1207 (1989)

Müller, P. J. & Suess, E. Productivity, sedimentation rate, and sedimentary organic matter in oceans- I. Organic carbon preservation. Deep Sea Research 26, 1347-1362 (1979).

Murray, L. and R.L. Wetzel. 1987. Oxygen production and consumption associated with the major autotrophic components in two temperate seagrass communities. Mar. Ecol. Progr. Ser. 38: 231-239.

Naiman, R. J. Primary production, standing stock and export of organic matter in a Mohave Desert thermal stream. Limnol. Oceanogr. **21**, 60-73 (1976).

Nakamori, T., A. Suzuki, Y. Iryu, Cont. Shelf Res. 12, 951 (1992).

Nakanishi, M., Y. Tezuka, T. Narita, O. Mitamura, K. Kawabata and S. Nakano. 1992. Phytoplankton primary production and its fate in a pelagic area of Lake Biwa. Arch. Hydrobiol. Beih. Ergebn. Limnol. 35: 47-67.

Nedwell, D. B., Walker, T. R., Ellis-Evans, J. C. & Clarke, A. Measurements of Seasonal Rates and Annual Budgets of Organic Carbon fluxes in an Antarctic Coastal Environment at Signy Island. Appl. Environ. Microbiol. 59, 3989-3995 (1993)

Neely RK, Davis CB (1985) Nitrogen and phosphorus fertilization of Sparganium eurycarpum Engelm. And Typha glauca Godr. Stands. II. Emergent plant decomposition. Aquat. Bot. 22: 363-375.

Neill, C. 1992. Life history and popualtion dynamics of whitetop shoots under dufferent levels of flooding and nitrogen supply. Aquatic. Bot. 42: 241-252.

Nelson, W. J., J. A. Kadlec and H. R. Murkin. 1990. Seasonal comparison of weight for two types of Typha glauca Godr. leaf litter. Aquat. Bot. 37: 299-314.

Nessel J. 1978. Distribution and dynamics of organica matter and phosphorus in a sewage enriched cypress stand. MS thesis, Univ. of Florida, Gainesville, Fla, 159 pp.

Neudecker, S. 1979. Effects of grazing and browsing fishes on the zonation of Corals in Guam. Ecology 60: 666-672.

Newell RC, Lucas MI, Linley EAS (1981) Rate of degradation and efficiency of conversion of phytoplankton debris by marine micro-organisms. Mar Ecol Prog Ser 6: 123-136

Newell, R., J. Field and C.Griffiths. 1982. Energy Balance and significance of microorganisms in a kelp bed community. Mar. Ecol. Prog. Ser. 8:103-113.

Newell SY, Fell JW, Statzell-Tallman A, Miller C, Cefalu R (1984). Carbon and nitogen dynamics in decomposing leaves of three coastal marine vascular plants of the subtropics. Aquat Bot 19: 183-192

Newell SY, Fell JW, Miller C (1986) Deposition and decomposition of turtlegrass leaves. Int Rev Ges Hydrobiol 71: 363-369

Nicotri, M. E. Grazing effects of four marine intertidal herbivores on the microflora. Ecology 58, 1020-1032 (1977)

Nielsen. Aboveground food resources and herbivory in a beech forest ecosystem. Oikos 31: 273-279 (1978).

Nienhuis, P. & Groenendijk, A. Consumption of eelgrass (Zostera marina) by birds and invertebrates: an annual budget. Mar. Ecol. Prog. Ser. 29, 29-35 (1986).

Nihlgard. 1972. Plant biomass, primary production and distribution of chemical elements in a beech and planted spruce forest in southern Sweden. Oikos 23: 69-81

Nixon S. and C. Oviatt. 1972. Preliminary measurements of midsummer metabolism in beds of eelgrass, Zostera marina. Ecology 53:150-153.

Nixon, S. and M. E. Pilson. 1984. Estuarine total system metabolism and organic exchange calculated from nutrient ratios: an example from Narragansset Bay, p. 261-291. *In* V. S Kennedy [ed.],The Estuary as a filter. Academic Press.

O'Connell AM (1987) Litter dynamics in Karri (Eucalyptus diversicolor) forest of South-Western Australia. J. Ecol. 75: 781-796.

O'Connell AM (1988) Nutrient dynamics in decomposing litter in karri (Eucalyptus diversicolor F. Muell) forests of South-Western Australia. J. Ecol. 76: 1186-1203.

Odum, E. P. (1971). Fundamentals of Ecology. 3rd ed. Saunders. Philadelphia, London & Toronto. pp. XIV +574.

Odum, E. P., C. E. Connell and L. B. Davenport. 1962. Population energy flow of three primary consumer components of old-field ecosystems. Ecology 43: 88-96.

Odum, H. T. Trophic structure and productivity of Silver Springs, Florida. Ecol. Monogr. **27**, 55-112 (1957).

Odum, H. T. and R. F. Wilson 1962. Further studies on reareation and metabolism of Texas bays, 1958-1960. Publ. Inst. Mar. Sci. Univ. Tex., 8:20-55.

Oechel, W. & Billings, W. D. in Arctic Ecosystems in a changing climate. An Ecophysiological perspective. (eds. Chapin III, F. Stuart, Jefferies, R. L., Reynolds, J. F., Shaver, G. R. & Svoboda, J.) 139-168 (Academic Press, New York, 1992).

Ohmart, C. P., Stewart, L. G. & Thomas, J. R. Leaf consumption by insects in three Eucalyptus forest types in Southeastern Australia and their role in short-term nutrient cycling. Oecologia **59**, 322-330 (1983).

Olson, J. S. in Analysis of temperate forest ecosystems. Ecological studies 1 (ed. Reichle, D. E.), 226-246 (Springer-Verlag, New York, 1970).

Omuf, C., J. Teal and I. Valiela. 1977. Interactions of nutrients, plant growth and herbivory in a mangrove ecosystem. Ecology 58:514-526.

Ong, J. E. (1993). Mangroves-a carbon source and sink. Chemosphere 27: 1097-1107.

Ong JE, Gong WK, Wong CH. 1982. Studies on nutrient levels in standing biomass, litter and slash in a mangrove forest. Univ. Sains Malaysia, Penang, Sch. Biol. Sci., 44 p.

Østergaad, F. and E. Kristensen. 1992. The importance of benthic macrofauna in decomposition of microalgae in a coastal marine sediment. Limnol. Oceanogr. 37:1392-1403.

Otsuki A and Hanya T. 1972. Production of dissolved organic matter from dead green algal cells. I Aerobic microbial decompostion. Limnol. Ocanogr 17: 248-257

Ott, J. and L. Maurer. 1977. Strategies of energy transfer from marine macrophytes to consumer levels: the Posidonia Oceanica example, p. 493-502.*In* B. Keegan [ed.], Biology of Benthic Organisms. Pergamon Press.

Packard, T. T. 1979. Respiration and respiratory electron transport activity in plankton for the Northwest African upwelling area. J. Mar. Res. 37: 712-739.

Packard, T. T. and P. J. le B Williams. 1981. Rates of respiratory oxygen consumption and electron transport in surface seawater from the northwest Atlantic. Oceanologia acta 4:351-358.

Pakarinen, P. 1978. Production and nutrient ecology of three Sphagnum species in southern Finnish raised bogs. Ann. Bot. Fennia 15: 15-26

Palm CA, Sanchez PA (1990) Decomposition and nutrient release patterns of the leaves of three tropical legumes. Biotropica 22: 330-338

Panatmat, M. 1988. Ecology and metabolism of a benthic community on an intertidal sandflat. Int. Revue. ges. Hydrobiol. 53: 211-298.

Pandey, C. B. and J. S. Singh. 1992. Rainfall and grazing effects on net primary productivity in a tropical savanna, India. Ecology 73: 2007-2021.

Parson, K.A. and A.A. de la Cruz (1980) Energy flow and grazing behavior of conocephaline grasshoppers in a *Juncus roemerianus* marsh. Ecology 61(5):1045-1050

Patriquin DG and McClung CR. 1978. Nitrogen acretion and the nature and possible significance of N_2 fixation (acetylene reduction) in a Nova Scotia Spartina alterniflora stand. Mar Biol 47: 227-242

Pattersen, M., K. Sebans and R. Olsen. 1991. In situ measurements of flow effects on primary production and dark respiration in reef corals. Limnol. Oceanogr. 36:936-948.

Peckol, P. B., J. S. Rivers (1996) Contribution by macroalgal mats to primary production of a shallow embayment under high and low nitrogen-loading rates. Estuar. Coast. Shelf Sci. 43: 311-325.

Pedersen, M. F. and J. Borum. 1996. Nutrient control of algal growth in estuarine waters. Nutrient limitation and the importance of nitrogen requirements and nitrogen storage among phytoplankton and species of macroalgae. Mar. Ecol. Prog. Ser. 142: 261-272.

Peduzzi P, Herndl GJ (1991) Decomposition and significance of seagrass leaf litter (Cymodocea nodosa) for the microbial food web in coastal waters (Gulf of Trieste, Northern Adriatic Sea). Mar Ecol Prog Ser 71: 163-174

Pellikaan, G. and P. Nienhuis. 1988. Nutrient uptake and release during growth and decomposition of eelgrass, Zostera marina L., and its effect on the nutrient dynamics of Lake Grevelingen. Aquat. Bot. 30:189-214.

Pergent, G., J. Romero, C. Pergent-Martini, M. Mateo and C. Boudouresque. 1994. Primary production, stocks and fluxes in the Mediterranean seagrass P. oceanica. Mar. Ecol. Prog. Ser.106: 39-146.

Perry, M.J. 1976. Phospahte utilization by an oceanic diatom in phosphorus-limited chemostat culture and in the oligotrophic waters of the central North Pacific. Limnology and Oceanography 21: 88-107

Polunin, N. and D. Klumpp. 1992. Algal food suply and grazer demand in a very productive coral-reef zone. J. Exp. Mar. Biol. Ecol. 164:1-16.

Pomeroy, L. R. and J. Johannes. 1968. Ocurrence and respiration of ultraplankton in the upper 500 m of the ocean. Deep Sea Res. 15: 381-391.

Poumba, K. 1994. Simulated degradation of phytodetritus in deep-sea sediments of the NE Atlantic (47ºN, 19ºW). Mar. Ecol. Progr. Ser. 105:291-299.

Prego, R. General aspects of carbon biogeochemistry in the ria of Vigo, northwestern Spain. Geochim. cosmochim. Acta 57, 2041-2052 (1993).

Proctor, J., Anderson, J. M., Fogden, S. C. L. & Vallack, H. W. Ecological Studies in Four Contrasting Lowland Rain Forests in Gunung Mulu National Park, Sarawak. J. Ecol. **71**, 261-283 (1983).

Qasim, S and P. Bhattathiri. 1971. Primary production of a seagrass bed on Kavarotti Atoll (Laccavides). Hydrobiologia 38: 29-38.

Randall, J. and J. W. Day. 1987. Effects of River Dicharge and vertical circulation in aquatic primary production in a turbid Lousiana (USA) Estuary. Neth. J. Sea Res. 21: 231-242.

Reader, R.J. and J.M. Stewart (1972) The relationship between net primary production and accumulation for a peatland in southeastern Manitoba. Ecology 53(6):1024-1037

Reddy KR, DeBusk WF (1991) Decomposition of water hyacinth detritus in eutrophic lake water. Hydrobiologia 211: 101-109.

Reichardt, W. and G. Dieckmann. In Antarctic Nutrient Cycles and Food Webs (eds. Siegfried, W. R., P. R. Condy and R. M.Laws) 115-122 (4th SCAR Symposium on Antarctic Biology, Springer-Verlag, New York, 1985).

Reichle, D. E. & Crossley Jr. D. A. in Secondary Productivity of Terrestrial Ecosystems (ed Petrusewicz, K.) 563-581 (U. S Atomic Energy Commission, Warszawa, 1967).

Reichle DE, Goldstein RA, Van Hook RI, Dodson GJ. 1973. Analysis of insect consumption in a forest canopy. Ecology 54: 1076-1084

Richardson, C.J., G.M. Ferrell, and P. Vaithiyanathan (1999) Nutrient effects on stand structure, resorption efficiency, and secondary compounds in everglades sawgrass. Ecology 80(7):2182-2192

Ritchie, M. E., D. Tilman and J. M. H. Knops. 1998. Herbivore effects on plant and nitrogen dynamics in Oak Savanna. Ecology 79: 165-177.

Rizzo, W. and R. Wetzel. 1985. Intertidal and shoal benthic community metabolism in a temperate estuary: studies of spatial and temporal scales of variability. Estuaries 8: 342-351.

Robertson, A. and P. Daniel. 1989. Decomposition and the annual flux of detritus from fallen timber in tropical mangrove forests. Limnol. Oceanogr. 34: 640-647.

Rogers KH, Breen CM (1982) Decomposition of Potamogeton crispus L: The effects of drying on the pattern of mass and nutrient loss. Aquat. Bot. 12: 1-12.

Romero, J., Pergent, G., Pergent-Martini, C., Mateo, M. A. & Regnier, C.The detritic compartment in a Posidonia oceanica meadow: litter features, decomposition rates and mineral stocks. P. S. Z. N. I. Mar. Ecol. 13, 69-83 (1992).

Rublee PA, Roman MR (1982) Decomposition of turtlegrass (Thalassia testudinum Koning) in flowing sea-water tanks and litterbags: compositional changes and comparisons with natural particulate matter. J Exp Mar Biol Ecol 58: 47-58

Sakshaug, E., A. Bjorge, B. Gulliksen, H. Loeng, and F. Mehlum. 1994. Structure, biomass, distribution, and energetics of the pelagic ecosystem in the Barents Sea: A synopsis. Polar Biol. 14:405-411.

Schalles, J. F. & Shure, D. J. Hydrology, community structure, and productivity patterns of a dystrophic Carolina bay Wetland. Ecol. Monogr. **59**, 365-385 (1989)

Schlesinger, W.H. 1978. Community structure, dynamics and nutrient cycling in the Okefenokee cypress swamp forest. Ecol. Mono. 48: 43-65

Schlesinger, W. H. Biogeochemistry: an analysis of global change, 2nd ed. (Academic Press, San Diego, CA, 1997)

Schowalter, T. D., J. W. Webb and D. A. Crossley Jr. 1981. Community structure and nutrient content of canopy arthropods in clearcut and uncut forest ecosystems. Ecology 62: 1010-1019.

Scott, J.A., N.R. French, and J.W. Leethom (1979) Patterns of consumption in grasslands. In: Perspectives in grassland ecology. [Ed.] French N.R., New York: Springer-Verlag, 89-105 *Cited in Pomeroy, L.R. and R.G. Wiegert (1981) The Ecology on a Salt Marsh. New York, Springer-Verlag, pp 271*

Seastedt TR (1988) Mass, nitrogen and phosphorus dynamics in foliage and root detritus of tallgrass prairie. Ecology 69: 59-65

Sfriso A., A. Marcomini (1997). Macrophyte production in a shallow coastal lagoon. Part I: coupling with chemico-physical parameters and nutrients concentrations in waters. Mar. Environ. Res. 44: 351-375

Sharma E, Ambasht RS (1987) Litterfall, decomposition and nutrient release in an age sequence of Alnus nepalensis plantation stands in the eastern Himalaya. J. Ecol. 75: 997-1010

Sheldon, S. P. The effects of herbivorous snails on submerged macrophyte communities in Minnesota lakes. Ecology **68**, 1920-1931 (1987).

Shin CN, Rhee GY and Chen J. 1987. Phosphate requirement, photosynthesis and diel cell cycle of Scenedesmus obliquus under fluctuating light. Can J Fish Aquat Sci 44: 1753-1758

Sinclair, A. R. E. The resource limitation of trophic levels in tropical grassland ecosystems. J. Anim. Ecol. **44**, 497-520 (1975).

Skjoldal, H. R. & Lännergren, C. The Spring Phytoplankton Bloom inLindåspollene, a Land-locked Norwegian Fjord. II. Biomass and Activity of Net- and Nanoplankton. Mar. Biol. 47, 313-323 (1978).

Smalley, A. E. 1960. Energy flow of a salt marsh grasshopper population. Ecology 41: 672-677.

Smith, K. L. Jr. 1992. Benthic Boundary Layer Communities and carbon cycling at abyssal depths in the Central North Pacific. Limnol. Oceanogr. 37:1034-1057.

Smith, L. & Clymo, R. S. An extraordinary peat-forming peat-forming community on the Falkland Islands. Nature **309**, 617-620 (1984).

Smith, R. E., W. Harrison, B. Irwin and T. Platt. 1986. Metabolism and carbon exchange in microplankton of the Grand Banks (Newfouland). Mar. Ecol. Prog. Ser. 34: 171-183.

Smith, S.V. 1973. Carbon dioxide dynamics: a record of organic carbon production, respiration and calcification in the Eniwetok reef flat community. Limnol. Oceanogr. 18: 106-120.

Smith, S. V. and J. T. Hollibaugh. 1993. Coastal metabolism and the oceanic organic carbon balance. Rev. Geoph. 31: 75-89.

Smith, S. V., P. L. Jokiel (1978). Water composition and biogeochemical gradients in the Canton Atoll lagoon. Atoll. Res. Bull. 221: 15-53

Smith, S. V., F. Pesret (1974). Processes of carbon dioxide flux in the Fanning Island lagoon. Pac. Sci. 28: 225-245

Smith, S. V., P. L. Jokiel, G. S. Key (1978) Biogeochemical budgets in coral reef ecosystems. Atoll Res. Bull. 220: 1-11.

Smith, W. O. Jr. 1977. The respiration of photosynthetic carbon in eutrophic areas of the ocean. J. Mar. Res. 35:557-565.

Soholt, L. F. 1973. Consumption of primary production by a population of kangaroo rats (Dipodomys merriami) in the Mojave desert. Ecological Monographs 43: 357-376.

Sorokin, Y. 1983. The Black Sea, p. 253-292. *In* B. Ketchum [ed.], Estuaries and Enclosed Seas. Ecosystems of the World n° 26 Elvesier.

Soszka, G. J. The invertebrates on submerged macrophytes in three masurian lakes. Ekol. pol. **23**, 371-391 (1975).

Spencer, P. 1977. Carbon budgets and vertical zonation of atlantic reef corals, p. 391-396. *In* D. Taylor [ed.], Proceedings of the the third coral reef symposium, (I) Biology. Springer-Verlag.

Stanley, D. W. A carbon flow model of epipelic algal productivity in Alaskan Tundra Ponds. Ecology **57**, 1034-1042 (1976).

Strom, S. and N. Welschmeyer. 1991. Pigment specific rates of phytoplankton growthand microzooplankton grazing in the open subartic Pacific Ocean. Limnol. Oceanogr. 36:50-63.

Strugnell, R. G. & Pigott, C. D. Biomass, shoot-production and grazing of two grasslands in the Rwenzori National Park, Uganda. J. Ecol. **66**, 73-96 (1978).

Suess, E. 1980. Particulate organic carbon flux in the oceans: surface productivity and oxygen utilization. Nature 288: 260-263.

Svensson, B.H. and T. Rosswell. 1980. Energy flow through the sub-arctic mire at Stordalan. in: Ecology of sub-arctic mire (ed. M. Sonesson). Ecological Bulletin no. 30. Swedish Natural Science Research Council. Liber Tryck, Stockholm.

Tanaka Y (1991) Microbial decomposition of reed (Phragmites communis) leaves in a saline lake. Hydrobiologia 220: 119-129.

Tanner, E. 1980. J of Ecology 68: 833

Taylor BR, Parkinson D, Parsons WFJ (1989) Nitrogen and lignin content as predictors of litter decay rates: a microcosm test. Ecology 70: 97-104

Teal, J. 1962. Energy flown in the salt marsh ecosystem of Georgia. Ecology 43:615-624.

Tenhunen, J. D., Lange, O. L., Hahn, S., Siegwolf, R. & Oberbauer, S. F. in Arctic Ecosystems in a changing climate. An Ecophysiological perspective. (eds. Chapin III, F. Stuart, Jefferies, R. L., Reynolds, J. F., Shaver, G. R. & Svoboda, J.) 213-238 (Academic Press, New York, 1992).

Tertsching, W. 1989. Diel activity patterns and foraging dynamics of the sea urchin Tripneustes ventricosus in a tropical seagrass community and a reef environment (Virgin Islands). Mar. Ecol. 10: 3-21.

Thornton, D. D. 1971. The effect of complete removal of hippopotamus on grasslands in the Queen Elizabeth National Park, Uganda. East African Wildlife Journal 9: 47-55.

Tijseen, S. B. 1979. Diurnal oxygen rhythm and primary production in the mixed layer of the Atlantic Ocean at 20°N. Neth. J. Sea Res. 13: 79-84.

Tijssen, S. and A. Eijgenraam. 1982 Primary and Community production in the Southern Bight of the North Sea deduced from oxygen concentration variations in the srping of 1980. Neth. J. Sea Res. 16: 247-259.

Tilzer, M. M. Estimation of phytoplanton loss rates from daily photosynthetic rates and observed biomass changes in Lake Constance. J. Plank. Res. **6**, 309-324 (1984).

Twilley RR, Blanton LR, Brinson MM, Davis GJ (1985) Biomass production and nutrient cycling in aquatic macrophyte communities of the Chowan River, North Caroline. Aquat. Bot. 22: 231-252.

Twilley RR, Ejdung G, Romare P, Kemp M (1986) A comparative study of decomposition, oxygen consumption and nutrient release for selected aquatic plants occurring in an estuarine environment. Oikos 47: 190-198

Twilley, R., A. Lugo and C. Pattersen-Zuca. 1986. Litter production and turnover in basin mangrove forests in southwest Florida. Ecology 67:670-683.

Udell, H.F., J. Zamdsky and T.E. Doherry. 1969. Producticity and nutrient values of plants growing in the salt marshes of the Town of Hempstead. Bull. of the Torrey Bot. Club 96: 42-51

Urabe, J., M. Nakanishi and K. Kawabata. 1995. Contribution of metazoan plankton to the cycling of nitrogen and phosphorus in Lake Biwa. Limnol. Oceanogr. 40: 232-241.

Vadstein, O., Harkjerr, B., Jensen A., Olsen, Y. & Reinertsen, H. Cycling of organic carbon in the photic zone of a eutrophic lake with special reference to the heterotrophic bacteria. Limnol. Oceanogr. **34**, 840-855 (1989).

Valiela I, Wilson J, Buchsbaum R, Riesma C, Bryant D, Foreman K, Teal J (1984) Importance of chemical composition of salt marsh litter on decay rates and feeding by detritivores. Bull Mar Sci 35: 261-269

Van Cleve, K., R. Barney, and R. Schleuter. 1981. Evidence of temperature control of production and nutrient cycling in two interior Alaska black spruce ecosystems. Can. J. For. Res 11: 258-273

Van Cleve, K., Oliver, L., Schlentner, R., Viereck, L. A. & Dyrness, C. T. Productivity and nutrient cycling in taiga forests ecosystems. Can. J. For. Res. **13**, 747-766 (1983).

Van der Valk, A. and P. Attiwill. 1984. Decomposition of leaf and root litter of Aviccenia marina at Westernport Bay, Victoria, Autralia. Aquat.Bot. 18:205-221.

Van der Valk AG, Rhymer JM, Murkin HR (1991) Flooding and the decomposition of litter of four emergent plant species in a prairie wetland. Wetlands 11: 1-16

Van Hook, R. I. Energy and nutrient dynamics of spider and orthopteran populations in a grassland ecosystem. Ecol. Monogr. **41**, 1-25 (1971).

Van Wick, R. J. 1988. Ecological studies on Potamogeton pectinatus L. I. General characteristics, biomass production and life cycles under field conditions. Aquat. Bot. **31**, 211-258.

Varley, G. C. (1970). The concept of energy flow applied to a woodland community. In: Animal populations in relation to their food resources. (ed. Watson, A.) pp. 389-405 (10th BES Symp, Blackwell Scientific, Oxford).

Velimirov B. and M. Walenta-Simon. 1992. Seasonal changes in specific growth rates, production and biomass of bacterial community in the water colum above a Mediterranean seagrass system. Mar. Ecol. Prog. Ser. 80: 1237-248.

Vidal, M. 1991. Estudios sobre la dinámica de nutrientes en sistemas estuaricos:flujos sedimento-agua de nutrientes y oxígeno. Ph. D. Tesis. Universidad de Barcelona, Spain.

Wahbeh MI, Mahasneh AM (1985). Some aspects of decomposition of leaf litter of the seagrass Halophila stipulacea from the Gulf of Aqaba (Jordan) Aquat. Bot. 21: 237-244

Wallace, J. B. & O´Hop, J. Life on a fast pad: waterlily leaf beetle impact on water lilies. Ecology **66**, 1534-1544 (1985).

Wallentinus I. 1981. Chemical constituents of some baltic macroalgae in relation to environmental factors. Proc. Int. Seaweed Symp. 10: 363-370

Walsh, J. J. (1988). How much shelf production reaches the deep sea. In: Productivity of the ocean: present and past. Dahlem Workshop 53. Berlin.

Walsh I, Dymond J, Collier R (1988) Rates of recycling of biogenic components of settling particles in the ocean derived from sediment trap experiments. Deep-Sea Res 35:43-58

Wanders, J. B. W. The role of benthic algae in the shallow reef of Curaçao (Netherlands Antilles) III: The significance of grazing. Aquat. Bot. 3, 357-390 (1977).

Weisse, T., Müller H., Pinto-Coelho, M., Schweizer, A., Springmann, D. & Baldringer G. Response of the microbial loop to the phytoplankton spring bloom in a large prealpine lake. **35**, 781-794 (1990).

Welschmeyer, N.A., Lorenzen C, J. Chlorophyll budgets: zooplankton grazing and phytoplankton growth in a temperate fjord and the Central Pacific Gyres. Limnol. Oceanogr. 30, 1-21 (1985)

Wetzel, R. and P. Penhale. 1983. Photosynthesis, light response and metabolism of submerged macrophyte communities in the lower Chesapeake Bay, p. 50-107. *In* R.L. Wetzel [ed.], Structural and functional aspects of the ecology of submerged aquatic macrophyte communities in the lower Chesapeake Bay, vol. I. U.S. EPA Final Grant Report No. R 805974 and X003245-01 and Virginia Institute of Marine Science SRAMSOE 267, Gloucester Point, Virginia.

Whealer, B. D. & Shaw, S. C. Above-ground crop mass and species richness of the principal types of herbaceous rich-fen vegetation of lowland England and Wales. J. Ecol. **79**, 285-301 (1991).

White, D., T. Weiss, J. Trapani and L. Thien. 1978. Production and decomposition of the dominant salt marsh plants in Lousiana. Ecology 59: 751-759.

Whitfield, D. W. A. Energy inputs and ecological efficiencies on Truelove Lowland. In: Truelove Lowland, Devon Island, Canada: A High Arctic Ecosystem (ed. Bliss, L. C.) pp. 607-620 (University of Alberta Press, Edmonton, 1977).

Whittaker, R.H. Communities and Ecosystems (MacMillan, London, 1975).

Whittaker, R. H. and G. M. Woodwell. 1969. Structure, production and diversity of the oak pine forest at Brookhaven, New York. J. Ecol. 57: 155-174.

Wiegert, R. & Evans, F. Primary production and the disappearance of dead vegetation on an old field n southeastern Michigan. Ecology **45**, 49-63 (1964).

Wielgolaski, F. E. 1975. Functioning of Fennoscandian Tundra Ecosystems. In: Fennoscandian Tundra Ecosystems. Part 2: Animals and System Analyses (ed. Wielgolaski, F. E.) 300-326 (Springer-Heidelber).

Williams, S. L. and R. C. Carpenter. 1988. Nitrogen-limited primary productivity of coral reef algal turfs: potential contribution of ammonium excreted by Diadema antillarum. Mar. Ecol. Prog. Ser. 47: 145-152.

Wolff, M., V. Koch, and V. Isaac (2000) A trophic flow model of the Caeté mangrove estuary (north Brazil) with considerations for the sustainable use of its resources. Estuarine, Coastal, and Shelf Science 50:789-803

Woodwell, G. M. & Botkin, D. B. in Analysis of temperate forest ecosystems. Ecological studies 1 (ed. Reichle, D. E.), 281-302 (Springer-Verlag, New York, 1970).

Woodwell, G. M., R. A. Houghton, C. A. S. Hall, D. E. Whitney, R. A. Moll and D. W. Juers. 1979. The Flax Pond ecosystem study: the annual metabolism and nutrient budgets of a salt marsh. pp: 491-511. In: R. L. Jefferies and A. J. Davy (eds.), Ecological Processes in Coastal Environments. Symposium of the British Ecological Society. Blackwell Scientic, Oxford, England.

Yap, H., A. Montebon and R. Dizon.1994. Energy Flow and seasonality in a tropical coral reef flat. Mar. Ecol. Prog. Ser. 103:35-43.

Zak, D. R., D. Tilman, R. P. Parmenter, C. W. Rice, F. M. Fisher, J. Vose, D. Milchunas, C. W. Martin. 1994. Plant production and soil microorganisms in late-successional ecosystems: a continental study. Ecology 75: 2333-2347.

Zieman, J. C., G. Thayer, M. Robblee and R.Zieman. 1979. Production and export of seagrasses from a tropical bay, p. 21-33. *In* R. J. Livingston [ed.], Ecological Processes in Coastal and Marine Systems, Plenum Press.

Ziemann, D. A., P. K. Bienfag, K.W. Fulton-Bennett, W. Kottenmeier and L.D. Conquest. 1993. Carbon budget for the spring bloom in Auke Bay, Alaska. Mar. Biol. 115: 509-521.
